# Exploiting the potential of *in situ* forming liquid crystals: development and *in vitro* performance of long-acting depots for peptide drug thymosin alpha 1 subcutaneous administration

**DOI:** 10.1080/10717544.2025.2460708

**Published:** 2025-03-11

**Authors:** Mercedes Vitek, Alenka Zvonar Pobirk, Robert Roškar, Mirjam Gosenca Matjaž

**Affiliations:** a Department of Pharmaceutical Technology, Faculty of Pharmacy, University of Ljubljana, Ljubljana, Slovenia; b Department of Biopharmaceutics and Pharmacokinetics, Faculty of Pharmacy, University of Ljubljana, Ljubljana, Slovenia

**Keywords:** Glycerol monooleate, glycerol monolinoleate, hexagonal mesophases, microstructure, subcutaneous injection, UHPLC analysis, sustained release, patient adherence

## Abstract

The fast-growing filed of long-acting depots for subcutaneous (SC) administration holds significant potential to enhance patient adherence to treatment regimens, particularly in the context of chronic diseases. Among them, injectable *in situ* forming lyotropic liquid crystals (LCCs) consisting of hexagonal mesophases represent an attractive platform due to their remarkable highly ordered microstructure enabling the sustained drug release. These systems are especially relevant for peptide drugs, as their use is limited by their short plasma half-life and inherent poor stability. In this study, we thus aimed to exploit the potential of a liquid crystalline platform for the sustained release of peptide drug thymosin alpha 1 (Tα1), characterized by a short plasma half-life and with that associated twice-weekly SC administration regimen. We initially selected specified ingredients, with ethanol serving to reduce viscosity and stabilize the peptide drug Tα1, lecithin contributing to LCCs formation and stabilization, and glycerol monooleate or glycerol monolinoleate representing the hexagonal LCCs forming matrix material. The selected studied nonaqueous precursor formulations were characterized by suitable rheological properties for SC injection. A convenient and rapid *in situ* phase transition of precursor formulations to hexagonal LCCs, triggered by water absorption, was successfully accomplished *in vitro.* Notably, *in situ* formed LCCs demonstrated sustained release kinetics of the peptide drug Tα1 for up to 2 weeks of *in vitro* release testing, offering minimized dosing frequency and thus promoting patient adherence. In summary, the newly developed *in situ* forming liquid crystalline systems represent prospective injectable long-acting depots for SC administration of the peptide drug Tα1.

## Graphical abstract

**Figure:**
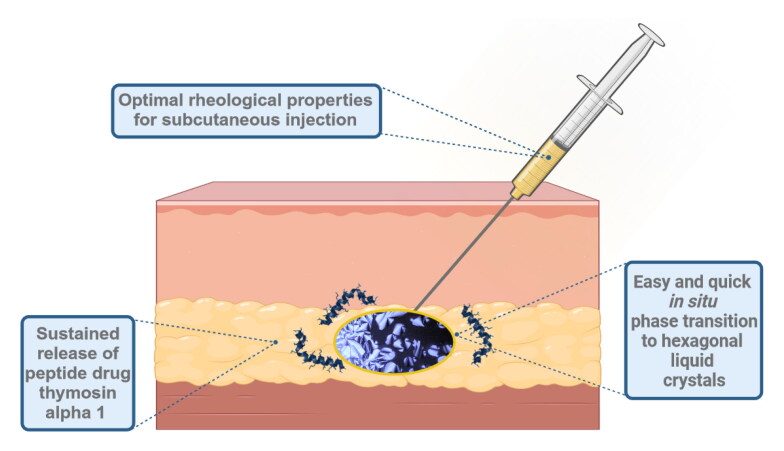

## Introduction

Patient adherence to a treatment regimen remains one of key challenges within the pharmaceutical community as it significantly influences therapeutic outcomes, especially in the treatment of chronic disorders and diseases (Bassand et al., 2022). Managing chronic illnesses demands strict adherence to a treatment regimen, which commonly includes multiple daily or weekly doses to maintain constant therapeutic levels of the drug in the human body. This is of particular importance for drugs susceptible to rapid *in vivo* clearance (Jindal et al., 2023). Yet, compliance with extended therapeutic regimen typically stands at approximately 50%, even in developed countries (Sabaté, 2003).

The fast-growing field of long-acting depot drug delivery systems for subcutaneous (SC) administration holds the potential to revolutionize the current treatment of chronic conditions (Nkanga et al., 2020; Dubbelboer & Sjögren, 2022; Rama & Ribeiro, 2023). Long-acting depots provide minimized dosing frequency and with that associated reduced side effects, while the SC delivery route enables quick and convenient self-administration. Ultimately, these benefits lead to improved patient adherence and associated therapeutic outcomes with reduced overall healthcare costs (van den Bemt et al., 2019; Li et al., 2021).

A plethora of different drug delivery technologies have been proposed for the formulation of long-acting depot drug delivery systems up to now (Chaudhary et al., 2019; Park et al., 2022; Lou et al., 2022). Simple oil-based solutions and suspensions were initially introduced. Following this, polymeric microspheres were utilized. Lately, *in situ* forming drug delivery systems have emerged as an attractive platform where the depot is formed upon injection due to a phase transition trigger such as body biological fluid, enzyme catalysis, physiological temperature, or pH (Jain & Jindal, 2024).

Among them, injectable *in situ* forming liquid crystalline systems have acquired a high appeal due to remarkable structural features, resulting in unique functionalities (Rahnfeld & Luciani, 2020; Sharma et al., 2023). Liquid crystalline mesophases are formed spontaneously by the self-assembly of amphiphilic lipids upon contact with aqueous environment providing a liquid crystalline gel with embedded drug molecules. The sustained drug release is a result of the slow drug diffusion through the network of nanochannels in the mesophases, whereby the channel size can be adjusted by changing the composition or other conditions (Shanmugam & Banerjee, 2011). Various mesophases can be formed corresponding to molecular shapes, local and global packing constraints, and the average interfacial mean curvature (Mezzenga et al., 2019). In accordance, lyotropic liquid crystals (LCCs) can be classified into lamellar, hexagonal, and cubic mesophases. For the development of long-acting depot drug delivery systems for SC administration hexagonal and cubic LCCs are of prime interest due to their highly ordered microstructure. Hexagonal mesophases are defined as cylindrical micelles packed in an infinite two-dimensional lattice arrangement of a hexagonal pattern, while cubic mesophases consist of three-dimensional structures formed by a continuous curved lipid bilayer with non-contacting aqueous channels (Chavda et al., 2022).

Hexagonal and cubic LCCs are thermodynamically stable colloidal systems and, owing to their high degree of order, they are less prone to fusion or aggregation as well as drug leakage when compared to other *in situ* forming drug delivery systems (Allegritti et al., 2024). In addition, their tunable properties concerning their microstructural organization enable relatively high loading and predictable release of small molecules as well as biomolecules such as proteins and peptides (Clogston & Caffrey, 2005; Chavda et al., 2022). In keeping with this, *in situ* forming LCCs as long-acting depot drug delivery systems are especially relevant for peptides as there is a great interest for the improvement of their pharmacokinetic properties (Tiwari et al., 2010; Wang et al., 2022). Namely, despite their high efficacy and low toxicity, their use is limited by their short plasma half-life due to significant metabolism by enzymes *in vivo* as well as their inherent poor physical and chemical stability. Given their immense therapeutic potential, market prospects, and economic value, there is a high demand for novel drug delivery systems that would effectively address the challenges described above (Gonella et al., 2022; Yang et al., 2022). Thymosin alpha 1 (Tα1), an immunomodulatory bioactive peptide with 28 amino acid residues, characterized by a short plasma half-life and with that associated twice-weekly SC administration regimen (Dominari et al., 2020), was chosen as a model peptide drug in this study.

Herein, we thus aimed to exploit the potential of liquid crystalline platform for development of patient-friendly *in situ* forming system for SC administration, designed to achieve sustained release of the peptide drug Tα1. Accordingly, our study prioritized the following main objectives: (i) optimal rheological properties of nonaqueous precursor formulation for SC injection, (ii) easy and quick *in situ* phase transition to hexagonal and/or cubic LCCs triggered merely by water absorption, (iii) sustained release kinetics of peptide drug Tα1 that would notably minimize its dosing frequency.

## Material and methods

### Materials

Ethanol (96% v/v) was provided by Pharmachem, Ljubljana, Slovenia. Lipoid^®^ S-100, soybean lecithin with not less than 94% (w/w) phosphatidylcholine content, was supplied from Lipoid GmbH, Ludwigshafen, Germany. According to the manufacturer’s specification the fatty acids of the two acyl groups of phosphatidylcholine are palmitic (15%), stearic (3%), oleic and isomers (12%), linoleic (62%), and α-linolenic (5%). Glycerol monooleate, type 40 (Peceol), and glycerol monolinoleate (Maisine^®^ CC) were obtained as a gift sample from Gattefossé SAS, Saint-Priest, France. According to the manufacturer’s specification, the first consists of monoglycerides (32–52%), diglycerides (30–50%) and triglycerides (5–20%) of mainly oleic (C_18:1_) acid and the latter consist of monoglycerides (32–52%), diglycerides (40–55%) and triglycerides (5–20%) of mainly linoleic (C_18:2_) and oleic (C_18:1_) acids. Tα1, a 28 amino acid sequence peptide, was purchased from Pure Peptides UK, Epsom, United Kingdom. Bidistilled water and phosphate buffered solution (PBS), respectively, were used throughout the experiments. All other chemicals and reagents were of analytical grade. For polarized light microscope examination, gelation time measurements, gelation test, and water uptake evaluation PBS with pH = 7.4 was used to mimic the subcutaneous environment at the injection site. As for the *in vitro* release testing, PBS with pH = 6.8 containing 5% (m/m) of ethanol was used as the release medium for the stability improvement of the peptide drug Tα1.

### Pseudoternary phase diagram construction

In order to determine the concentration ranges of the LCCs formation, four pseudoternary phase diagrams were constructed by a water titration method. Each diagram depicted three phases, with certain phases consisting of different combinations of ingredients. The first phase was composed of either a hydrotropic substance, i.e. ethanol, or a mixture of a hydrotropic substance and an amphiphile, i.e. ethanol and lecithin with a mass ratio of 1/1. The next phase was designated as the lipid phase consisting of glycerol monooleate or glycerol monolinoleate. Finally, the third vertex of the diagram was represented by the hydrophilic phase.

Nine dilution lines were constructed for each diagram, and the starting point of each line denoted a precursor formulation. All precursor formulations were thus composed of ethanol or ethanol-lecithin mixture and glycerol monooleate or glycerol monolinoleate with mass ratios ranging from 90/10 to 10/90%.

For the titration process, each precursor formulation was slowly titrated with aliquots of the hydrophilic phase and stirred at room temperature for a sufficient time to obtain equilibrium. Subsequently, samples were checked for homogeneity, consistency, and appearance. Homogeneous, highly viscous, and opaque samples were characterized as LCCs.

Next, samples visually identified as LCCs were further checked under a polarized light microscope at 25 °C and 37 °C for the presence of liquid crystalline mesophases. Based on the obtained results, eight precursor formulations were then selected for further characterization. The composition of these selected studied precursor formulations is detailed in Table 1, while the preparation procedure is outlined in the following section.

**Table 1.** Composition of the studied precursor formulations.

	(E/L)Go50	(E/L)Gl50	(E/L)Go60	(E/L)Gl60	(E/L)Go70	(E/L)Gl70	(E/L)Go80	(E/L)Gl80
Ethanol	25	25	30	30	35	35	40	40
Lecithin	25	25	30	30	35	35	40	40
Glycerol monooleate	50	–	40	–	30	–	20	–
Glycerol monolinoleate	–	50	–	40	–	30	–	20

The letters in the name of each system indicate ethanol – E, lecithin – L, glycerol monooleate – Go, glycerol monolinoleate – Gl, and the number indicates content of ethanol/lecithin mixture with a mass ratio of 1/1.

### Sample preparation

Unloaded precursor formulations were prepared by mixing appropriate amounts of ethanol or ethanol-lecithin mixture and glycerol monooleate or glycerol monolinoleate for a sufficient time to form a homogeneous system. In the case of Tα1-loaded precursor formulations containing 1.6 mg/g of peptide drug (i.e. dose of reference medicine) (Dominari et al., 2020), Tα1 was first dissolved in ethanol or ethanol-lecithin mixture. The resulting solution was then mixed with appropriate amount of glycerol monooleate or glycerol monolinoleate for a sufficient time until a homogeneous system was obtained. Both unloaded and Tα1-loaded precursor formulations were prepared at room temperature.

### Polarized light microscopy

Polarized light microscopy (PLM) was used for phase transition analysis of the samples from the pseudoternary phase diagram construction as well as the gelation test. In the former, samples that were identified as LCCs based on macroscopic examination of mixtures formed along dilution lines of the pseudoternary phase diagrams were examined at 25 °C and 37 °C to assess phase transition and presence of liquid crystalline mesophases, respectively. In the latter, phase transition of precursor formulations upon contact with PBS at predetermined time points (1, 6, 12, 24, 36, 48 and 72 hours, and 7, 10 and 14 days) was examined at 37 °C. PLM was performed using a CX31-P Upright Microscope (Olympus, Tokyo, Japan). The magnification was 40×.

### Gelation time measurements

Gelation time is the time required for a precursor formulation to convert into an *in situ* formed gel upon contact with an excess aqueous medium. Within the scope of gelation time measurements, 0.5 mL of each precursor formulation was injected with a 25-gauge needle into 5 mL of PBS preheated at 37 °C. The time upon contact of a liquid transparent precursor formulation with the aqueous medium until complete transformation into an opaque *in situ* formed gel was recorded as the gelation time (Mei et al., 2017). For each precursor formulation, the measurement was performed in triplicate.

### Gelation test

The ability to form and maintain an *in situ* formed gel of a precursor formulation upon contact with excess aqueous medium for a prolonged period of time was evaluated based on the macroscopic appearance of *in situ* formed gels at predetermined time points (1, 6, 12, 24, 36, 48 and 72 hours, and 7, 10 and 14 days). In addition, for better visualization, *in situ* formed gels in vials immediately after injection were also photographed. 0.5 mL of each precursor formulation was injected with a 25-gauge needle into a 10 mL vial with 5 mL of PBS preheated at 37 °C. To note, for every time point, each precursor formulation was injected into a separate vial. During the whole testing period samples were stored in an orbital shaker-incubator ES-20 (SIA Biosan, Riga, Latvia) set at 50 rpm and 37 °C. At each time point, PBS on the surface of each *in situ* formed gel was cautiously wiped off. Subsequently, *in situ* formed gels were observed visually for homogeneity, consistency, and appearance (Ki et al., 2014; Mei et al., 2018). In addition, phase transition analysis of *in situ* formed gels at 37 °C using a polarized light microscope was performed at each time point.

### Water uptake evaluation

To explore the swelling behavior of *in situ* formed gels, their water uptake was monitored by gravimetric analysis according to a standard protocol (Mei et al., 2018) at predetermined time points (1, 6, 12, 24, 36, 48 and 72 hours, and 7, 10 and 14 days). In keeping with this, the time point at which equilibrium with water was reached and the water maximum absorption, denoted as water capacity, was also determined. 0.5 g of each precursor formulation was injected with a 25-gauge needle into a 10 mL vial with 5 mL of PBS preheated at 37 °C. During the whole testing period samples were stored in an orbital shaker-incubator ES-20 (SIA Biosan, Riga, Latvia) set at 50 rpm and 37 °C. Experiments were performed in triplicate. At each time point, the respective masses were determined and the percentage of water uptake was calculated from Eq. (1), where M_vg_ represents mass of a vial together with *in situ* formed gel, M_v_ represents mass of a vial alone, M_vpp_ represents mass of a vial together with PBS and precursor formulation, and M_vp_ represents the mass of a vial together with PBS.

(1) $$W\%=\left(\frac{\text{Mvg}-\text{Mv}}{\text{Mvpp}-\text{Mvp}}-1\right)\times 100\%$$

### Differential scanning calorimetry

Differential scanning calorimetry (DSC) measurements were carried out for individual compounds (i.e. ethanol, lecithin, glycerol monooleate, glycerol monolinoleate, and bidistilled water) and *in situ* formed gels after reaching equilibrium with the water. DSC was performed to analyze intermolecular interactions and water state within samples. A DSC 1 differential scanning calorimeter (Mettler Toledo, Greifensee, Switzerland) was used. Approximately 10 mg of the sample was accurately weighed into a small aluminum pan and sealed. An empty sealed pan was used as a reference. Nitrogen with a flow rate of 50 mL/min was used as a purge gas. One cooling and one heating scan were recorded during each analysis. Samples were cooled from 20 °C to −80 °C, kept at −80 °C for 5 min, and heated to 140 °C. The cooling and heating rate was 5 K/min.

### Rheological measurements

The rheological behavior of precursor formulations and *in situ* formed gels after reaching equilibrium with water was characterized using a Physica MCR 301 rheometer equipped with RheoCompass software (Anton Paar GmbH, Graz, Austria). Rotational tests were conducted at 25 ± 0.1 °C for precursor formulations and at 37 ± 0.1 °C for *in situ* formed gels after reaching equilibrium with water. Experiments were performed in duplicate. Rotational measurements were carried out to determine the viscosity (η), which was calculated according to Eq. (2), where τ is the shear stress and γ̇ is the shear rate.

(2) η=τ/γ˙

Oscillatory tests were employed to define the storage (elastic; G′) and loss (viscous; G″) moduli of *in situ* formed gels after reaching equilibrium with water at 37 ± 0,1 °C. They were calculated using Eqs. (3) and (4), respectively, where τ is the shear stress, *γ* is the deformation, and δ is the phase shift angle.

(3) $$G^{\prime }=(\tau /\gamma )\times \text{cos}\delta$$

(4) $$G^{\prime \prime }=(\tau /\gamma )\times \text{sin}\delta$$

In addition, complex viscosity (η*) was calculated according to Eq. (5), where τ is the shear stress, γ is the deformation, and ω is the angular frequency.

(5) η∗=τ/(γ×ω)

Rotational tests were performed using a cone and plate measuring system CP50-2 (cone diameter 49.961 mm, cone angle 2.001°, sample thickness 0.209 mm). The shear rate ranged from 1 s^−1^ to 100 s^−1^. For the oscillatory tests, the stress sweep measurements were carried out at a constant frequency of 10.0 s^−1^ to determine the linear viscoelastic region. Afterward, the oscillatory shear measurements were performed as a function of frequency (0.1–100 s^−1^) at a small stress (0.1%) chosen within the linear region to provide the least disturbance of the microstructure.

### In vitro release testing

A membraneless model, which enables direct contact between *in situ* formed gel and release medium, was applied for *in vitro* release testing. 1 g of Tα1-loaded precursor formulation containing 1.6 mg/g of peptide drug (i.e. dose of reference medicine) (Dominari et al., 2020) was injected with a 25-gauge needle directly into 15 mL of release medium preheated at 37 °C. Considering physiological conditions after SC administration and chemical stability of peptide drug Tα1, PBS (pH = 6.8) containing 5% (m/m) of ethanol was selected as the most appropriate release medium for the test completion in 2 weeks at 37 °C. At predetermined time points (1, 6, 12, 24, 36, 48 and 72 hours, and 7, 10 and 14 days) 1 mL ­aliquots of release medium were withdrawn and replaced by an equal volume of fresh preheated receptor medium to keep the volume constant. During the whole testing period samples were stored in an orbital shaker-incubator ES-20 (SIA Biosan, Riga, Latvia) set at 50 rpm and 37 °C. Experiments were performed in quadruplicate. The medium that was taken at each time point was analyzed quantitatively by ultra-high performance liquid chromatography (UHPLC) analysis described below. The cumulative amount of the released peptide drug Tα1 (Q_t_) was plotted as a function of time and calculated according to Eq. (6) where c_t_ is the peptide drug Tα1 concentration of receptor medium at each sampling time, V_rm_ is the volume of receptor medium, c_i_ is the peptide drug Tα1 concentration at previous sampling times, and V_i_ is the sampling volume.

(6) $$Q_{t}=\left(c_{t}\times V_{\text{rm}}+\overset{t-1}{\underset{i=0}{\sum }}c_{i}\times V_{i}\right)$$

### UHPLC analysis

An Infinity 1290 ultra-high performance liquid chromatograph (Agilent Technologies, Santa Clara, CA, USA) equipped with a diode array detector with a high-sensitivity Max-Light cartridge cell (60 mm) and an EZChrom acquisition system was used. Chromatographic separation was performed on a reversed-phase Synergi Hydro column 150 × 4.6 mm, 4 µm particle size (Phenomenex, Torrance, CA, USA) at 40 °C. The mobile phase consisted of solvent A: 0.1% H_3_PO_4_ and solvent B: acetonitrile with a gradient elution of 12.0% to 16.5% solvent B in 12 min at a flow rate of 1 mL/min. The total run time was 14 min. The injection volume was 10 μL, and a detection wavelength of 214 nm was selected. The method was validated in terms of selectivity (no interference at the retention time of the Tα1), linearity (*R*^2^ = 1.000 in the concentration range between 1 and 100 mg/L), precision (RSD < 5%) and accuracy (10 0 ± 5%). Tα1 was stable in the samples for at least 4 days when 5% ethanol was added to the solution.

### Circular dichroism spectroscopy

Circular dichroism (CD) measurements were performed using a Chirascan CD spectrometer equipped with a Peltier temperature controller (Applied Photophysics Ltd, London, United Kingdom). CD spectra were recorded in a 1-mm quartz cell (Hellma GmbH & Co, Müllheim, Germany) at 37 °C using 1-nm step, 1-nm bandwidth and 1-s sampling. The secondary structure of the peptide drug Tα1 was analyzed by scan measurement in the range from 200 nm to 260 nm. The results are the average of three scans.

### Data and statistical analysis

The data and statistical analysis were performed with GraphPad Prism 10.2.0. All results, unless stated otherwise, were expressed as mean ± standard deviation (SD).

## Results and discussion

### Pseudoternary phase diagram construction

**Figure 1.**
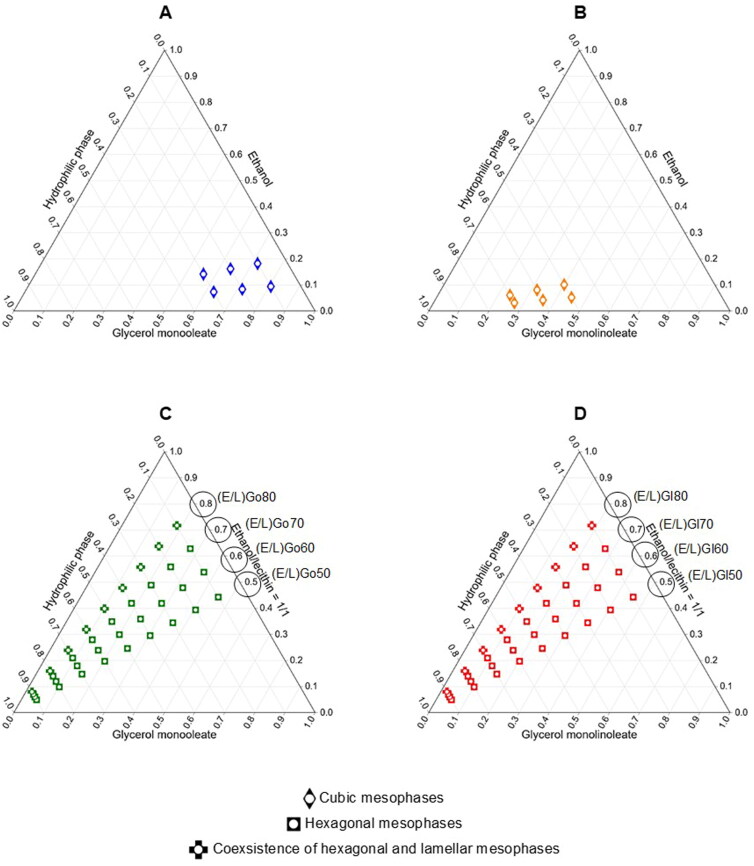
Pseudoternary phase diagrams of the systems composed of (A) ethanol/glycerol monooleate/hydrophilic phase, (B) ethanol/glycerol monolinoleate/hydrophilic phase, (C) ethanol/lecithin/glycerol monooleate/hydrophilic phase, and (D) ethanol/lecithin/glycerol monolinoleate/hydrophilic phase. White circles are showing areas of existence of LCCs as determined based on macroscopic anaylsis at 25 °C. Color diamonds, squares, and crossess are showing areas of existence of LCCs as determined based on PLM anaylsis at 25 °C and/or at 37 °C. The selected studied precursor formulations are marked with a black circle.

When developing a novel drug delivery system based on LCCs, phase behavior of systems with precisely defined components can be investigated using ternary or pseudoternary phase diagrams. In this study, four pseudoternary phase diagrams were constructed for the systems containing ethanol/glycerol monooleate/hydrophilic phase (Figure 1(A)), ethanol/glycerol monolinoleate/hydrophilic phase (Figure 1(B)), ethanol/lecithin/glycerol monooleate/hydrophilic phase (Figure 1(C)), and ethanol/lecithin/glycerol monolinoleate/hydrophilic phase (Figure 1(D)). Ethanol had a role of a hydrotropic substance for viscosity reduction (Ferreira et al., 2020) as well as peptide drug Tα1 stabilization. Lecithin was chosen as a biocompatible amphiphile capable of formation and stabilization of various LCCs mesophases depending on the remaining amphiphiles in the mix (Gosenca et al., 2013). Glycerol monooleate and glycerol monolinoleate were selected as hexagonal and/or cubic mesophases-forming amphiphilic lipids. They possess excellent biocompatible and biodegradable characteristics due to the presence of ester bonds in their structure, which undergo lipolytic degradation by endogenous lipases after SC injection (Zhang et al., 2020). Hydrophilic phase represented aqueous environment of the SC tissue.

Macroscopic analysis of the systems formed across the constructed pseudoternary phase diagrams was initially performed. Different proportions and type of components resulted in formation of distinct systems. Transparent or semi-transparent and viscous gel-like systems were identified as LCCs. As our focus was on the formation of liquid crystalline mesophases, we did not explore in detail other regions of the diagrams where nonhomogeneous systems, coarse emulsions, or microemulsions were present. Pseudoternary phase diagram study showed that absence or presence of lecithin had a key influence on the formation of LCCs, while the type of lipid did not show any significant effect. Namely, in the diagrams from Figure 1(A and B) employing only ethanol, lipid, and hydrophilic phase, a considerably smaller region of LCCs formation was observed compared to the diagrams from Figure 1(C and D) utilizing ethanol/lecithin mixture, lipid, and hydrophilic phase. More specifically, in the case of the diagram from Figure 1(A), adding hydrophilic phase at a level of 10–30% to precursor formulations containing 10–20% of ethanol resulted in the formation of LCCs. In parallel, a similarly small region of LCCs was distinguished in the diagram from Figure 1(B), where addition of hydrophilic phase ranging from 50 to 70% to precursor formulations with 10–20% of ethanol led to LCCs formation. On the other hand, in the case of the diagrams from Figure 1(C and D), precursor formulations containing 50–80% of the ethanol/lecithin mixture formed a large region of LCCs after hydrophilic phase was added in the range of 10–90%. When comparing the systems across all diagrams from Figure 1, it is important to note that the LCCs formed in the diagrams from Figure 1(C and D) exhibited favorable macroscopic characteristics such as homogeneity and high viscosity.

### Phase transition analysis within pseudoternary phase diagram construction

**Figure 2.**
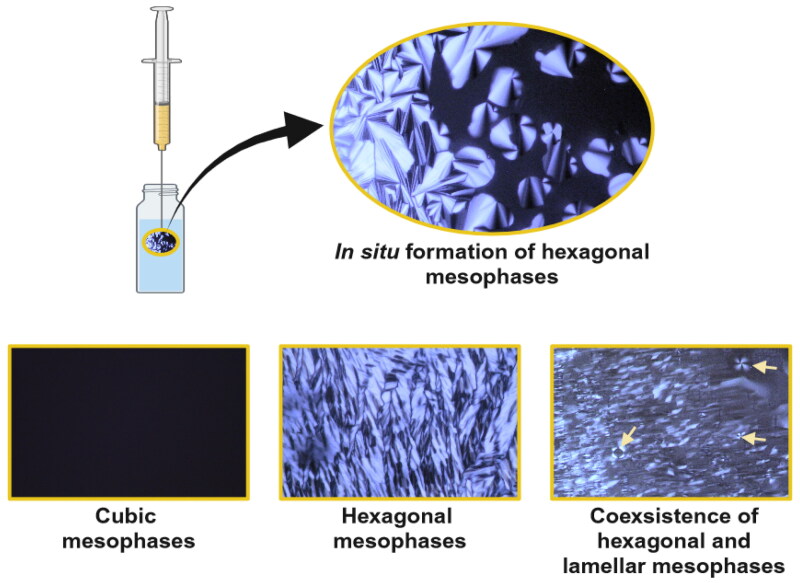
Representative PLM photomicrographs obtained through phase transition analysis within pseudoternary phase diagram construction demonstrating cubic mesophases, hexagonal mesophases, and coexsistence of hexagonal and lamellar mesophases at 37 °C. The Maltese crosses are indicated by the arrows.

Systems identified as potential LCCs based on macroscopic analysis, utilizing pseudoternary phase diagrams, underwent subsequent microscopic analysis using polarized light microscope at room (25 °C) and body (37 °C) temperature. The acquired observations are schematically presented in Figure 1, while the representative photomicrographs are depicted in Figure 2. PLM is one of the most commonly used methods for investigating LCCs mesophases, offering valuable insight into their molecular organization and phase transitions. When subjected to polarized light, anisotropic systems such as lamellar and hexagonal LCCs exhibit characteristic birefringent pattern. Maltese crosses together with oily streaks denote the presence of lamellar mesophases, while fan-like textures indicate formation of hexagonal mesophases. A dark background, with no birefringence, suggests the presence of cubic mesophases, known for their isotropic liquid behavior (Manaia et al., 2017; Zhang et al., 2020).

First, evaluation of the systems was performed at a temperature of 25 °C. In the case of the systems from the first and the second diagram, the black view under polarized microscope pointed to the presence of cubic LCCs being in good agreement with the investigation by Mei et al. (2018). In contrast, the phase behavior of the systems depicted in the third and fourth diagram exhibited distinctive characteristics of LCCs, confirming that lecithin has a key function in formation of these LCCs. Namely, numerous and pronounced fan-like textures started to emerge in the dark background from precursor formulations containing 50%, 60%, and 70% of the ethanol/lecithin mixture, respectively, independent of the hydrophilic phase content. This observation is imperative as it draws us to two important findings. Firstly, it indicates the presence of hexagonal LCCs and in some areas coexistence with cubic LCCs; nevertheless, hexagonal mesophases were strongly prevailing. Secondly, it implies that absorption of only a small amount of hydrophilic phase was necessary for these precursor formulations to form hexagonal LCCs *in situ*. Further, mixed phases of hexagonal LCCs together with lamellar LCCs were formed from precursor formulations containing 80% of the ethanol/lecithin mixture, respectively, but only after a larger addition of hydrophilic phase, i.e. 40–90%. It should be noted that, for precursor formulations containing 80% of the ethanol/lecithin mixture, fan-like textures were less numerous and pronounced as well as that individual Maltese crosses were also observed.

Further, comparable results were obtained at a temperature of 37 °C for all four diagrams. The only exception was that precursor formulations from the third and the fourth diagram containing 80% of the ethanol/lecithin mixture already showed fan-like textures along with Maltese crosses when the hydrophilic phase content reached 10%. This indicates that higher temperature induces, to some extent, the formation of hexagonal LCCs together with lamellar LCCs, which is desirable for SC administration.

Based on all the findings obtained from the pseudoternary phase construction (i.e. macroscopic appearance) and the corresponding phase behavior analysis (i.e. microstructure), eight precursor formulations, capable of *in situ* phase transition to hexagonal LCCs upon addition of water, were selected for further characterization studies. The composition of the selected studied precursor formulations is reported in Table 1 and highlighted in Figure 1.

### Gelation time measurements

Gelation time denotes the time required for a precursor formulation to transform into an *in situ* formed gel when exposed to excess aqueous medium (Mei et al., 2017). A rapid sol-gel transition is desired to minimize the possibility of initial burst release as *in situ* formation of gel retards the release of the incorporated drug. At room temperature, all precursor formulations were clear with good fluidity, but upon contact with aqueous medium heated to 37 °C, they quickly lost their flowability. Among all precursor formulations, a very short gelation time of a few seconds was measured for (E/L)Go50 (2.8 seconds), (E/L)Gl50 (2.5 seconds), (E/L)Go60 (4.1 seconds), and (E/L)Gl60 (3.6 seconds). Further, a slightly longer gelation time was measured for (E/L)Go70 (14.1 seconds) and (E/L)Gl70 (13.3 seconds). The longest sol-gel transition time was determined for (E/L)Go80 (70.0 seconds) and (E/L)Gl80 (45.4 seconds). These results indicate that all precursor formulations would spontaneously transform into an *in situ* gel at the site of administration upon exposure to the physiological fluid. However, it can be observed that there are certain differences among precursor formulations which are most likely related to their composition. Namely, increasing glycerol monooleate and glycerol monolinoleate content, respectively, leads to a decrease in gelation time, which is also consistent with the literature data (Mei et al., 2018). The phenomenon can be attributed to glycerol monooleate and glycerol monolinoleate being amphiphilic lipids capable of forming hexagonal LCCs. Consequently, they have the ability to rapidly induce the formation of these highly viscous mesophases.

**Table 2.** DSC thermodynamic parameters (i.e. crystallization temperature and crystallization enthalpy) of the cooling cycle of *in situ* formed gels after reaching equilibrium with water.

	T_c1_ (°C)	ΔH_c1_ (J/g)	T_c2_ (°C)	ΔH_c2_ (J/g)
(E/L)Go50	–18.5	3.4	–32.7	7.9
(E/L)Gl50	–26.0	8.9	–43.1	3.2
(E/L)Go60	–24.1	5.0	–42.4	10.2
(E/L)Gl60	–16.2	5.6	–35.5	16.1
(E/L)Go70	–23.5	24.6	–43.4	18.2
(E/L)Gl70	–24.0	8.9	–44.7	12.3
(E/L)Go80	–22.6	99.2	–	–
(E/L)Gl80	–22.8	72.1	–	–

### Gelation test

**Figure 3.**
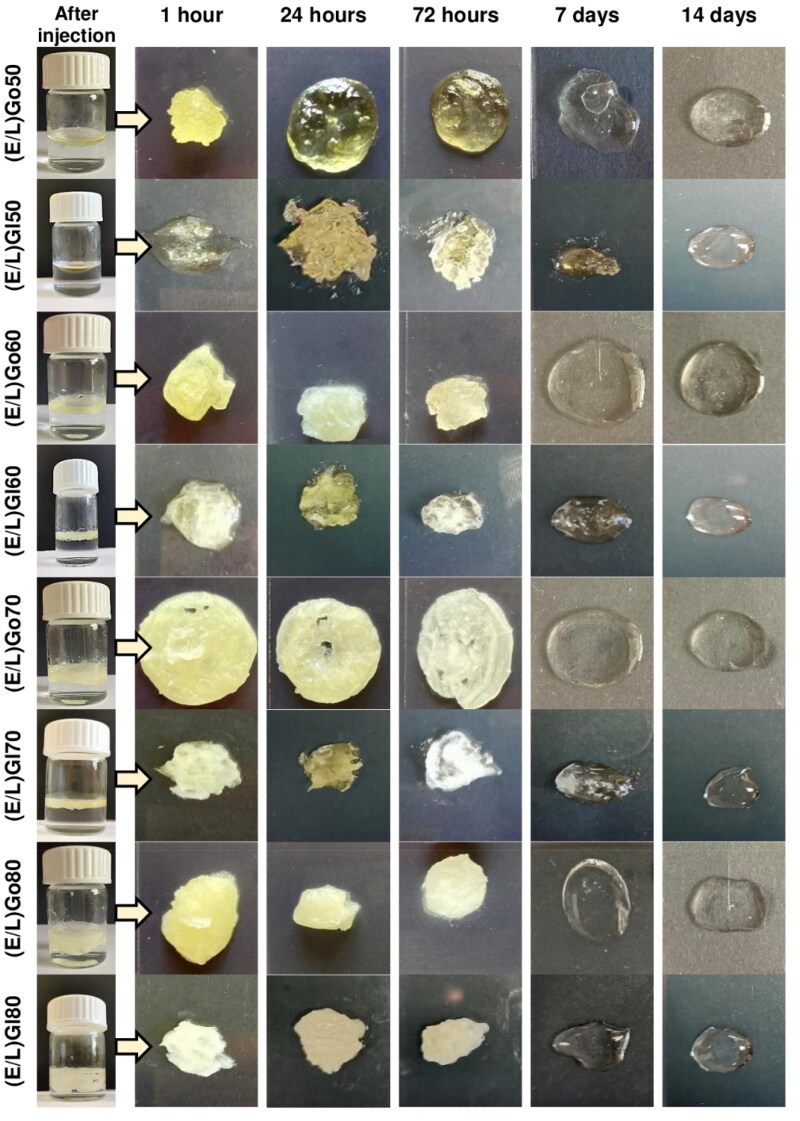
Morphology of *in situ* formed gels from precursor formulations upon contact with excess aqueous medium within the scope of gelation test at selected predetermined time points. In addition, for better visualization, *in situ* formed gels in vials immediately after injection are also shown.

The ability of a precursor formulation to form and maintain an *in situ* formed gel upon injection into excess aqueous medium heated to 37 °C was evaluated within gelation test. Figure 3 shows the visual appearance of *in situ* formed gels at selected predetermined time points, namely, immediately after injection, the initial and final time assessment points (1 hour and 14 days), the time points when equilibrium with water was established (24 and 72 hours), and the time point showing the most prominent morphological changes of *in situ* formed gels were observed (7 days). In addition, for better visualization, *in situ* formed gels in vials immediately after injection are also illustrated. Supplementary Figure S1 provides visual representation of *in situ* formed gels at other predetermined time points, namely 6, 12, 36, 48 hours, and 10 days.

The intensity of color of all *in situ* formed gels was the highest at the first time point, i.e. 1 hour, due to the thorough uptake of the aqueous medium, which began immediately upon contact with it. The most compact forms with noticeably low quantity of uptook aqueous medium were formed by (E/L)Go50 and (E/L)Gl50, which, unlike the milky yellow (E/L)Go80 and (E/L)Gl80, were bright yellow. The least firm and visibly the biggest volumes of *in situ* formed gels were observed for (E/L)Go80 and (E/L)Gl80, as they uptook high quantity of aqueous medium. (E/L)Go60, (E/L)Gl60, (E/L)Go70, and (E/L)Gl70 typically represented an ‘intermediate stage”. They combined both clear and cloudy areas in color, and their consistency was softer than that of (E/L)Go50 and (E/L)Gl50 and firmer than that of (E/L)Go80 and (E/L)Gl80. With the exception of less intense color and slower process of swelling, after 6, 12, 24, 36, 48, and 72 hours, no significant changes in macroscopic appearance of all *in situ* formed gels were observed. However, after 7 days notable changes in color and consistency were detected for all *in situ* formed gels as they started to liquefy and decrease in their size. Over the following days, the process of liquification and erosion was slowly progressing. Interestingly, at the last time point, i.e. 14 days, remaining *in situ* formed gels of (E/L)Go80 and (E/L)Gl80 settled at the bottom of the vials, while remainings of the other systems were still floating.

When comparing all precursor formulations, due to the yellow color of glycerol monooleate and glycerol monolinoleate, respectively, gels containing more lipid phase were more yellow. But what seems to be of major importance, we found that *in situ* formed gels with a higher lipid content were more compact and eroded more slowly, while *in situ* formed gels containing a higher amount of the ethanol/lecithin mixture were softer and degraded faster.

### Phase transition analysis within gelation test

**Figure 4.**
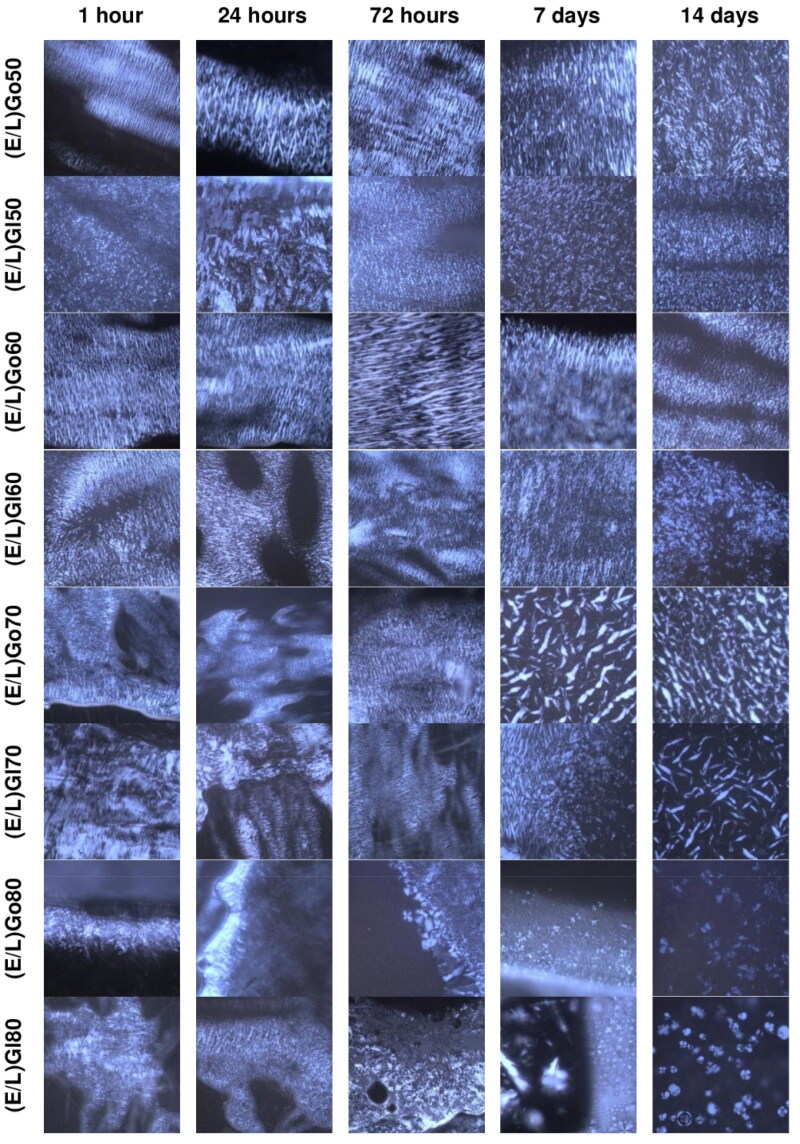
Representative PLM photomicrographs of *in situ* formed gels from precursor formulations upon contact with excess aqueous medium obtained through phase transition analysis within gelation test at selected predetermined time points at 37 °C.

As the microstructure of *in situ* formed gel is a crucial factor influencing the drug release kinetics, the phase transition of precursor formulations upon contact with aqueous medium was monitored using PLM at 37 °C. The analysis was performed at the same time points as the gelation test was carried out. Photomicrographs obtained at the selected predetermine time points, i.e. 1, 24, 72 hours, and 7 and 14 days (see chapter Gelation test) are shown in Figure 4. Supplementary Figure S2 provides photomicrographs taken at other predetermined time points, namely 6, 12, 36, 48 hours, and 10 days. The phase changes at post-hydration time of precursor formulations with excess aqueous medium revealed dynamic phase transitions, which were caused by rearrangement of molecules within precursor formulations.

These microstructural changes can be understood in terms of aqueous self-assembly of amphiphile mixtures explained by their critical packing parameter (CPP). Namely, the self-assembly of single amphiphiles in aqueous medium is driven by a balance between the hydrophobic interactions of the tails and the geometrical packing constraints of the polar head groups. These factors are expressed as CPP = *v/al*, where *v* is the volume of the hydrophobic tail, *a* is the polar head group area, and *l* is the hydrophobic tail length of the amphiphilic molecule. As a guideline, amphiphiles with a CPP ∼1 usually self-assemble into lamellar LCCs (Engström & Engström, 1992), a CPP of ∼1.3 is characteristic for bicontinuous cubic mesophases, while amphiphiles with a CPP ∼1.7 form inverted hexagonal mesophases (Larsson, 1989).

In regard to our results, clearly visible and numerous fan-like textures emerging from dark background, were observed for (E/L)Go50, (E/L)Gl50, (E/L)Go60, (E/L)Gl60, (E/L)Go70, and (E/L)Gl70 at the first time point of the assessment and they persisted until the conclusion of the analysis. It appears that hexagonal LCCs were quickly formed from these precursor formulations and that their microstructure was preserved until the final time point of the analysis. These findings can be attributed to the high content of glycerol monooleate and glycerol monolinoleate, respectively, in these precursor formulations. Namely, upon contact with aqueous medium, the polar head groups of the amphiphilic lipid from precursor formulations begin to move more freely. Consequently, these movements induce disorder in the hydrophobic chain of the amphiphilic lipid, leading to an increase of volume of the hydrophobic tail – *v*. However, the cross-sectional area of the polar head groups stays constant due to the strong hydrogen bonding. Therefore, CPP value increases as *v* increases and the polar head group area – *a* and the hydrophobic tail length of the amphiphilic molecule – *l* remain constant, thereby facilitating phase transition to hexagonal mesophases (Borgheti-Cardoso et al., 2015; Ferreira, 2022).

When looking at (E/L)Go80 and (E/L)Gl80, hexagonal LCCs were also mainly present at all time points of the assessment. However, it should be noted that here fan-like structures were less pronounced. In addition, Maltese crosses indicative of lamellar LCCs were observed at the initial time point for (E/L)Go80 and (E/L)Gl80, with their presence slowly increasing throughout the analysis. Given that these precursor formulations contained a high content of lecithin/ethanol mixture, its effect was reflected in the resulting mesophases. Namely, a CPP value for lecithin, specifically for phosphatidylcholine as its main component, ranges from 0.5 to 1, meeting the requirement for bilayer formation of lamellar mesophases. Furthermore, ethanol molecules intercalated within phospholipid bilayers of lecithin additionally contributed to the lipid bilayer fluidity (Mkam Tsengam et al., 2022). As a result, in the case of the abovementioned precursor formulations, the self-assembly of lamellar mesophases was also observed along with formation of hexagonal mesophases.

### Water uptake evaluation

**Figure 5.**
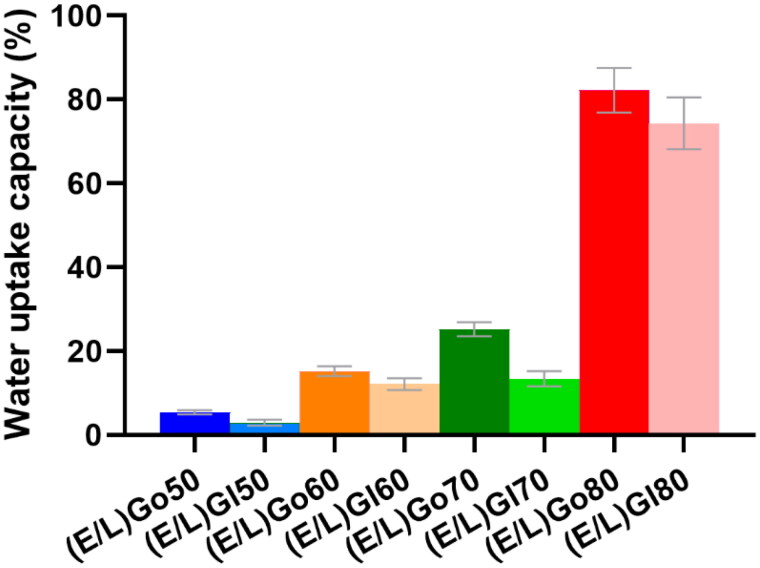
Water uptake capacity of *in situ* formed gels at equilibrium with water. Data are expressed as mean ± SD (*n* = 3).

Swelling behavior of *in situ* formed gels is another important characteristic influencing the drug release behavior. Therefore, their water uptake kinetics was evaluated at predetermined time points at temperature of 37 °C. The water uptake was monitored over time until equilibrium with water was reached. The determined value represented the maximum water absorption, referred to as water uptake capacity, shown in Figure 5.

The obtained results showed that the water uptake of all *in situ* formed gels increased rapidly in the first hour upon contact with excess aqueous medium and then gradually leveled off. The equilibrium water absorption for (E/L)Go50, (E/L)Gl50, (E/L)Go60, (E/L)Gl60, and (E/L)Gl70 was determined at 24 hours, while for (E/L)Go70, (E/L)Go80, and (E/L)Gl80 the equilibrium with water was reached after 72 hours. When considering the water capacities of *in situ* formed gels, the obtained data indicated that the chosen lipid played a pivotal role in their swelling behavior. The lowest water capacity was determined for (E/L)Go50 (5.4%) and (E/L)Gl50 (2.9%) consisting of the highest proportion of glycerol monooleate and glycerol monolinoleate, respectively. Slightly higher water capacities were observed for (E/L)Go60 (15.2%), (E/L)Gl60 (12.1%), and (E/L)Gl70 (13.4%). These results are in good agreement with phase transition analysis within gelation test, where it has been shown that fan-like textures, indicating hexagonal LCCs, are continually present in these *in situ* formed gels. According to the literature, water channels within hexagonal mesophases are closed to the external environment, hence water diffusion is retarded (Chavda et al., 2022). Further, moderately higher water capacity was determined for (E/L)Go70 (25.2%), while (E/L)Go80 (82.2%) and (E/L)Gl80 (74.3%) stood out with the highest water capacity. Again, these results correlate well with phase transition analysis within gelation test, revealing that in addition to hexagonal LCCs, lamellar mesophases are also present in (E/L)Go80 and (E/L)Gl80. It is known that lamellar LCCs usually absorb more water (Alfutimie et al., 2015). When looking at all the results together, another interesting finding can be observed. Namely, *in situ* formed gels containing glycerol monooleate appeared to absorb a higher amount of water compared to those containing glycerol monolinoleate. This overall trend is important to note, as it seems to be also reflected in the results of the *in vitro* release testing presented later in the study.

### Differential scanning calorimetry

DSC analysis was performed to elucidate intermolecular interactions and water state within *in situ* formed gels after reaching equilibrium with water. Evaluation was performed based on the crystallization (T_c_) and melting (T_m_) temperatures visible in the crystallization and the melting curves as well as the enthalpies of crystallization (ΔH_c_) and melting (ΔH_m_) derived by integrating the areas under the corresponding peaks in the DSC thermograms.

**Figure 6.**
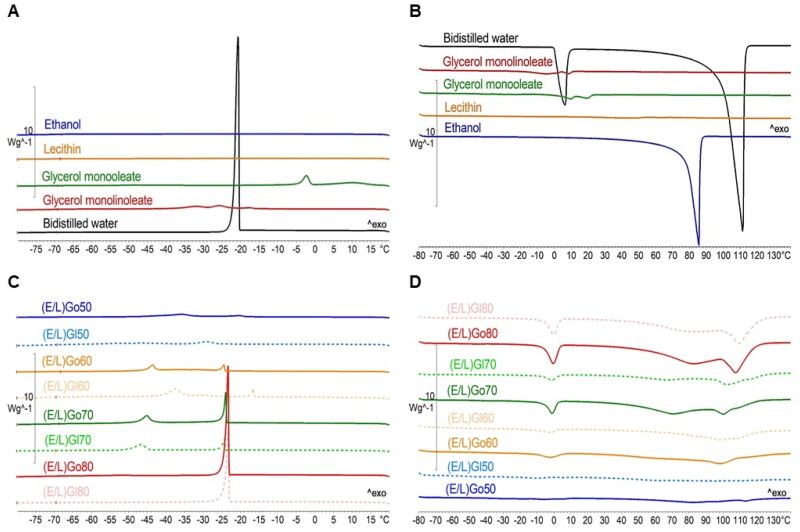
(A) DSC cyrstallization curves and (B) DSC melting curves of individual components plus (C) DSC crystallization curves and (D) DSC melting curves of *in situ* formed gels after reaching equilibrium with water.

Initially, assessment was carried out for individual compounds, i.e. ethanol, lecithin, glycerol monooleate, and glycerol monolinoleate, and bidistilled water. On the crystallization curves of individual components (Figure 6(A)), no thermal events were observed for ethanol or lecithin. However, for glycerol monooleate, a minor broad exothermic peak at T_c1_ = 15.7 °C (ΔH_c1_ = 0.35 J/g) plus a noticeable exothermic peak at T_c2_ = −0.8 °C (ΔH_c2_ = 133.0 J/g) were detected. For glycerol monolinoleate, a small exothermic triple peak appeared (T_c1_ = −16.3 °C, ΔH_c1_ = 1.2 J/g, T_c2_ = −22.9 °C, ΔH_c2_ = 6.7 J/g, T_c3_ = −29.2 °C, ΔH_c3_ = 5.6 J/g). The observed peaks of both lipids can be attributed to the rearrangement and/or crystallization of glycerol monooleate and glycerol monolinoleate molecules, respectively (Chauhan et al., 2016). Linolenic acid (C_18:2_) has one more double bond than oleic acid (C_18:1_), which contributes to its higher degree of unsaturation and greater mobility. This increased mobility is evidenced by more crystallization peaks, as observed in the DSC thermograms (Nyame Mendendy Boussambe et al., 2017). Regarding bidistilled water, at T_c1_ = −20.1 °C (ΔH_c1_ = 239.6 J/g) a sharp exothermic peak appeared, coinciding with the crystallization of supercooled water. Next, on the melting curves of individual components (Figure 6(B)), again no thermal events were observed for lecithin. Nevertheless, in case of ethanol an endothermic peak was detected at T_m1_ = 76.2 °C (ΔH_m1_ = −775.5 J/g) corresponding to its evaporation. In the case of lipid components, their melting was characterized by small double endothermic peaks. More specifically, at T_m1_ = 3.3 °C (ΔH_m1_ = −15.4 J/g) and T_m2_ = 13.7 °C (ΔH_m2_ = −12.6 J/g) for glycerol monooleate, and at T_m1_ = −15.6 °C (ΔH_m1_ = −13.5 J/g) and T_m2_ = 4.9 °C (ΔH_m2_ = −6.9 J/g) for glycerol monolinoleate. The thermal events of bidistilled water were observed at T_m1_ = −0.3 °C (ΔH_m1_ = −278.4 J/g), attributed to ice melting, followed by a broad endothermic peak at T_m2_ = 97.9 °C (ΔH_m2_ = −1709.2 J/g), ascribed to its evaporation.

In the next step, evaluation of *in situ* formed gels after reaching equilibrium with water was performed with special attention given to the water state within them. Water, located near the polar heads of amphiphilic molecules in LCCs, exhibits different thermal properties due to interactions that reduce its degrees of freedom when compared to water that is more distant from the polar heads. Consequently, water molecules forming stronger interactions with amphiphilic molecules solidify at lower temperatures compared to water with weaker interactions, resulting in a lower enthalpy of freezing, sometimes even below the detection limit. Based on this water is classified as non-freezable, freezable interlamellar bound water, and freezable bulk water (Ezrahi et al., 2002).

Figure 6(C) displays the crystallization curves and Table 2 shows the crystallization enthalpies of *in situ* formed gels after equilibrium with water was reached. As regards the crystallization curves of (E/L)Go50 and (E/L)Gl50, two wide exothermic peaks in the range of T_c_ = −18.5 °C to T_c_ = −43.1 °C with small areas under the curve appeared. It seems plausible that herein most of the water was located around the polar headgroups of ethanol and lecithin, with almost no free water in the *in situ* formed gel. Further, in the case of (E/L)Go60, (E/L)Gl60, (E/L)Go70, and (E/L)Gl70, we detected two exothermic peaks between in the range of T_c_ = −16.2 °C to T_c_ = −44.7 °C, representing the crystallization of free water and bound water from the second hydration layer. In these *in situ* formed gels, water was present around the polar headgroups of ethanol and lecithin in addition to free water within the water channels, indicating that the polar headgroups were already saturated with water molecules. Further, it should be emphasized that certain differences were observed among (E/L)Go70 and the other listed *in situ* formed gels. Namely, area under the first exothermic peak at approximately −20 °C, attributed to free water within the system, was 3- to 5-times larger in the case of (E/L)Go70, which corresponds well with the results of water uptake evaluation and is also shown in the *in vitro* release testing presented later in the study. In regard to the crystallization curves of (E/L)Go80 and (E/L)Gl80, only one exothermic peak appeared at −22.6 °C and 22.8 °C, respectively, which in terms of the size of the area under the curve and shape, most closely resembles the reference peak of bidistilled water. It can be postulated that the polar headgroups of ethanol and lecithin are already fully saturated with water molecules of the first and the second hydration layer and that a significant amount of absorbed water is in the form of free water within the water channels. It seems plausible that this free water mostly belongs to lamellar mesophases, which were detected in addition to hexagonal LCCs by PLM analysis of these *in situ* formed gels. To note, all of these findings are in good agreement with results of gelation test and water uptake evaluation, which confirm the lowest water absorption of (E/L)Go50 and (E/L)Gl50, contrary to (E/L)Go80 and (E/L)Gl80 with the highest water uptake.

Figure 6(D) shows the melting curves, and Table 3 presents the melting enthalpies of *in situ* formed gels after equilibrium with water was reached. Melting of ice formed within the cooling cycle of the analysis was noted at approximately 0 °C. In addition, evaporation of ethanol was detected at approximately 78 °C, while water evaporated at approximately 100 °C. The obtained melting curves confirm trends observed from the crystallization curves, where the positions and areas under the curves were directly proportional to the content of absorbed water within *in situ* formed gels.

**Table 3.** DSC thermodynamic parameters (i.e. melting temperature and melting enthalpy) of the heating cycle of *in situ* formed gels after reaching equilibrium with water.

	T_m1_ (°C)	ΔH_m1_ (J/g)	T_m2_ (°C)	ΔH_m2_ (J/g)	T_m3_ (°C)	ΔH_m3_ (J/g)
(E/L)Go50	–	–	–	–	–	–
(E/L)Gl50	–	–	–	–	–	–
(E/L)Go60	–10.8	–30.6	–	–	87.2	–53.1
(E/L)Gl60	–9.8	–28.4	–	–	89.3	–74.7
(E/L)Go70	–5.5	–56.5	55.8	–54.8	93.4	–87.3
(E/L)Gl70	–7.4	–29.3	58.0	–10.0	95.7	–78.5
(E/L)Go80	–5.8	–104.6	62.9	–138.6	99.5	–173.7
(E/L)Gl80	–5.7	–98.9	59.1	–81.4	102.2	–158.7

### Rheological measurements

Microstructure evaluation of precursor formulations as well as *in situ* formed gels after reaching equilibrium with water was further upgraded by rheological tests, which provided additional insights into their flow behavior after applied stress as well as viscoelastic characteristics. These measurements contributed to a comprehensive understanding of the structural changes going along with sol–gel transition in addition to structural analyses performed using PLM and DSC.

Firstly, rotational measurements (Table 4) were performed to elucidate the flow behavior of a system subjected to applied stress, offering an insight into microstructural alterations upon SC administration. The viscosity curves of all precursor formulations obtained at 25 °C demonstrated a constant viscosity regardless of increasing shear rate. This finding confirm that all precursor formulations exhibited Newtonian fluid behavior, a desirable feature for injectables designed for SC administration. When comparing the viscosities of precursor formulations at the lowest shear rate, a positive correlation between lipid content and viscosity was revealed. However, it is important to note that viscosities of all precursor formulations ranged from 17.0 cP to 36.9 cP, being far below 50 cP, therefore confirming their suitability for SC injection (Miller et al., 2010). Precursor formulations with glycerol monolinoleate as a lipid phase generally exhibited lower viscosities. Further, rotational tests were also performed for *in situ* formed gels after reaching equilibrium with water at a temperature of 37 °C. Their viscosities decreased with increasing shear rate until they reached a constant value at high shear rates, hence all *in situ* formed gels can be classified as non-Newtonian pseudoplastic systems, which is also consistent with our expectations. Upon evaluating the viscosities of *in situ* formed gels, the viscosity values of *in situ* formed gels were prominently higher when compared to precursor formulations. In addition, if negligible variations in viscosity values at the lowest shear rate (1 s^−1^) for precursor formulations were detected, notable variations were observed for *in situ* formed gels which can be explained with their spontaneous formation. Therefore, viscosities for *in situ* formed gels are presented at 2 s^−1^ with similar trend to that observed for their respective precursor formulations. More specifically, viscosity values ranged from 698.0 Pa·s to 111.9 Pa·s. *In situ* formed gels containing glycerol monolinoleate as a lipid phase in general exhibited lower viscosities. All of these results correlate well with gelation test and the corresponding PLM analysis.

**Table 4.** Viscosities (mean ± SD (*n* = 2)) of precursor formulations determined at the shear rate of 1 s^−1^ and temperature of 25 °C and *in situ* formed gels after reaching equilibrium with water determined at the shear rate of 2 s^−1^ and temperature of 37 °C.

	Precursor formulation η (cP)	*In situ* formed gel η (Pa·s)
(E/L)Go50	36.9 ± 0.0	689.0 ± 20.0
(E/L)Gl50	29.3 ± 0.0	429.3 ± 14.1
(E/L)Go60	28.8 ± 0.0	470.5 ± 13.4
(E/L)Gl60	23.2 ± 0.0	323.2 ± 11.2
(E/L)Go70	24.1 ± 0.0	420.0 ± 15.8
(E/L)Gl70	18.5 ± 0.0	205.5 ± 5.9
(E/L)Go80	19.8 ± 0.0	113.5 ± 7.0
(E/L)Gl80	17.0 ± 0.0	111.9 ± 4.1

**Figure 7.**
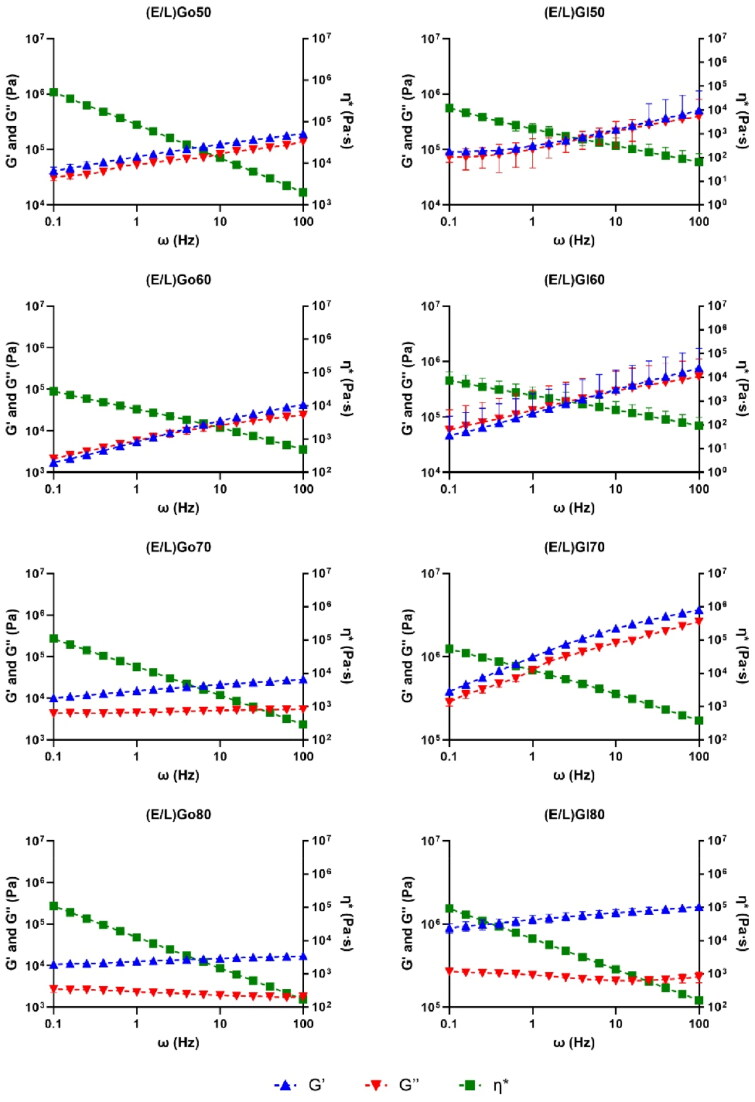
Storage modulus (G’), loss modulus (G’’), and complex viscosity (η*) as function of angular frequency (ω) at a stress of 0.1 % determined for *in situ* formed gels after reaching equilibrium with water at 37 °C. Data are expressed as mean ± SD (*n* = 2).

Further, oscillatory shear frequency sweep measurements were performed for *in situ* formed gels after reaching equilibrium with water at 37 °C (Figure 7), as they provide important information regarding the viscoelastic properties of a system corresponding to its network structure. Therefore, rheological parameters, including storage (G′) and loss (G″) moduli, as well as complex viscosity (η*), across various angular frequencies were recorded. The G′ modulus reflects elastic properties of a system, with high values demonstrating a system with strong elasticity and structure, while high G’’ values suggest a predominantly viscous, liquid-like material. Depending on the dominant modulus, a system can be classified as either elastic or viscous. The G’ modulus was generally higher than the G″ modulus with increasing frequency, whereas complex viscosity was decreasing with increasing frequency in case of all *in situ* formed gels, indicating predominantly elastic behavior. This is characteristic of gel-like systems and can be attributed to the well-organized microstructure of the LCCs. More specifically, in the case of (E/L)Go50, (E/L)Gl50, (E/L)Go60, (E/L)Gl60, and (E/L)Gl70, the G′ and G″ moduli were strongly enhanced with increasing frequency. This observed rheological pattern is representative of hexagonal LCCs (Xingqi et al., 2019) and is good agreement with PLM photomicrographs. Furthermore, similar curves were also observed for (E/L)Go70, whereby the G′ and G″ moduli were less enhanced with increasing frequency, indicating a less dense network of hexagonal LCCs. To note, less strong interactions between surfactant molecules and water were also confirmed by DSC measurements for this *in situ* formed gel. Next, the rheological behavior of (E/L)Go80 and (E/L)Gl80 also correlated well with the results of other assessments. Namely, in the case of these two *in situ* formed gels, both the G′ and G″ moduli were nearly independent of the angular frequency over the entire range investigated with the large gap between the both curves, suggesting the coexistence of hexagonal mesophases along with lamellar LCCs (Mistry et al., 2023), as we had also anticipated based on PLM analysis.

### In vitro release testing

#### Selection of the release medium

The newly developed *in situ* forming liquid crystalline systems were designed for the sustained release of the peptide drug Tα1 with inherent poor stability. Therefore, to ensure an optimal *in vitro* release testing experiment for the period of 2 weeks, preliminary studies were conducted to assess the influence of the release medium on the Tα1’s stability. In addition, given that the experiment was carried out at 37 °C and that the samples were stored for subsequent UHPLC analysis after sampling, the effect of storage temperature on the Tα1’s stability was also evaluated (Table 5).

**Table 5.** Percentage of undegraded Tα1 (mean ± SD (*n* = 3)) in different media and at different temperatures determined after 2 weeks within the selection of the release medium studies.

	T = −20 °C	T = 8 °C	T = 25 °C	T = 37 °C
Ultrapure water	99.5 ± 0.5	100.0 ± 0.3	<0.5	<0.5
Phosphate buffer solution (pH = 7.4)	114.0 ± 1.1	31.5 ± 0.6	<0.5	<0.5
Phosphate buffer solution (pH = 6.8)	99.3 ± 2.4	99.7 ± 0.4	<0.5	<0.5
Simulated body fluid (pH = 7.4)	106.2 ± 1.8	86.0 ± 1.5	<0.5	<0.5
Phosphate buffer solution (pH = 6.8) with 5% of ethanol	98.7 ± 0.0	99.1 ± 0.2	96.1 ± 0.2	93.7 ± 0.0
Ethanol	99.6 ± 0.7	100.2 ± 0.1	83.0 ± 0.4	71.8 ± 2.3

Proportion of ethanol (96%) is given in m/m %.

Within the assessment of release media, we examined the Tα1’s stability regarding the absence (ultrapure water) or the presence of ions in various buffers (PBS, simulated body fluid), the pH value (6.8 and 7.4) and the proportion of ethanol (5%, 100% (m/m)). Additionally, as part of the temperature stability testing, the Tα1’s stability was evaluated at the following temperatures for all release media: −20 °C (freezer temperature), 8 °C (refrigerator temperature), 25 °C (room temperature), and 37 °C (body temperature). At −20 °C and 8 °C, the Tα1’s stability was adequate within all tested combinations of release media. However, evident differences in stability were observed at elevated temperatures. To note, we found that by adding a small proportion of ethanol improved stability of the peptide drug Tα1 in the release medium was obtained, proving its key influence on Tα1’s stability. Considering the observed finding and the literature data reporting that a slightly acidic pH improves Tα1’s stability (Dai et al., 2012), PBS (pH = 6.8) containing 5% (m/m) of ethanol was selected as the most appropriate release medium at all tested temperatures over the entire testing period.

The potential effect of ethanol on *in situ* depot formation was investigated by PLM microstructural examination of the *in situ* formed gels exposed to the release medium containing 5% (m/m) ethanol after equilibrium with water was reached (data not shown). It has been demonstrated that this proportion of ethanol had no effect on depot formation.

#### In vitro release of the Tα1 from in situ formed gels

**Figure 8.**
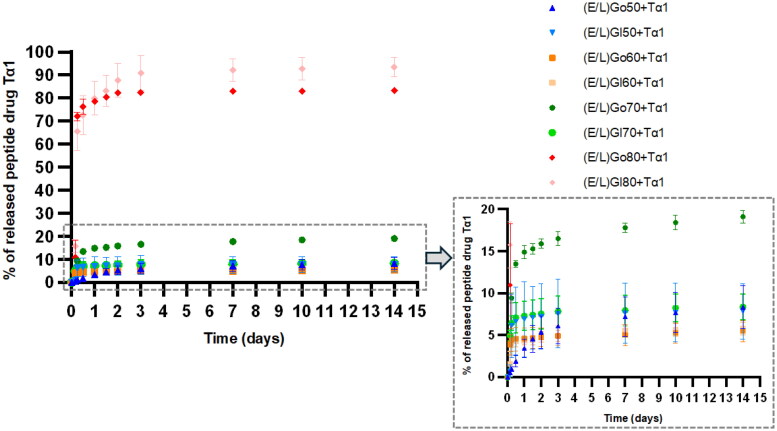
*In vitro* release profiles of the peptide drug Tα1 from *in situ* formed gels. Data are expressed as mean ± SD (*n* = 4).

Achieving the sustained release of the peptide drug Tα1 was one of the pivotal aspects we focused on in the development of the *in situ* forming liquid crystalline systems in this study. Thus*, in vitro* release testing was performed to evaluate their potential for minimization of the Tα1’s dosing frequency that could greatly improve patient compliance upon clinical translation of the systems. Figure 8 displays the cumulative release of the peptide drug Tα1 *in vitro* from the *in situ* formed gels over a period of 2 weeks.

All the studied *in situ* formed gels demonstrated the sustained release profiles; however, noticeable differences were observed among them. (E/L)Go80 and (E/L)Gl80 exhibited the greatest total drug release after 2 weeks with 84.2% and 93.4%, respectively. Further, (E/L)Go70 demonstrated 19.1% of released Tα1 after 2 weeks. It is important to note that this represented 2- to 4-times greater total drug release when compared to other *in situ* formed gels. Namely, they exhibited comparable amount of released drug after 2 weeks, being 8.4% for (E/L)Gl70, 8.3% for (E/L)Go50, 7.8% for (E/L)Gl50, 5.8% for (E/L)Gl60 and 5.5% (E/L)Go60.

The observed differences can be explained by bidirectional relationship among variables influencing the drug release mechanism from LCCs. Specifically, the hydrophilic characteristics of the peptide drug Tα1 (Goldstein et al., 1977), which determine the affinity for the water channels of LCCs, as well as the composition and the microstructure of the LCCs with the interrelated water uptake capacity. It is known from the literature that the release of hydrophilic drugs from lamellar LCCs, which are in general more highly hydrated mesophases, is more rapid than from hexagonal LCCs with relatively low water absorption. This phenomenon can be attributed to an increase in the water channels available for release of hydrophilic drugs with increasing water content within the system (Borgheti-Cardoso et al., 2015; Elnaggar et al., 2016).

In the present study, the coexistence of hexagonal mesophases along with lamellar LCCs was confirmed by PLM analysis and oscillatory measurements for (E/L)Go80 and (E/L)Gl80. Consequently, the water uptake capacity of (E/L)Go80 and (E/L)Gl80 was exceptionally high and the release was greater than that of the other gels formed *in situ*. In keeping with this, their release profiles were also consistent with the explanation provided above. Namely, the other *in situ* formed gels formed only hexagonal mesophases, resulting in their noticeably sustained release profiles. Among these, (E/L)Go70 demonstrated a moderately greater total drug release, which corresponded with its higher water uptake capacity and the associated larger proportion of free water, as confirmed by DSC measurements as well. In other words, larger amount of free water within water channels of hexagonal mesophases present in (E/L)Go70 enabled moderately greater release of the hydrophilic peptide drug Tα1. However, it is still necessary to take into account that (E/L)Go70 formed only hexagonal mesophases and that water channels within them are closed to the external environment, hence water diffusion is retarded (Chavda et al., 2022). Other *in situ* formed gels exhibiting solely hexagonal mesophases showed similar water uptake capacities and similar intermolecular network, as identified by DSC analysis, their amount of the released peptide drug Tα1 was comparable.

Further, the peptide drug Tα1’s secondary structure using CD spectroscopy was examined. Considering the literature indicating that Tα1 is an intrinsically disordered peptide at neutral pH and body temperature in water, with various solvents capable of inducing structural changes (Hoch & Volk, 2016), its structural stability was systematically evaluated in different samples throughout processing. Supplementary Figure S3A shows the dichroic profile of the peptide drug Tα1 in ethanol for incorporation into formulation, indicating β-sheet conformation (Greenfield, 2006). Further, the CD spectrum of the peptide drug Tα1 in the release medium post-drug release testing, shown in Supplementary Figure S3B, indicates that the peptide adopted a random coil conformation in the aqueous environment, which aligns well with findings from (Grottesi et al., 1998). In addition, it also correlates with the CD spectra obtained for the dissolved lyophilisate of the peptide drug Tα1 in the release medium and in PBS itself (Supplementary Figure S3C and S3D).

Taken together, these results confirm that the peptide drug Tα1 adopts and maintains its native conformation, characteristic of aqueous environment, in the release medium after the completion of the *in vitro* release testing. Notably, the conformational changes in different environments may serve as structural prerequisites for Tα1’s interaction with lymphocyte membranes, potentially representing the initial event in lymphocyte activation during immune response modulation, thereby highlighting the functional relevance (Grottesi et al., 1998).

To conclude, the results of the *in vitro* release testing demonstrated that adjusting the composition of precursor formulations facilitates the regulation of *in situ* formed gels’ microstructure, thereby controlling the release profiles of the incorporated peptide drug Tα1. Furthermore, the release profiles obtained over a period of 2 weeks imply the potential of the *in situ* formed gels innovated in this study to prolong the peptide drug Tα1’s release and notably minimize its dosing frequency. Nevertheless, it is important to note that the % of the released peptide drug Tα1 increases only slightly after initial release observed in the first days of the *in vitro* release testing. A similar release behavior has also been reported for the peptide drug leuprolide acetate from liquid crystalline hexagonal mesophases (Báez-Santos et al., 2016). Upon administration, the SC tissue pressure along with flow of the SC interstitial fluid perfusing the *in situ* formed depots is expected to assist the erosion of the *in situ* formed gel matrix and enhance the drug release, though (Torres-Terán et al., 2023).

## Conclusion

This study has shown that lyotropic liquid crystals represent a flexible and versatile platform that enables the regulation of macro and microstructure, and thereby the release profile and overall performance, through minimal adjustments in the component ratios. We report here the development of an *in situ* forming system for SC administration for potential sustained release of the peptide drug Tα1. Through a systematic design, we accomplished all the main objectives that we set at the beginning of the study. Firstly, nonaqueous precursor formulations demonstrated optimal rheological properties for SC injection. Further, an easy and quick *in situ* phase transition of precursor formulations to hexagonal LCCs was obtained. The change was triggered by water absorption, which represents the least invasive stimulus for phase transition occurrence. Finally, the obtained release kinetics of the peptide drug Tα1 from *in situ* formed gels imply a prolonged release behavior that could notably minimize its dosing frequency. These results highlight the great potential of the newly developed *in situ* forming liquid crystalline systems as injectable long-acting depots for SC administration of the peptide drug Tα1, promoting patient adherence.

## Supplementary Material

Supplementary_material.docx

## Data Availability

The data presented in this study are available in article and supplementary materials.
