# Supplementary material for: Exploiting the potential of in situ forming liquid crystals: development and in vitro performance of long-acting depots for peptide drug thymosin alpha 1 subcutaneous administration
Source: Drug Deliv. 2025 Mar 11;32(1):2460708. doi: 10.1080/10717544.2025.2460708 (PMC11899226; doi:10.1080/10717544.2025.2460708)
Supplement: Supplementary_material.docx [file IDRD_A_2460708_SM4866.docx]

**Supplementary Figure S1** Morphology of *in situ* formed gels from precursor formulations upon contact with excess aqueous medium within the scope of gelation test at selected predetermined time points.

**Supplementary Figure S2** Representative PLM photomicrographs of *in situ* formed gels from precursor formulations upon contact with excess aqueous medium obtained through phase transition analysis within gelation test at selected predetermined time points at 37 °C.

**Supplementary Figure S3** CD spectra of (A) the peptide drug Tα1 in ethanol for incorporation into formulation, (B) the peptide drug Tα1 in the release medium post-drug release testing, (C) the dissolved lyophilisate of the peptide drug Tα1 in the release medium, and (D) the dissolved lyophilisate of the peptide drug Tα1 in PBS.


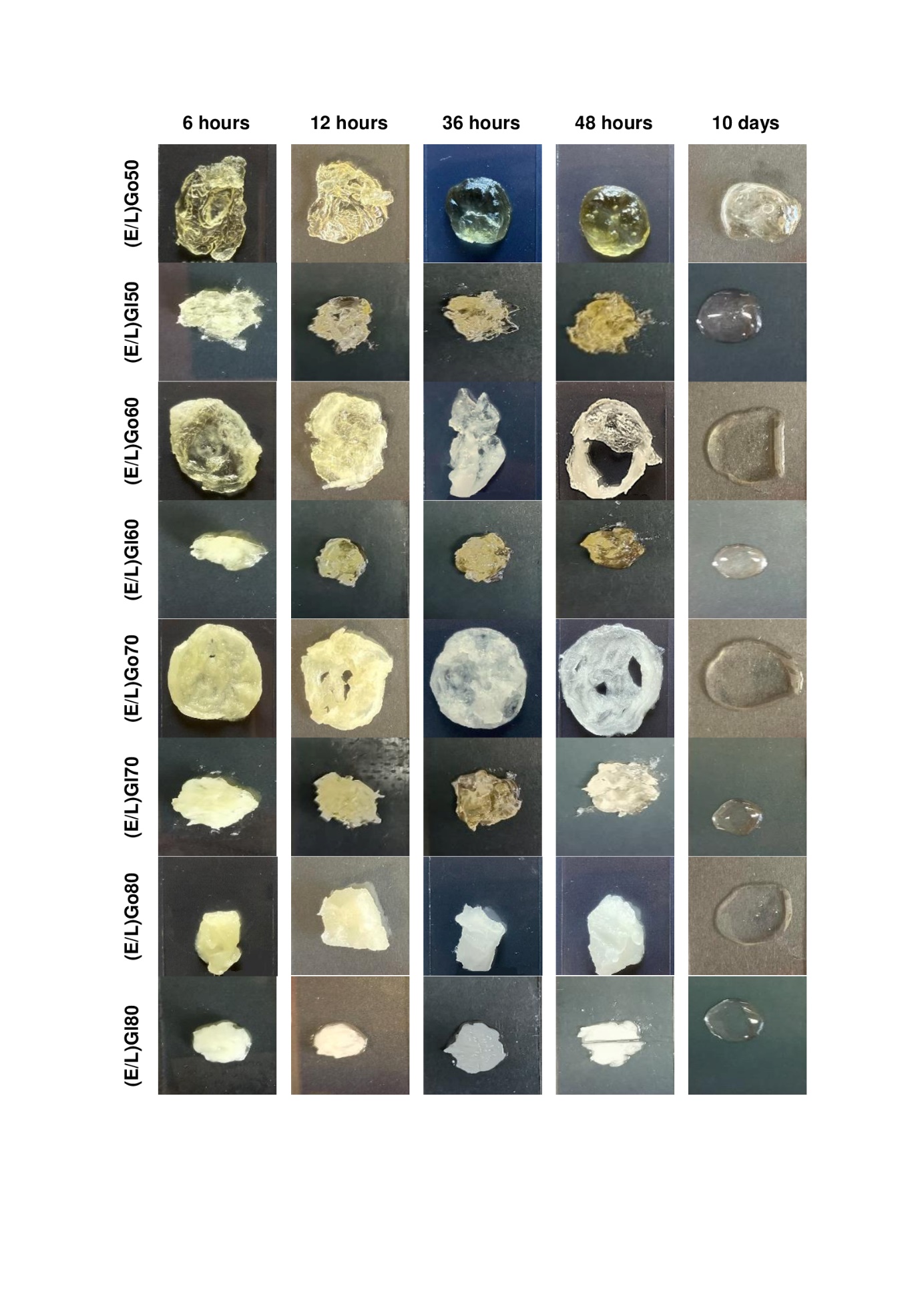


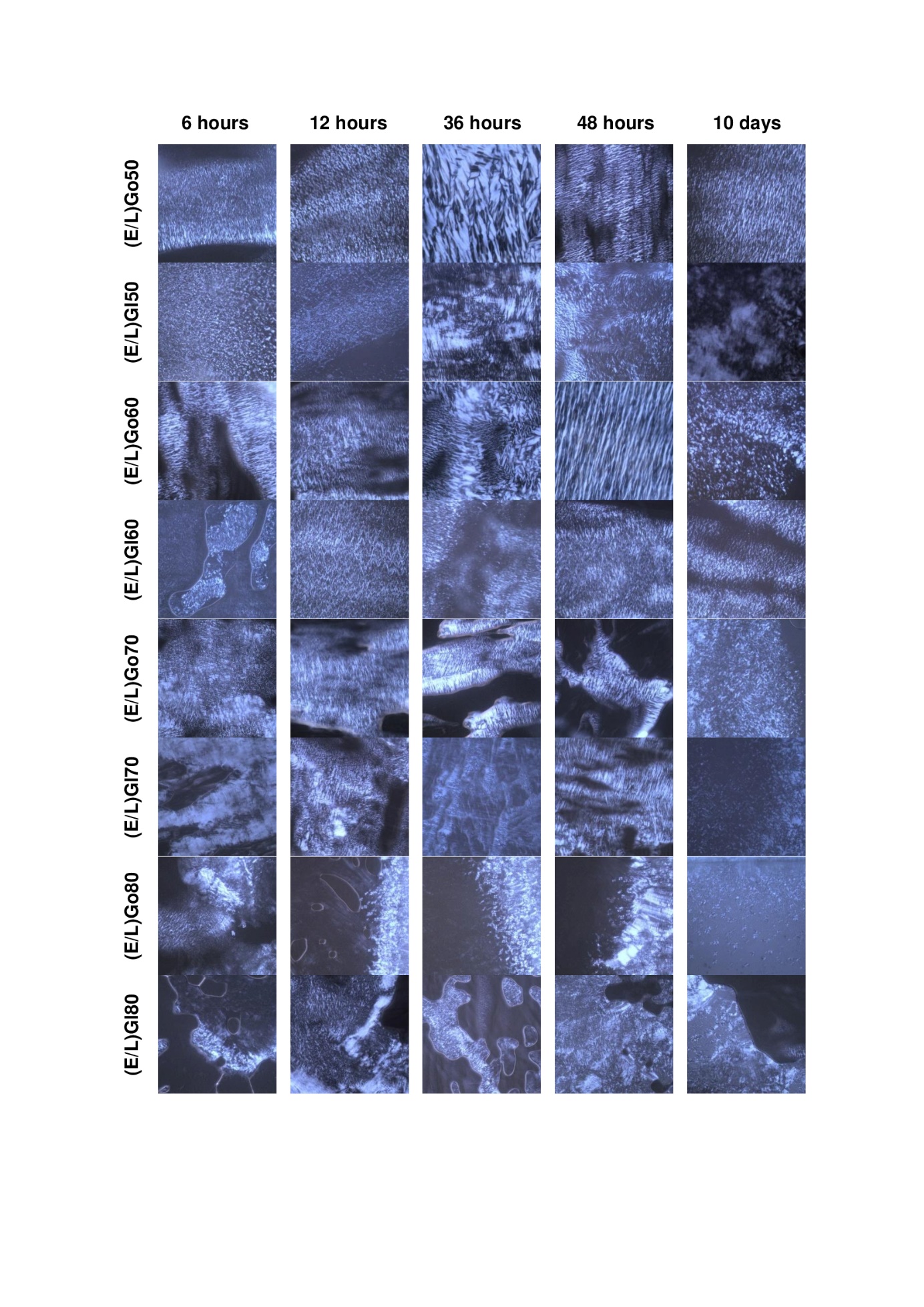


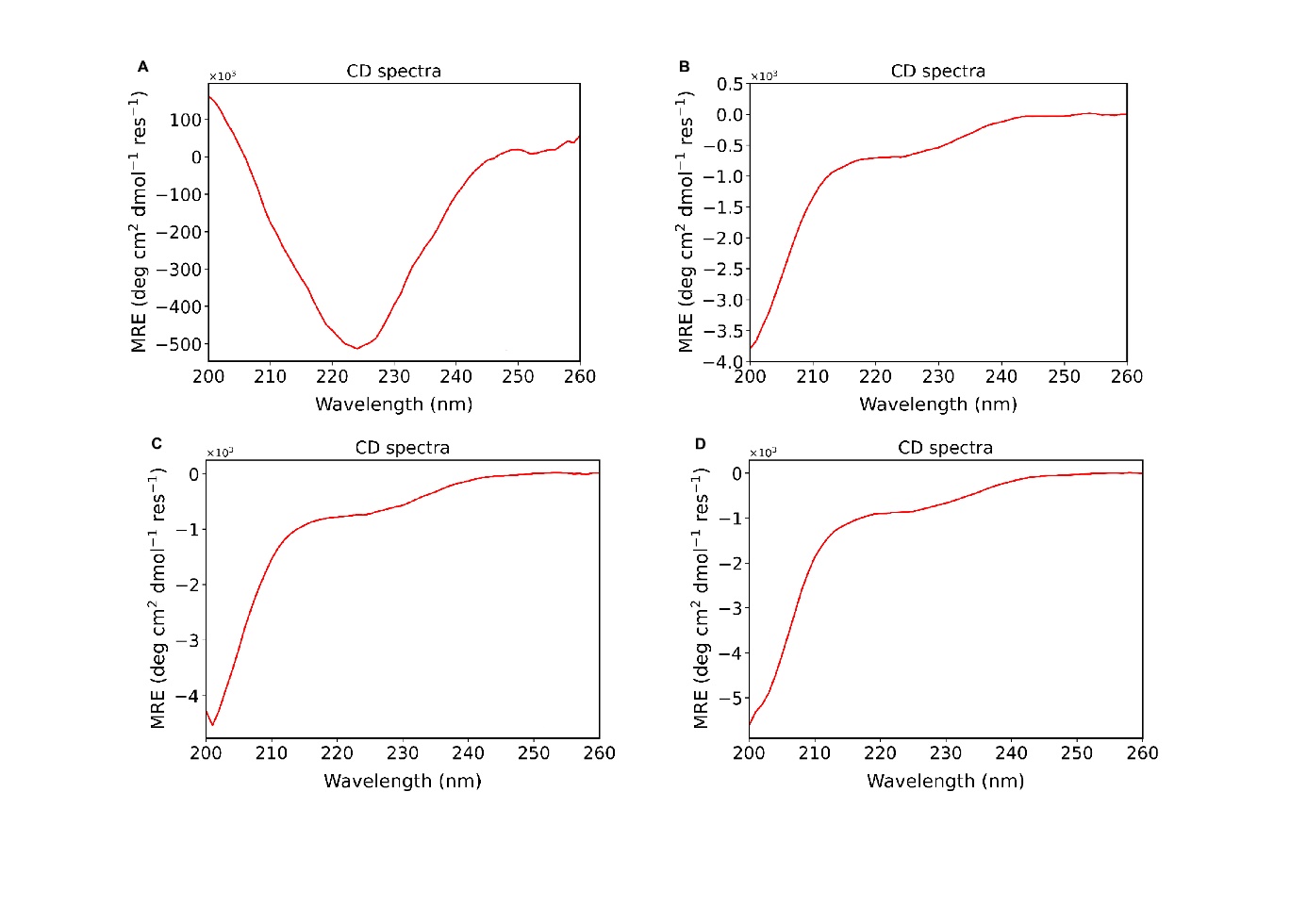


Supplementary Figure S3 added after revision.


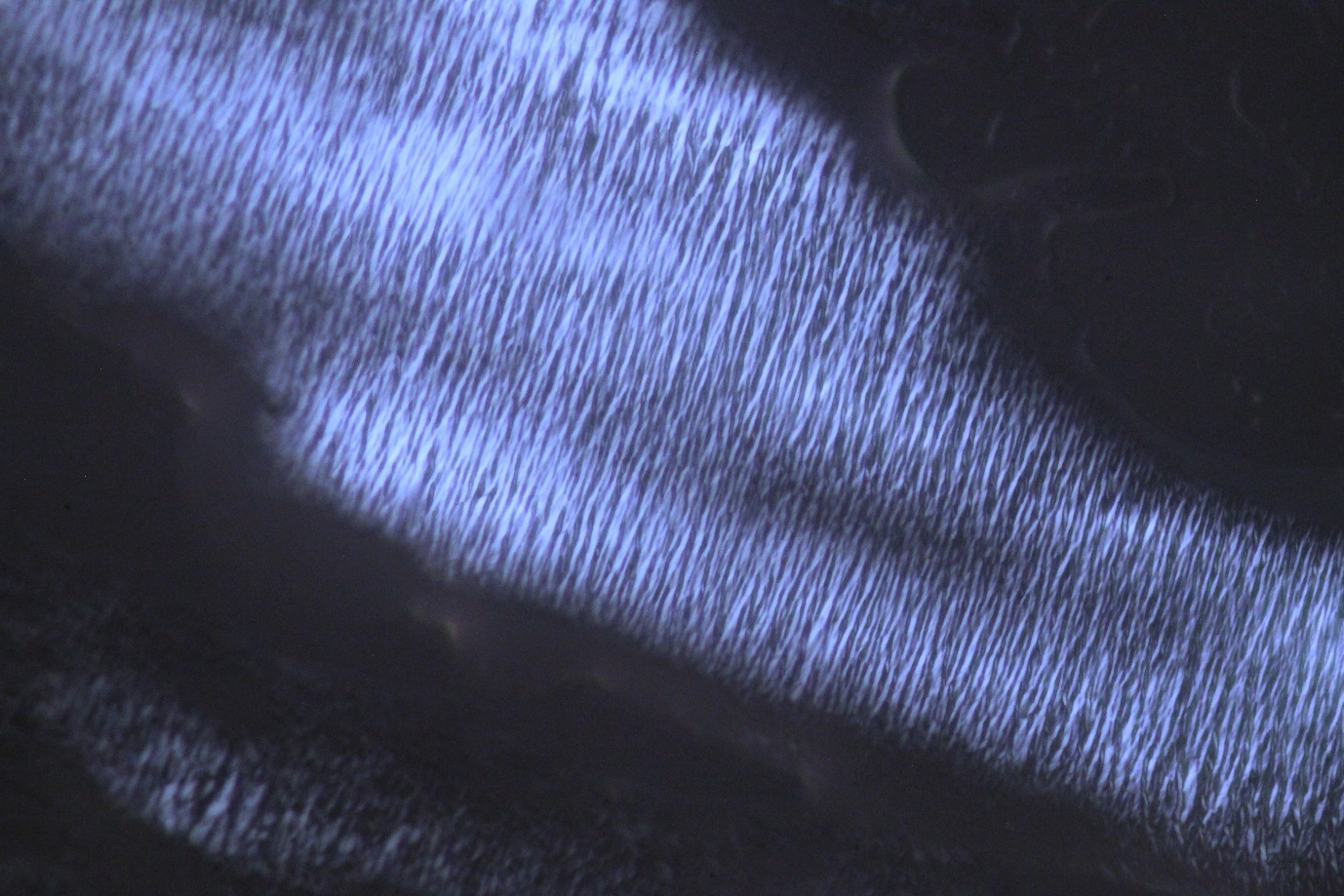


Original Image for Figure 4: (E/L)Go50, 1 hour; left part of the image is included in the figure.


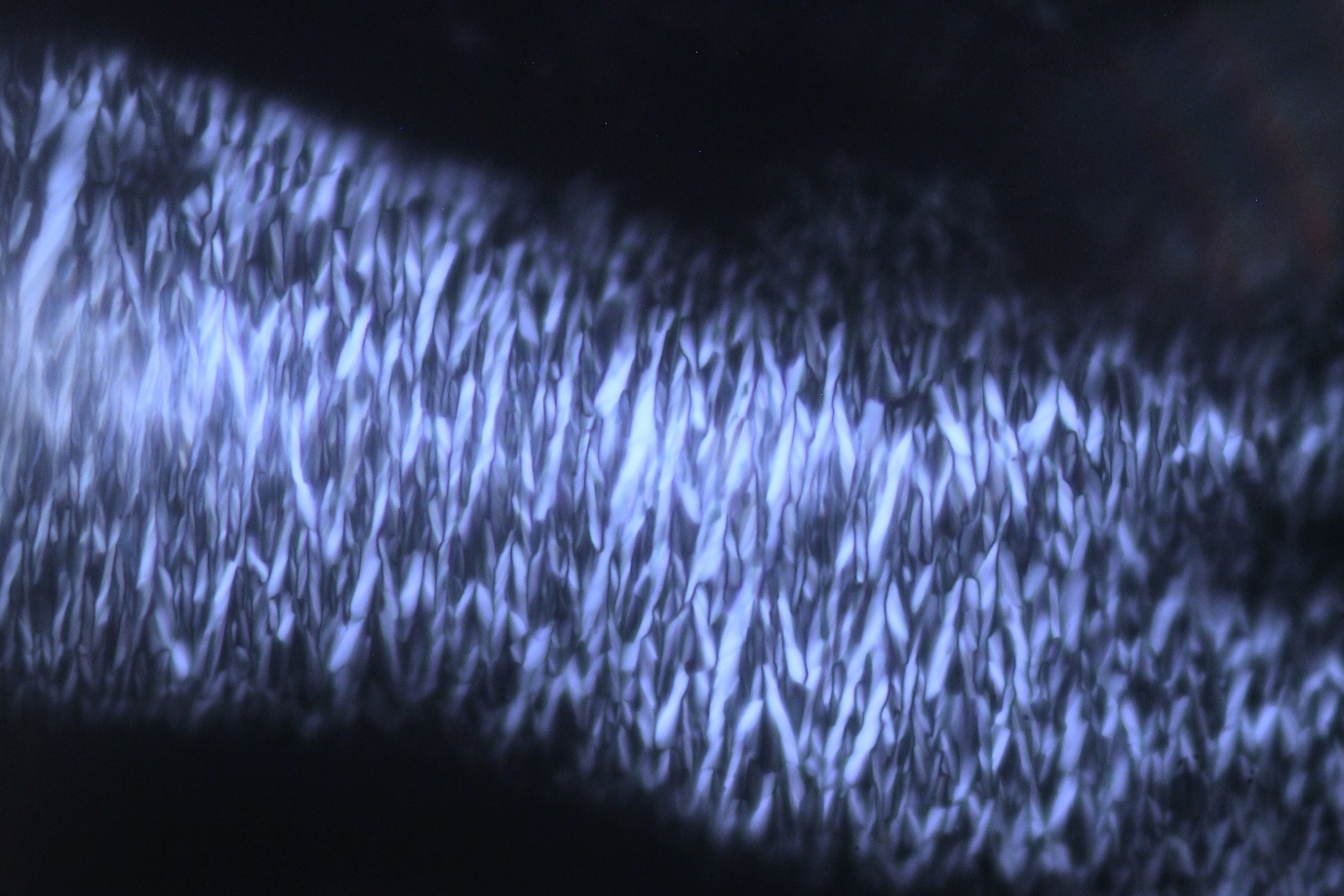


Original Image for Figure 4: (E/L)Go50, 24 hours; middle part of the image is included in the figure.


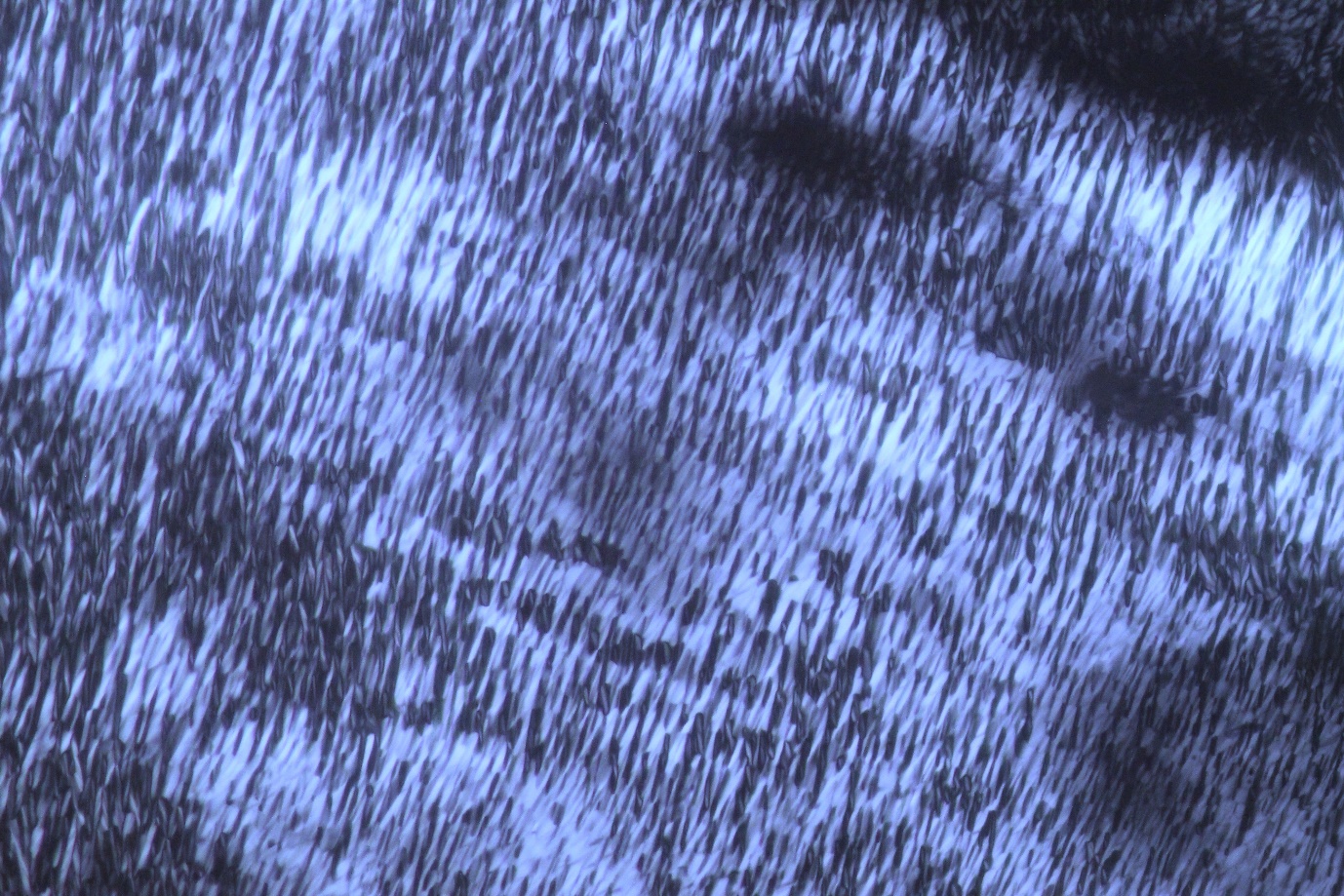


Original Image for Figure 4: (E/L)Go50, 72 hours; middle part of the image is included in the figure.


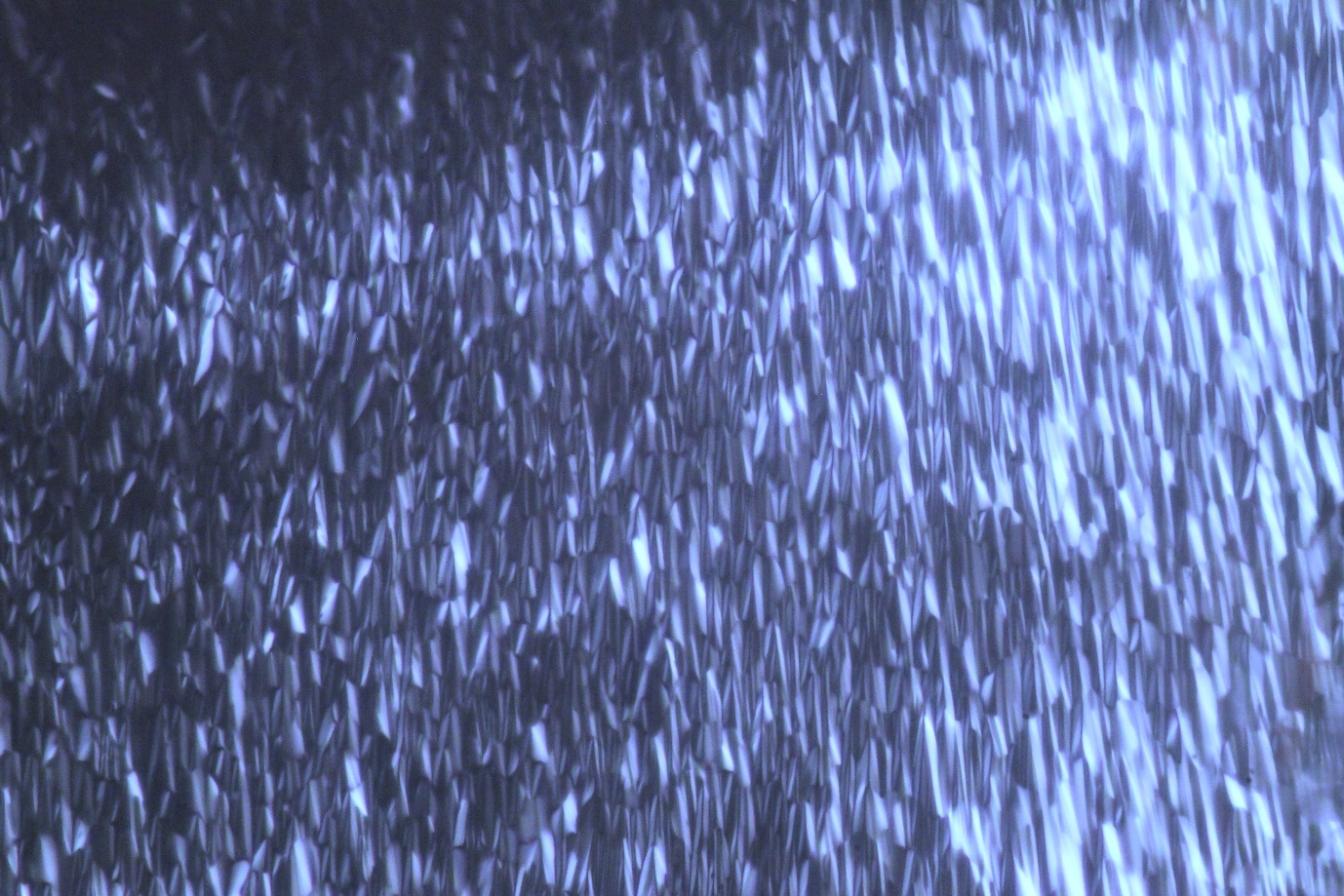


Original Image for Figure 4: (E/L)Go50, 7 days; middle part of the image is included in the figure.


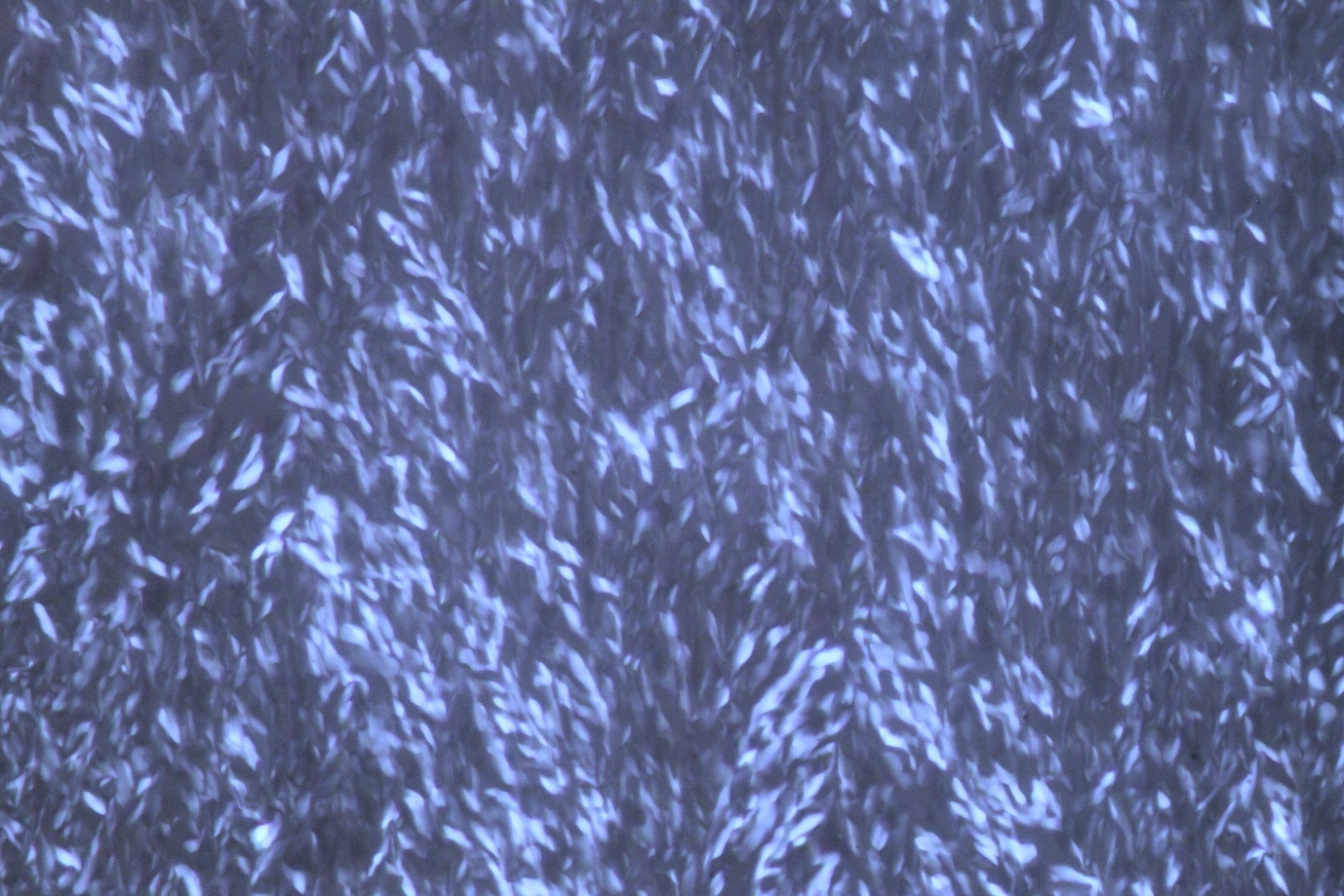


Original Image for Figure 4: (E/L)Go50, 14 days; middle part of the image is included in the figure.


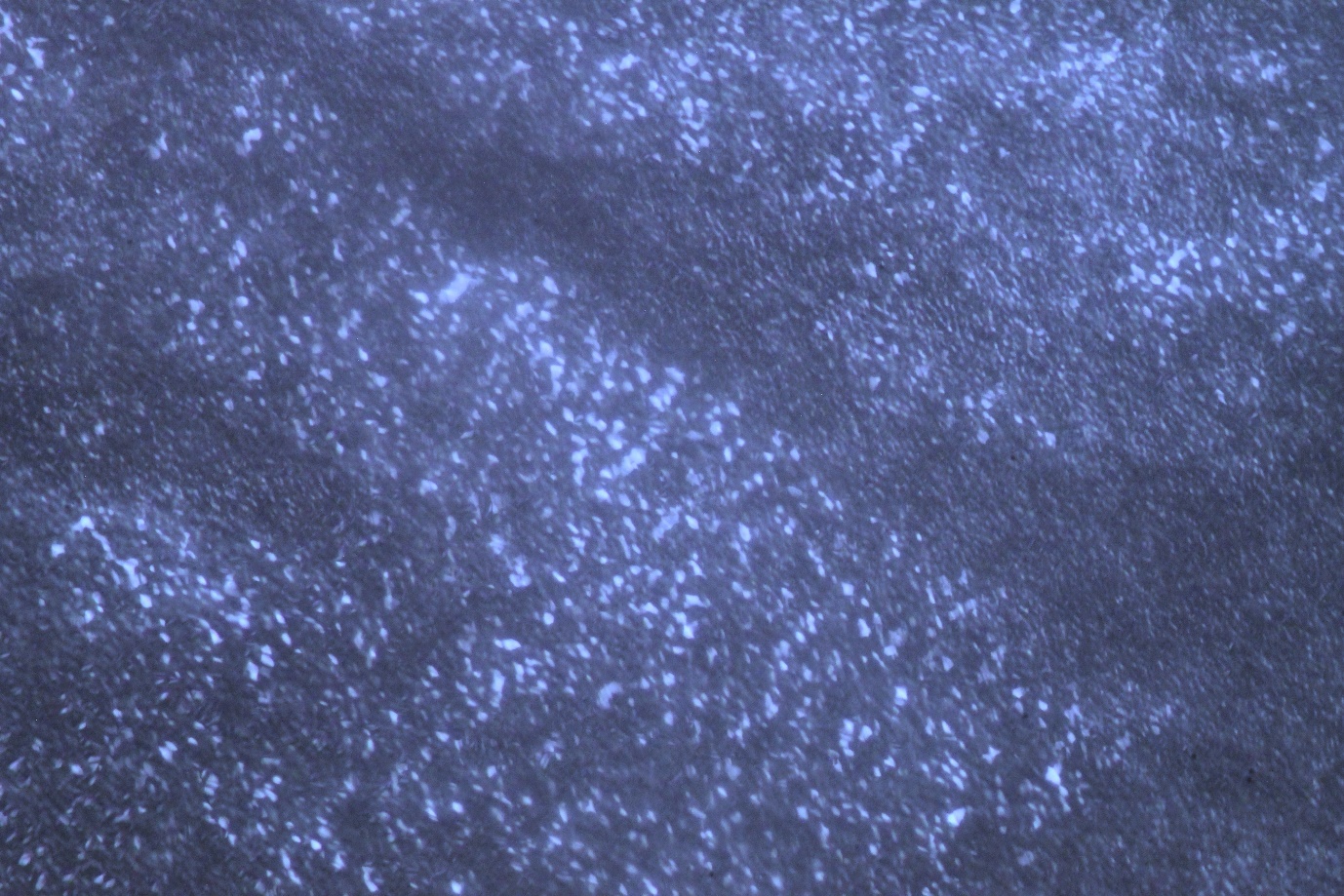


Original Image for Figure 4: (E/L)Gl50, 1 hour; middle part of the image is included in the figure.


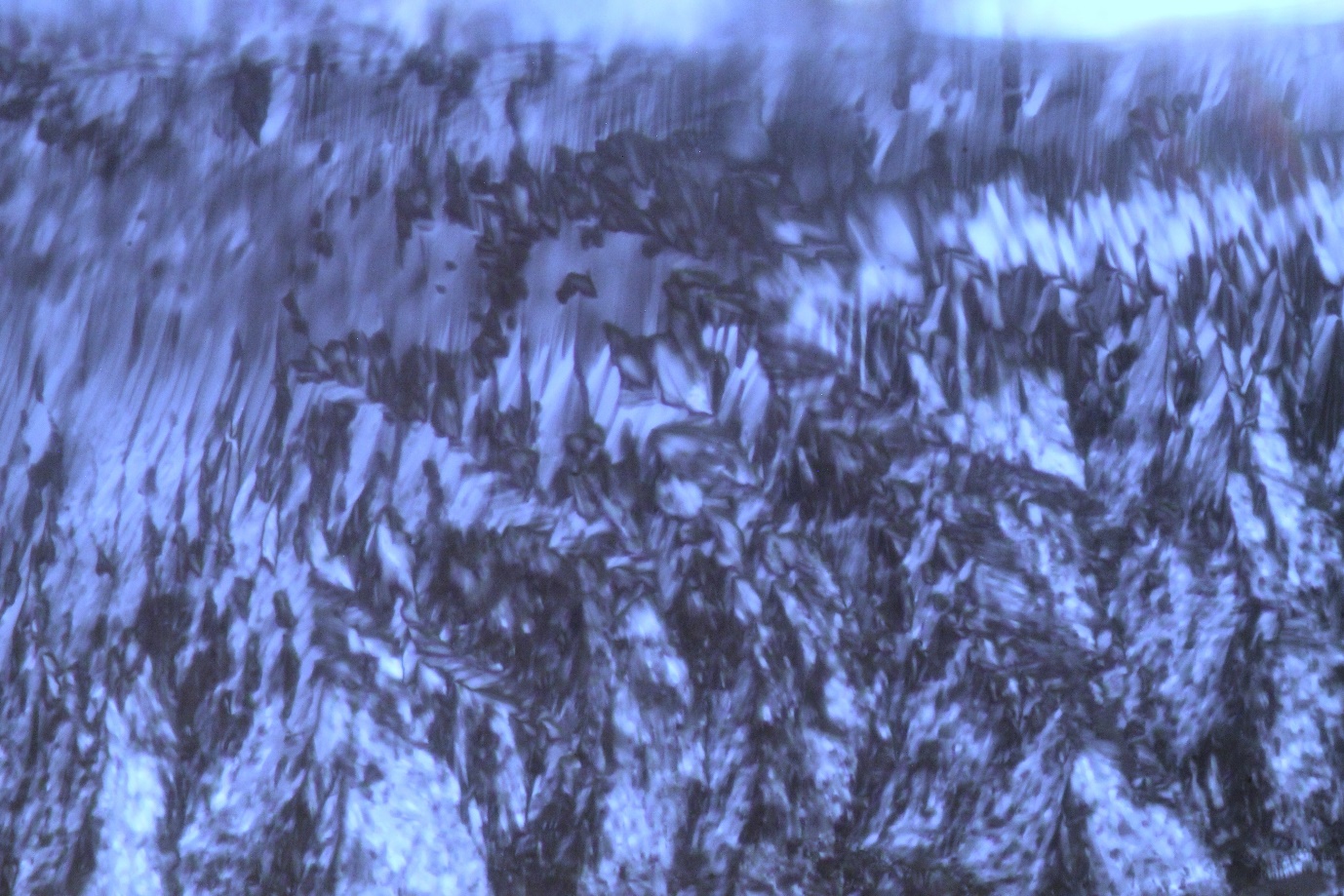


Original Image for Figure 4: (E/L)Gl50, 24 hours; middle part of the image is included in the figure.


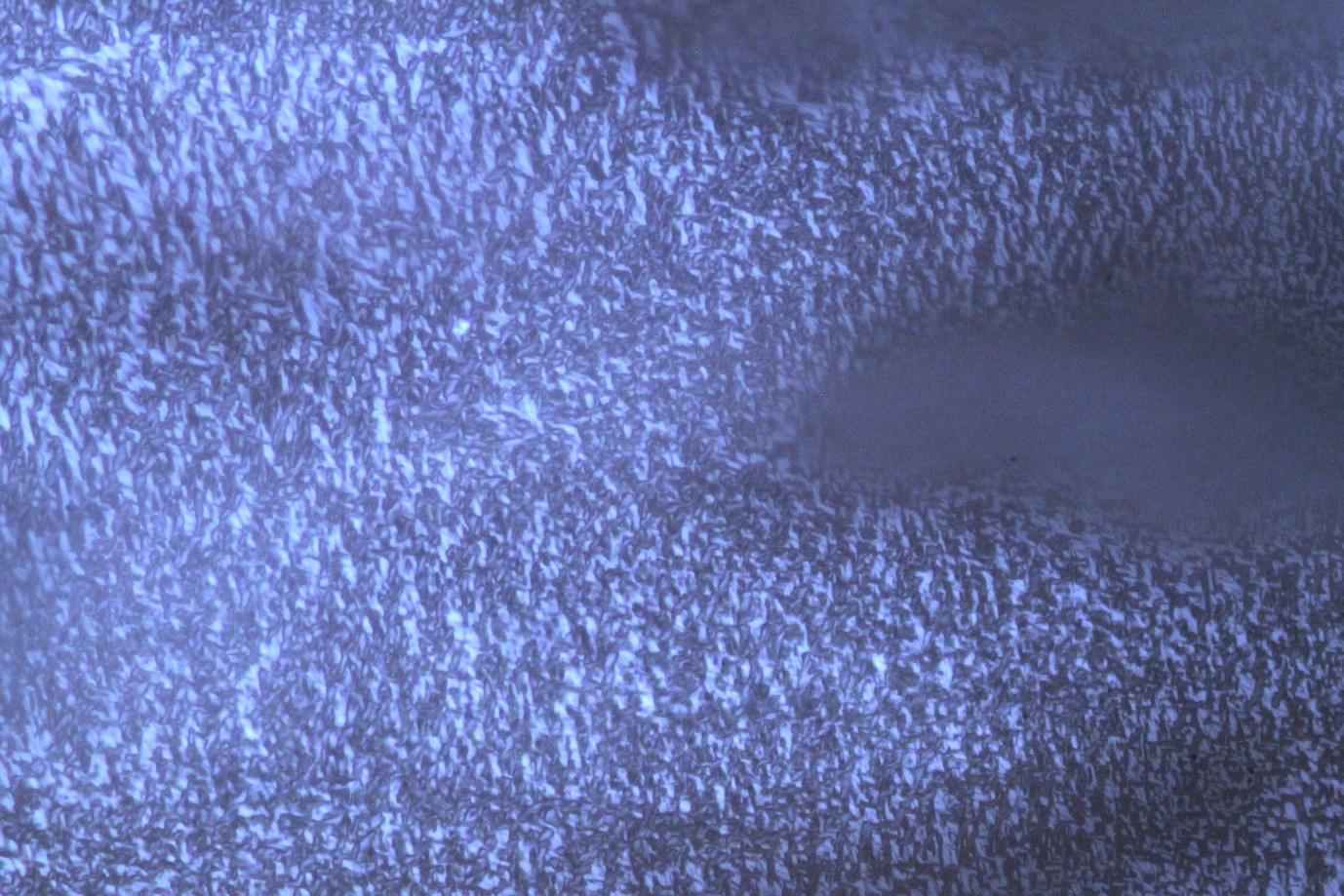


Original Image for Figure 4: (E/L)Gl50, 72 hours; middle part of the image is included in the figure.


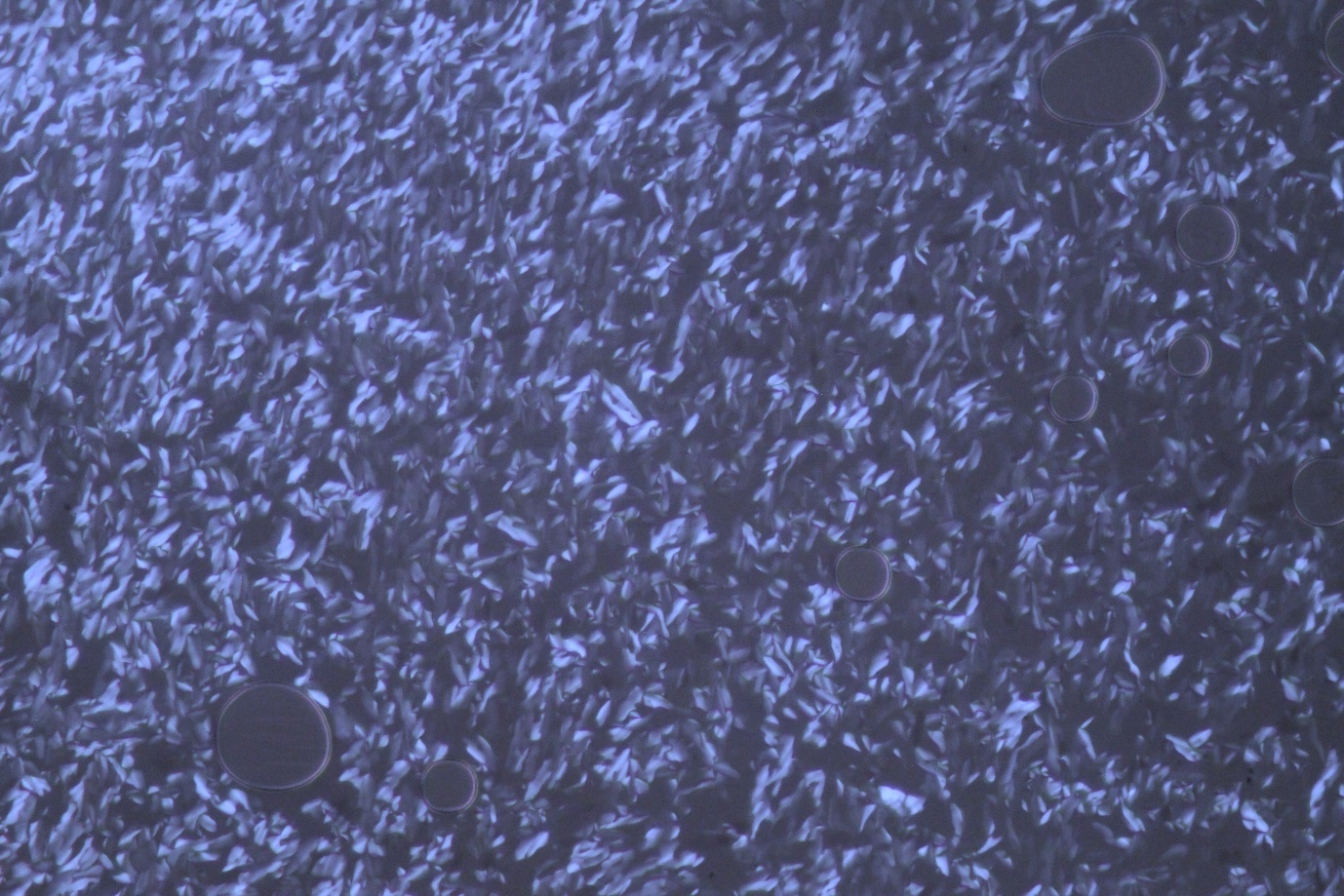


Original Image for Figure 4: (E/L)Gl50, 7 days; middle part of the image is included in the figure.


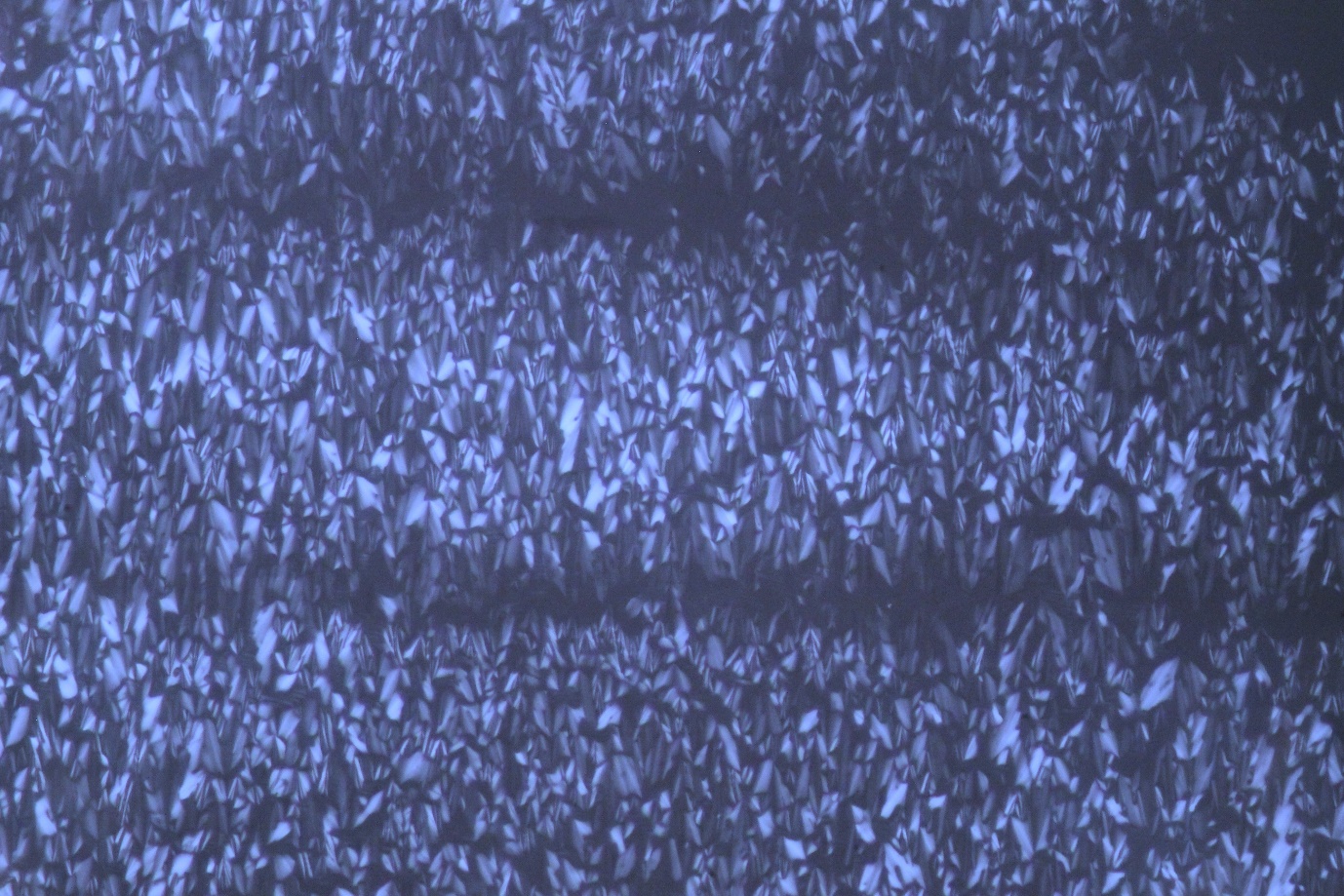


Original Image for Figure 4: (E/L)Gl50, 14 days; middle part of the image is included in the figure.


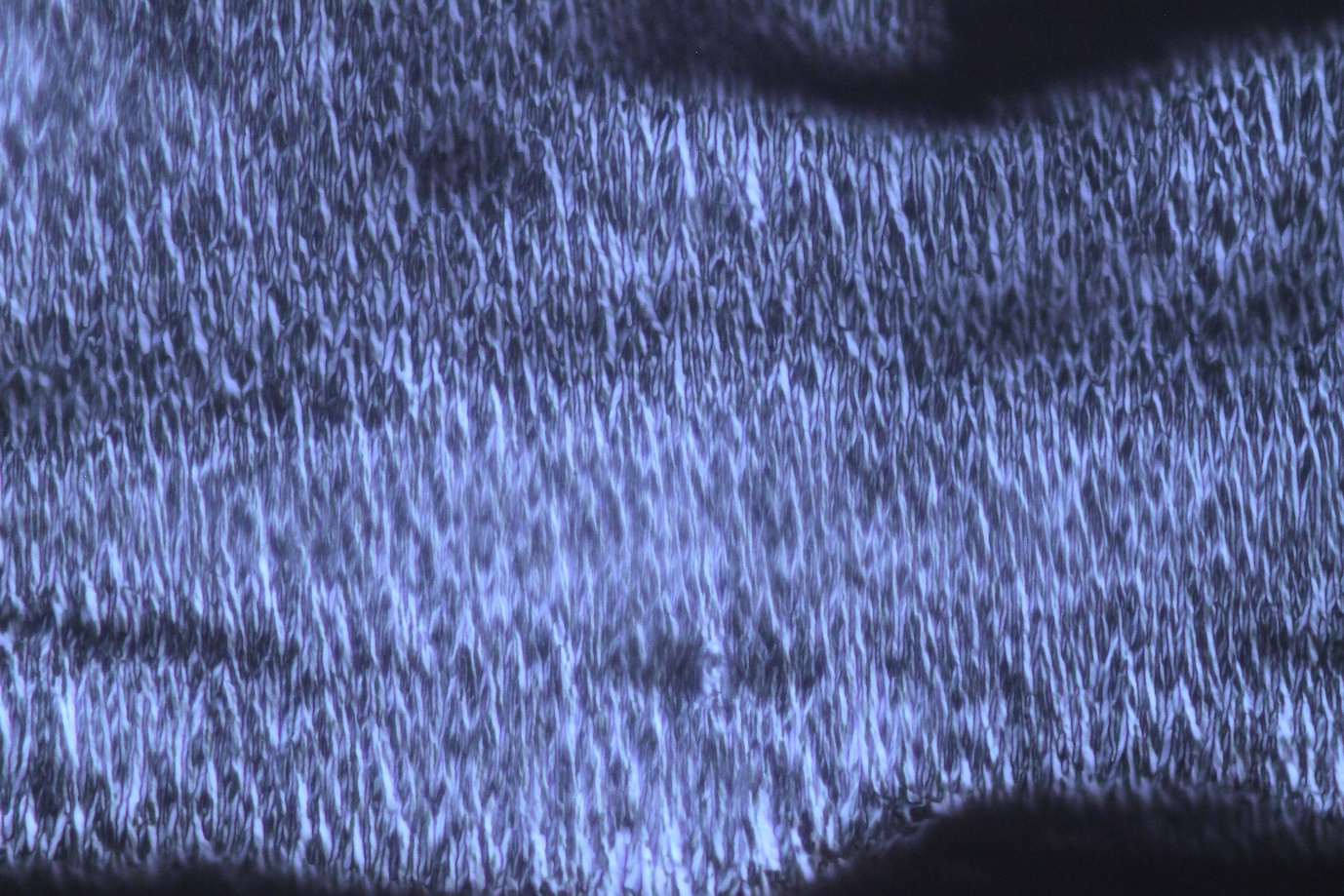


Original Image for Figure 4: (E/L)Go60, 1 hour; middle part of the image is included in the figure.


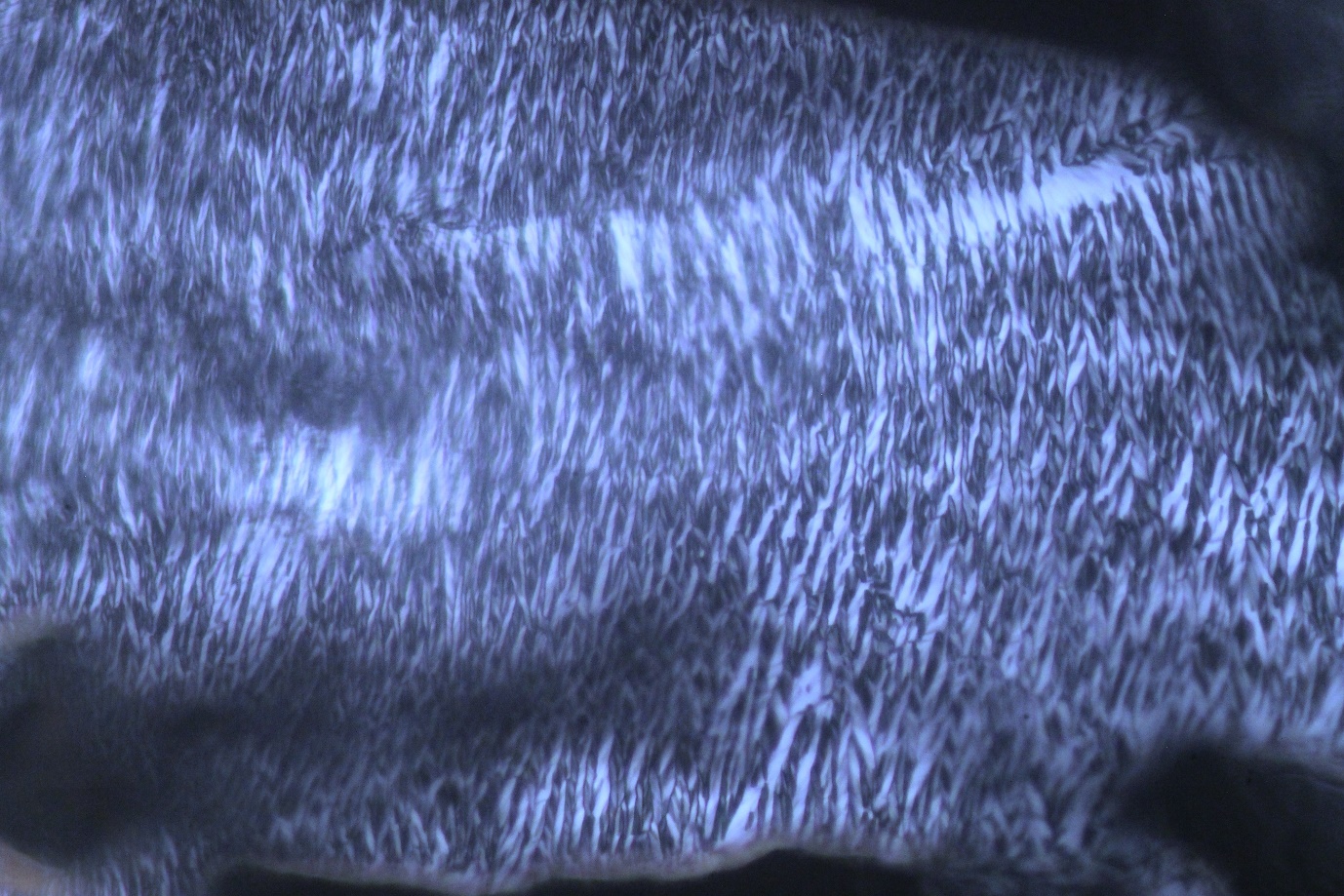


Original Image for Figure 4: (E/L)Go60, 24 hours; middle part of the image is included in the figure.


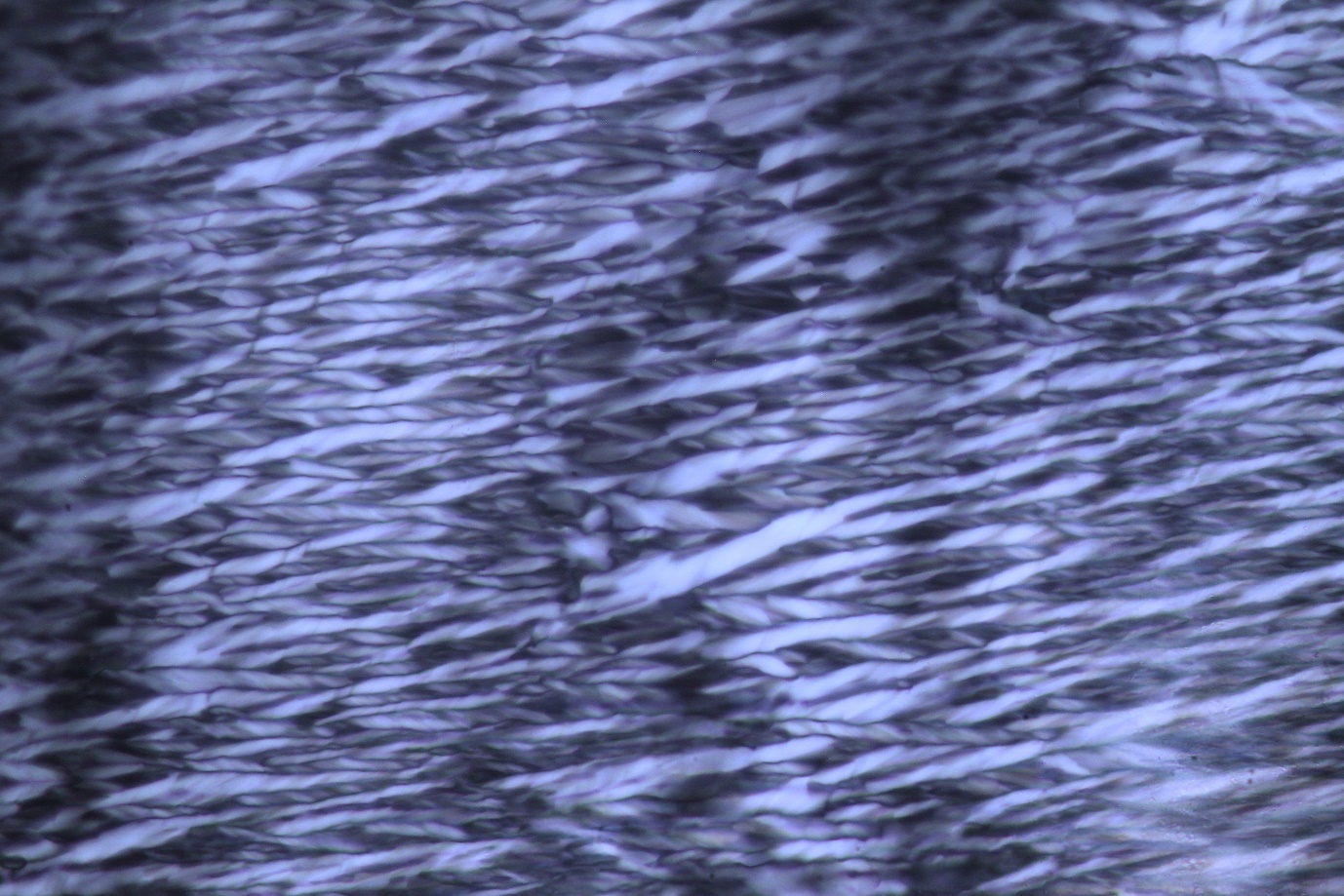


Original Image for Figure 4: (E/L)Go60, 72 hours; middle part of the image is included in the figure.


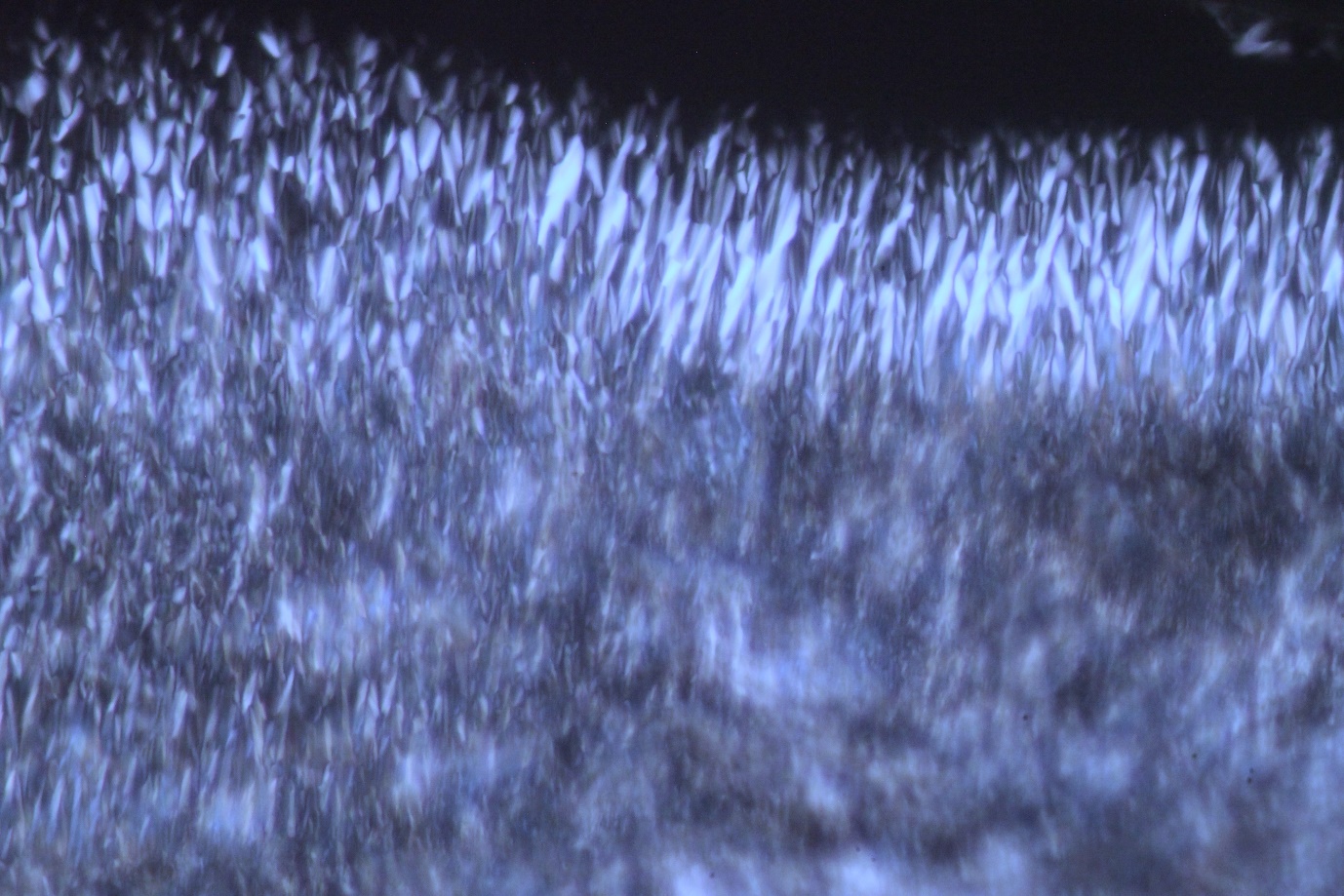


Original Image for Figure 4: (E/L)Go60, 7 days; middle part of the image is included in the figure.


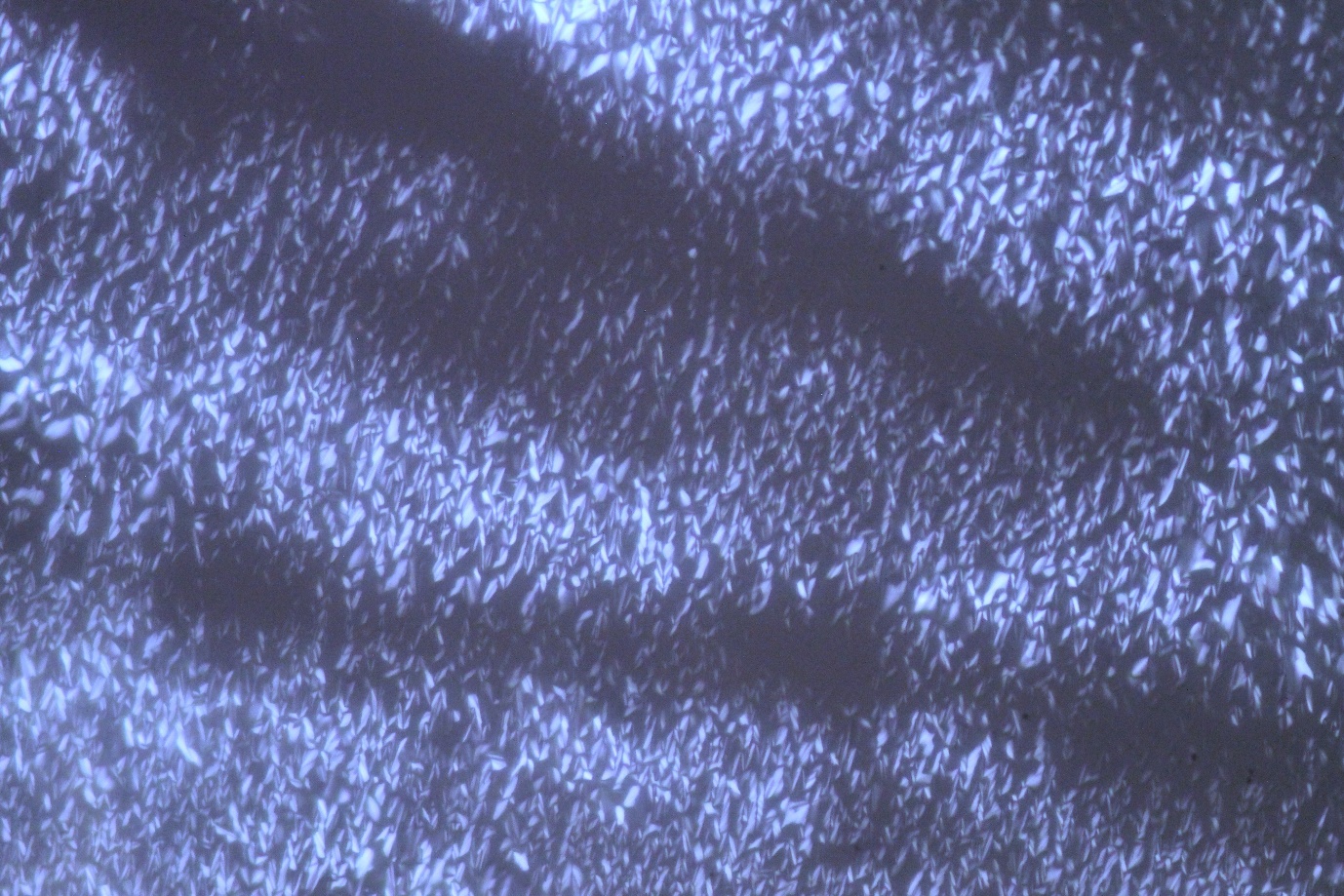


Original Image for Figure 4: (E/L)Go60, 14 days; middle part of the image is included in the figure.


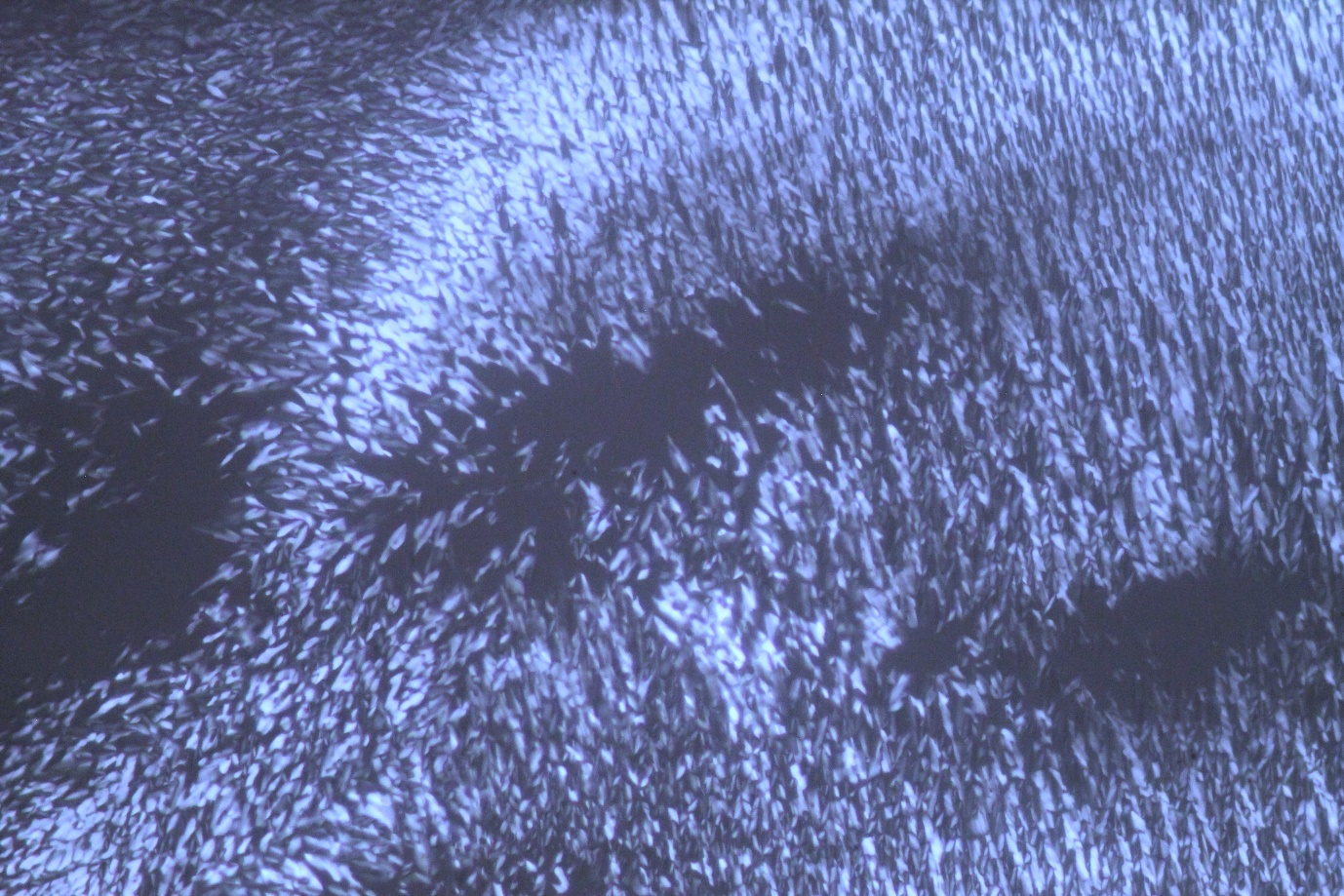


Original Image for Figure 4: (E/L)Gl60, 1 hour; middle part of the image is included in the figure.


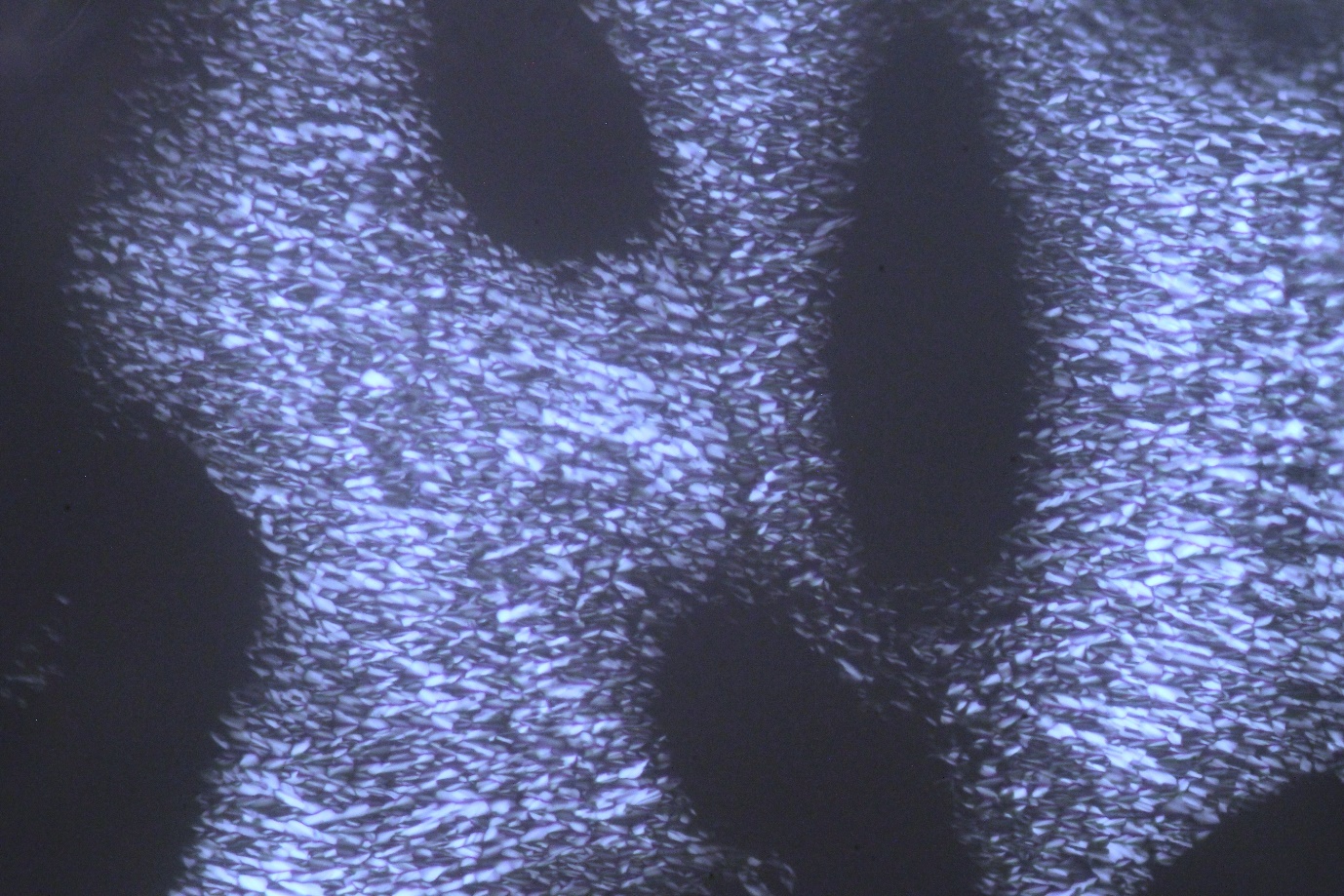


Original Image for Figure 4: (E/L)Gl60, 24 hours; middle part of the image is included in the figure.


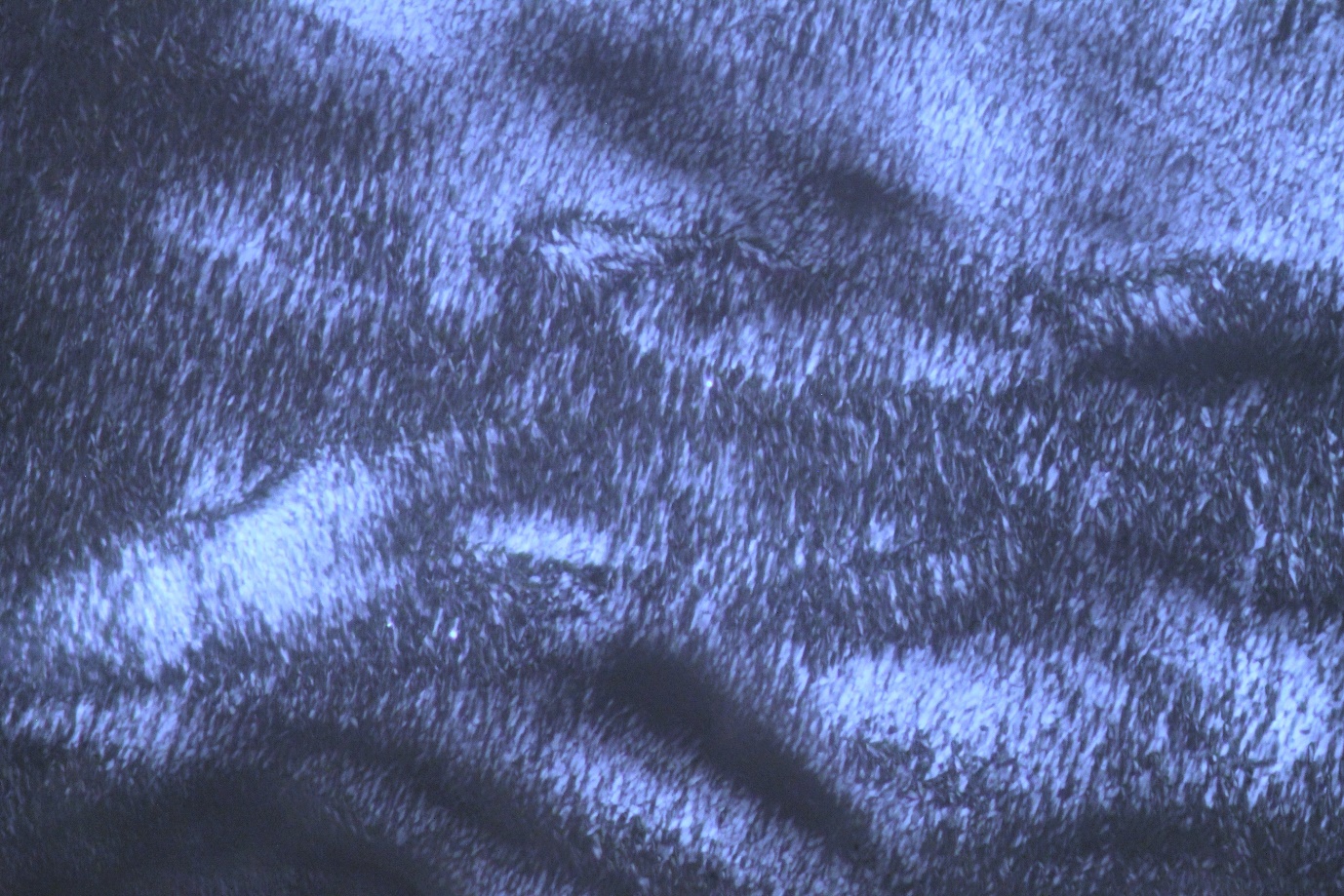


Original Image for Figure 4: (E/L)Gl60, 72 hours; middle part of the image is included in the figure.


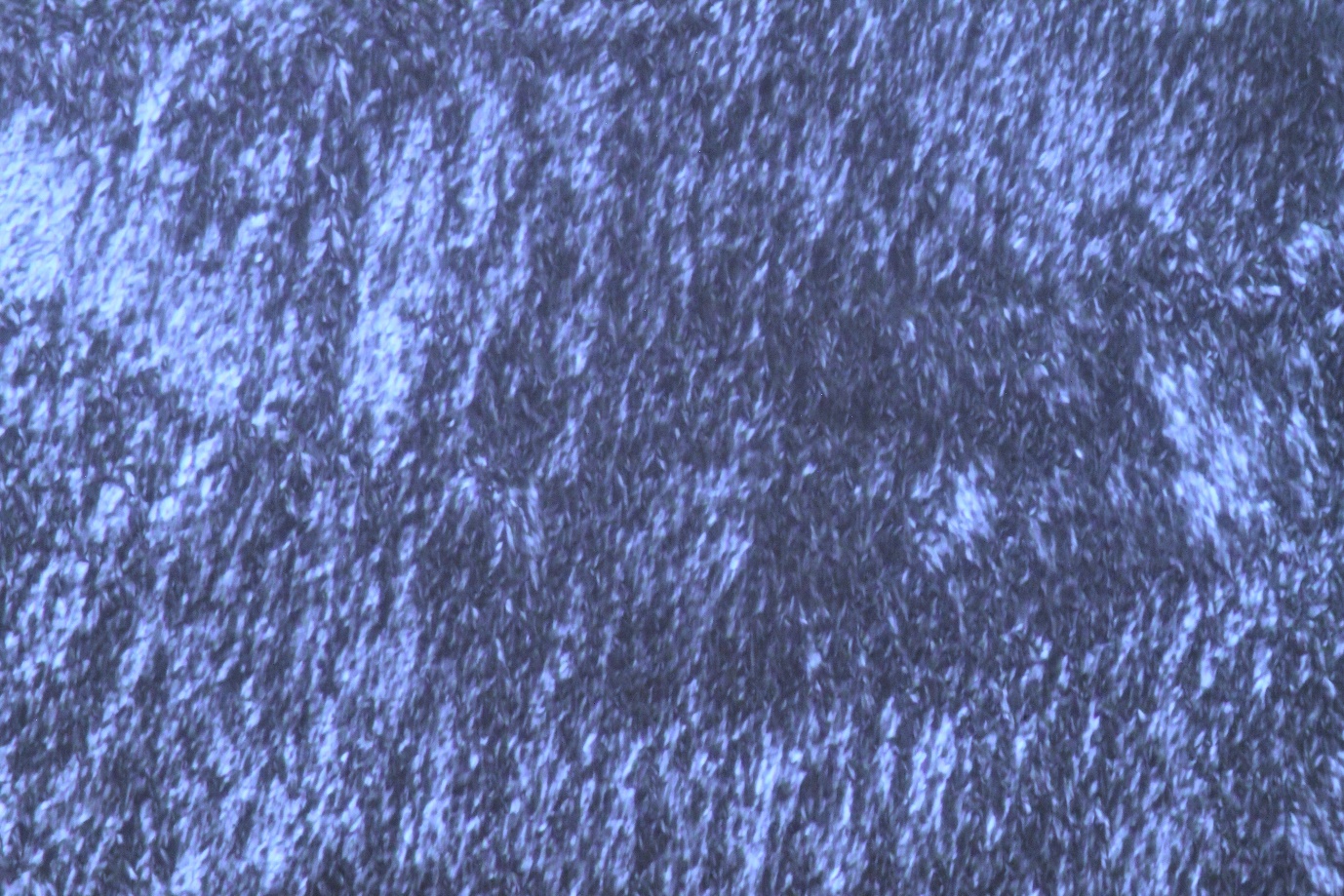


Original Image for Figure 4: (E/L)Gl60, 7 days; middle part of the image is included in the figure.


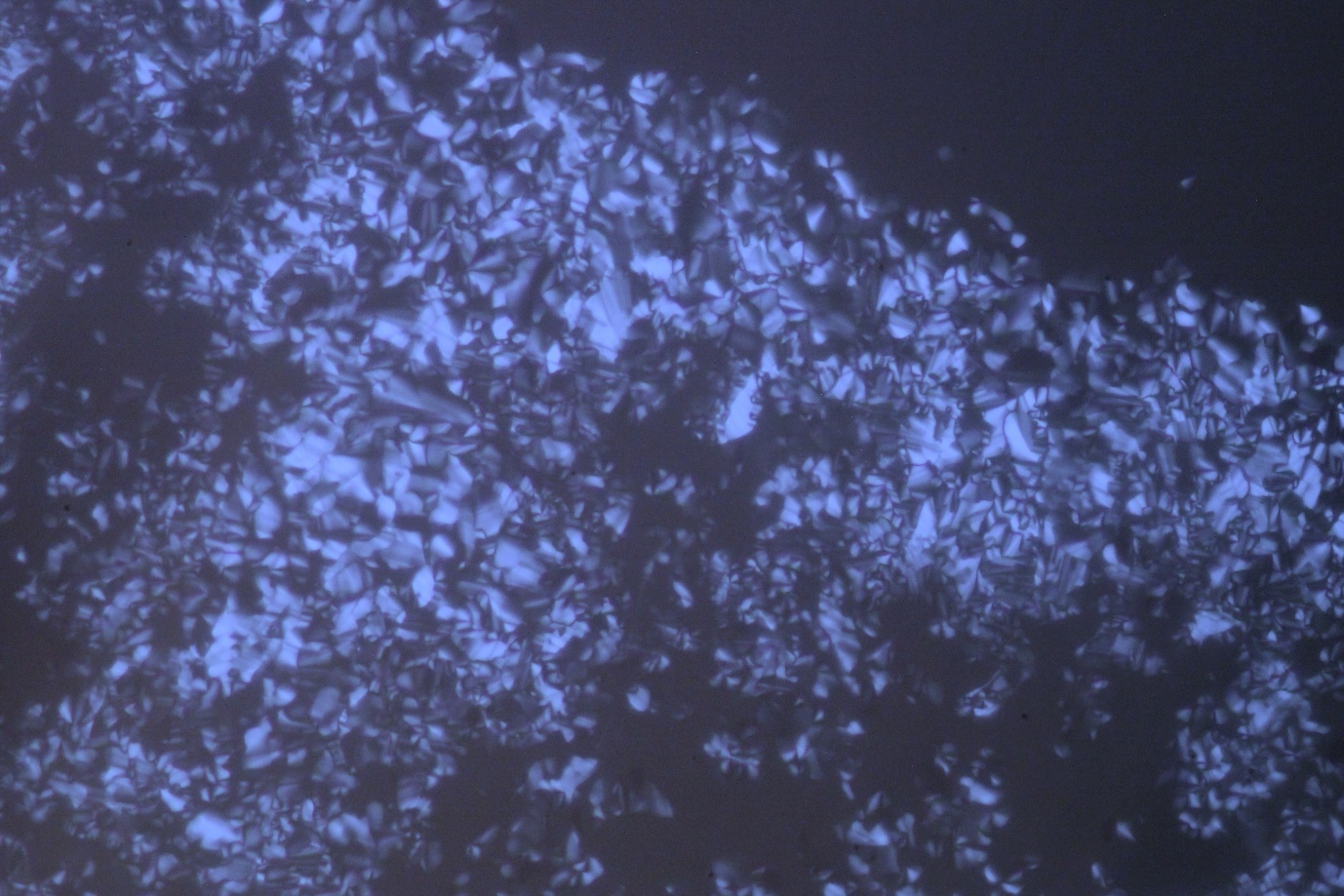


Original Image for Figure 4: (E/L)Gl60, 14 days; middle part of the image is included in the figure.


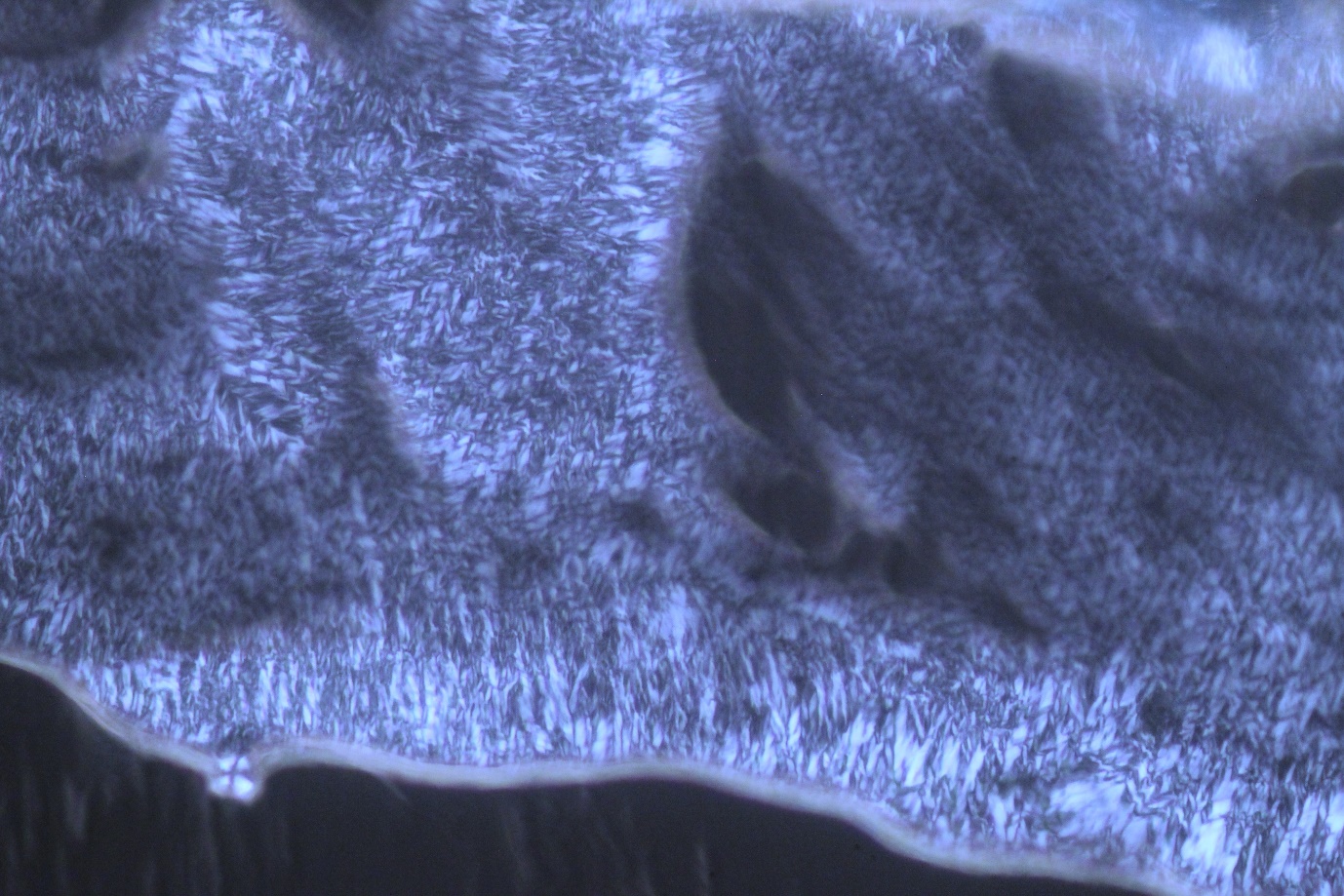


Original Image for Figure 4: (E/L)Go70, 1 hour; middle part of the image is included in the figure.


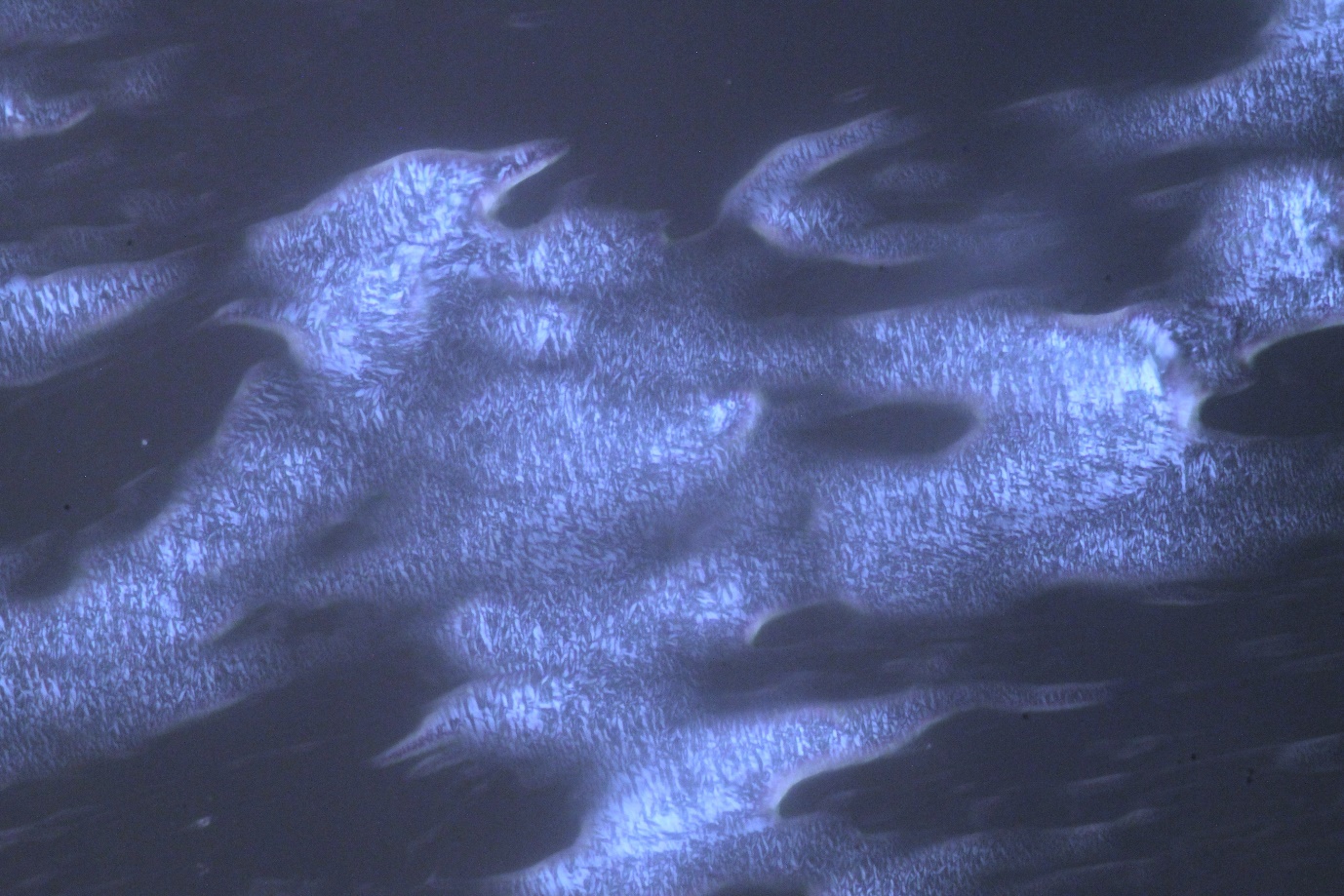


Original Image for Figure 4: (E/L)Go70, 24 hours; middle part of the image is included in the figure.


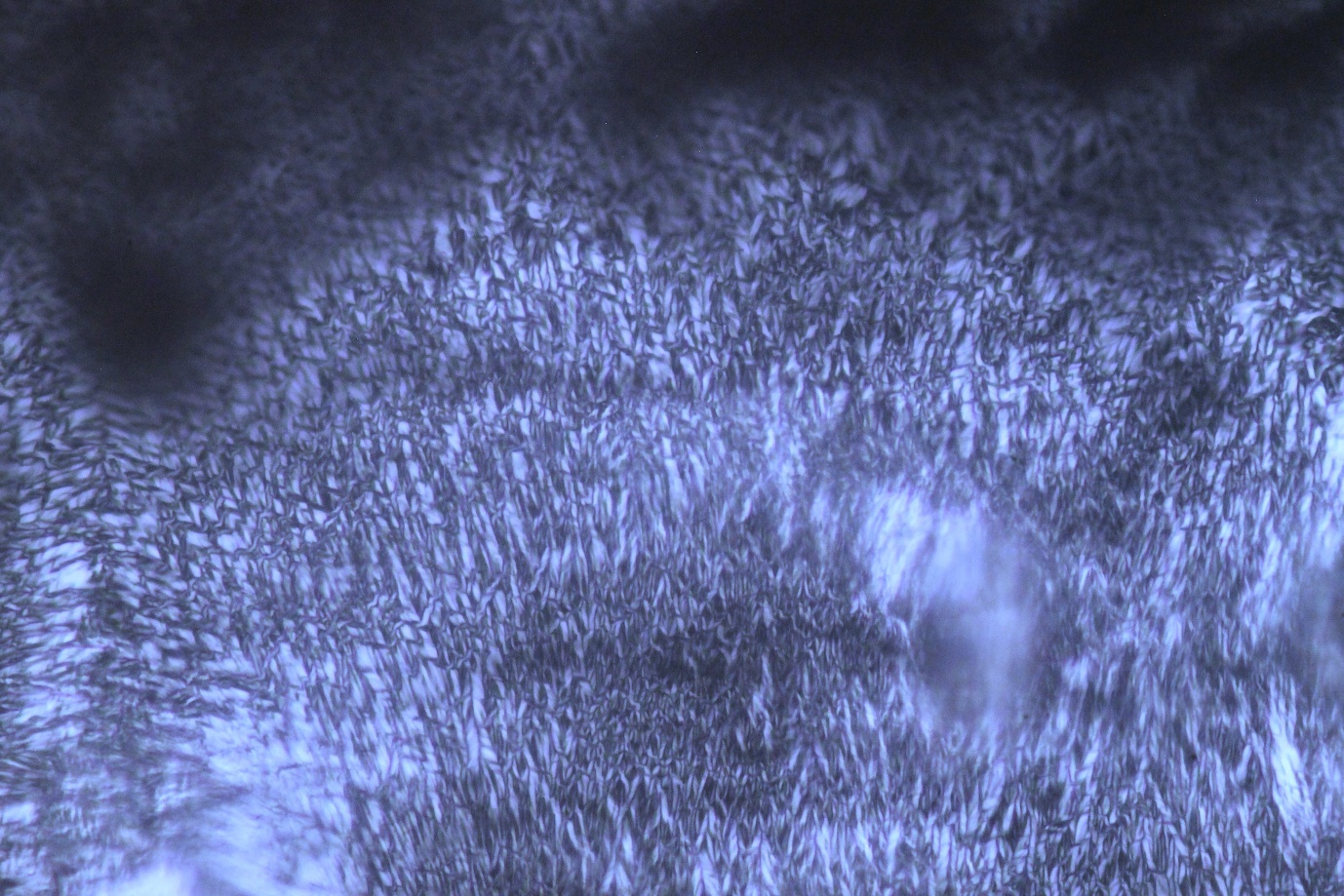


Original Image for Figure 4: (E/L)Go70, 72 hours; middle part of the image is included in the figure.


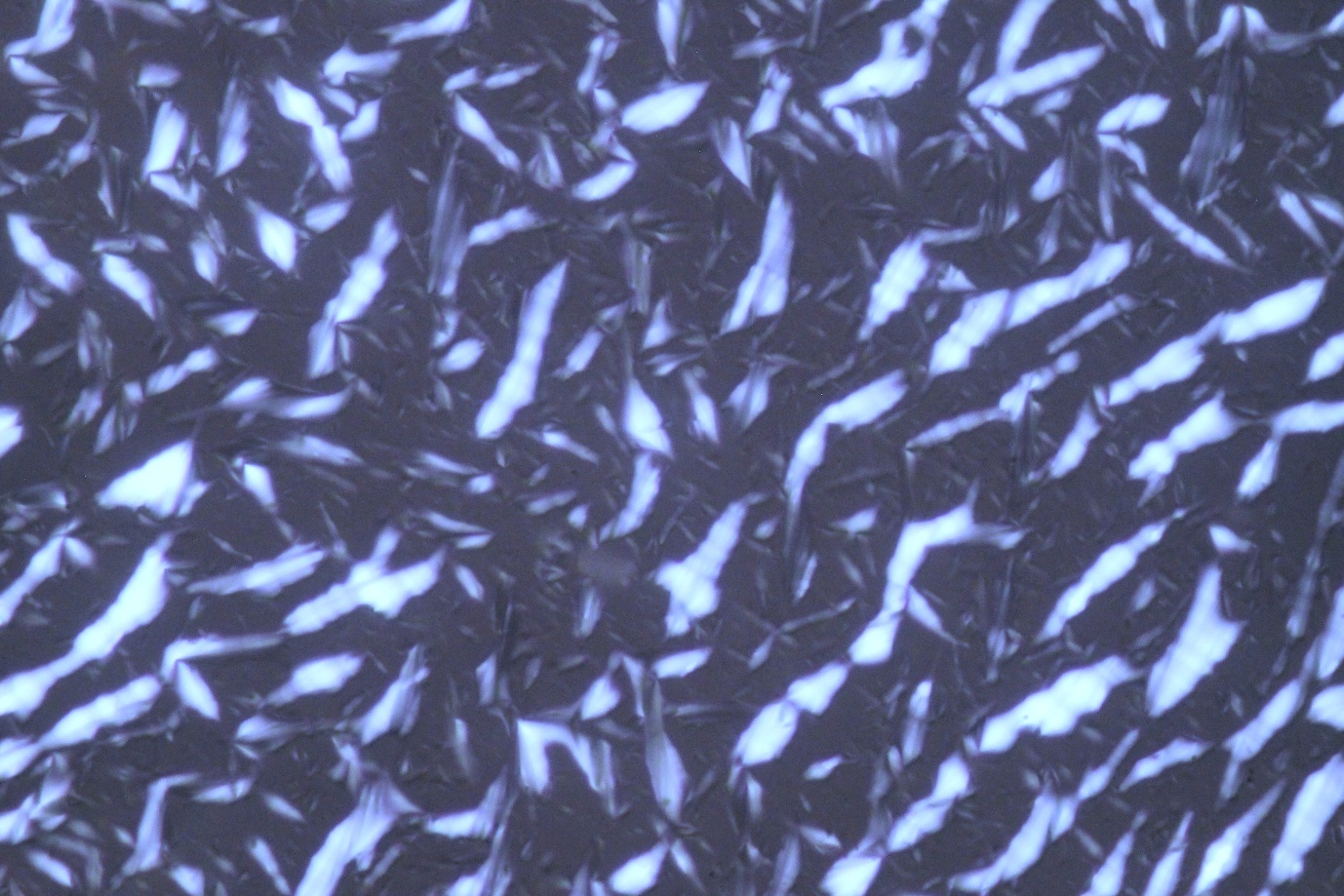


Original Image for Figure 4: (E/L)Go70, 7 days; middle part of the image is included in the figure.


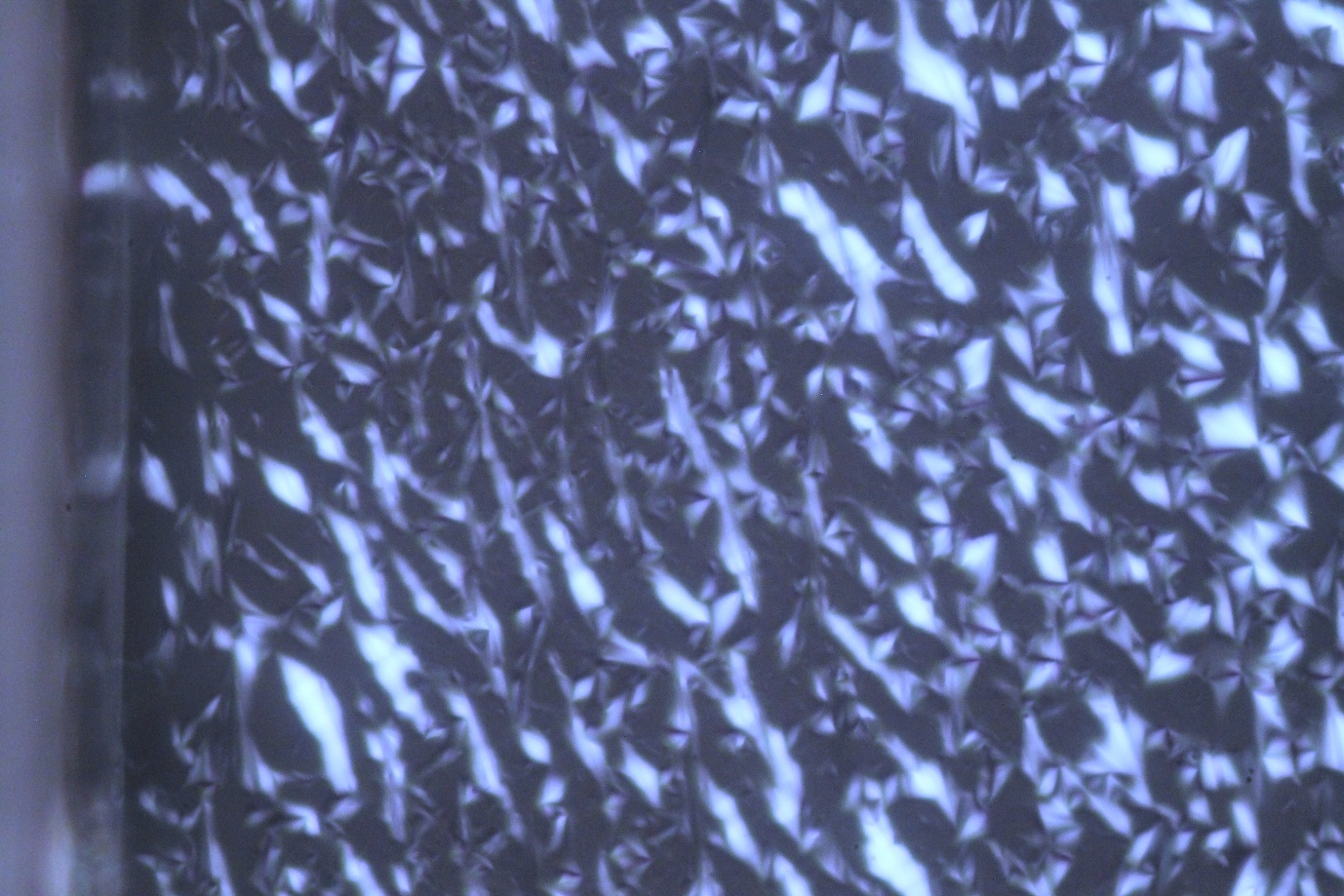


Original Image for Figure 4: (E/L)Go70, 14 days; middle part of the image is included in the figure.


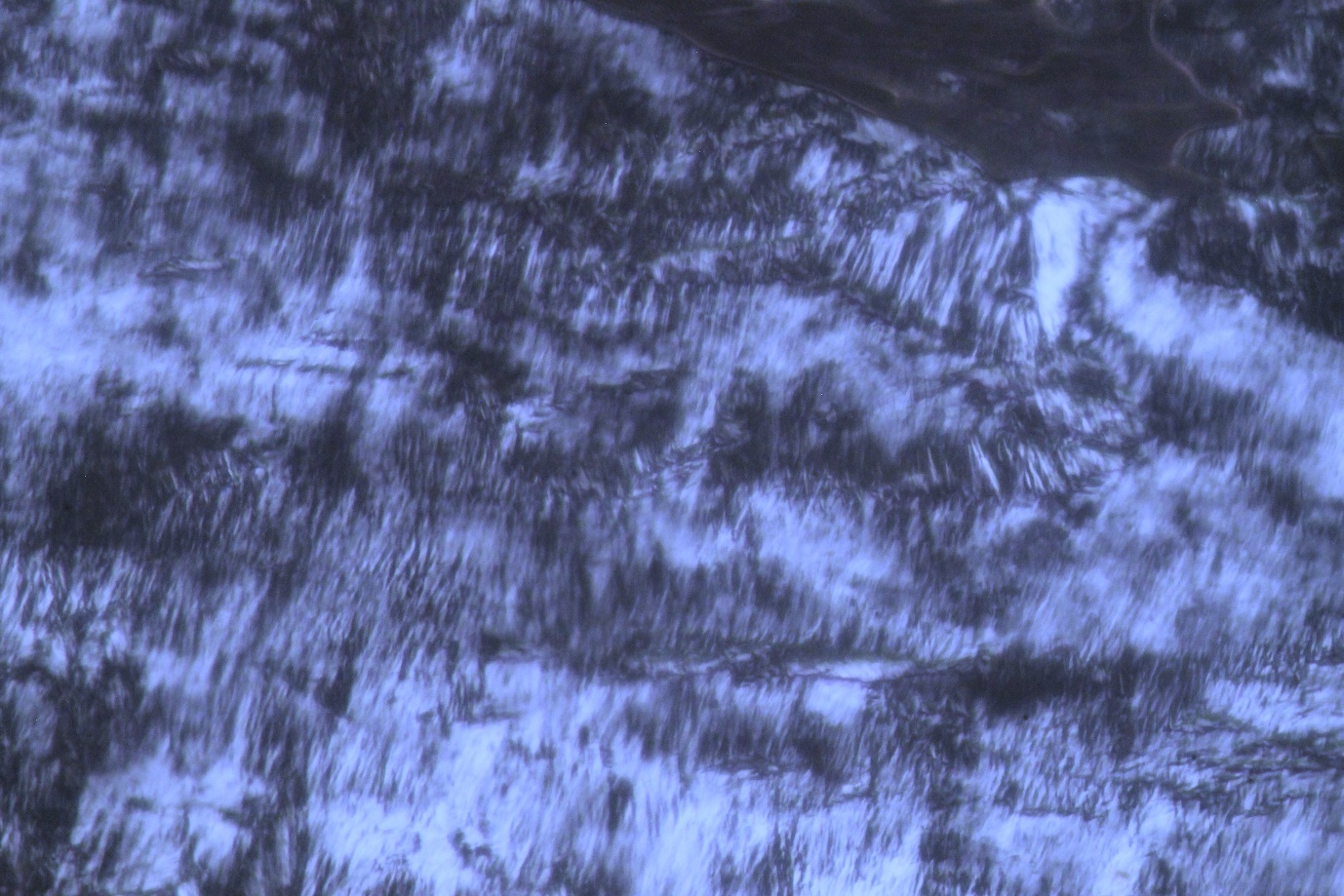


Original Image for Figure 4: (E/L)Gl70, 1 hour; middle part of the image is included in the figure.


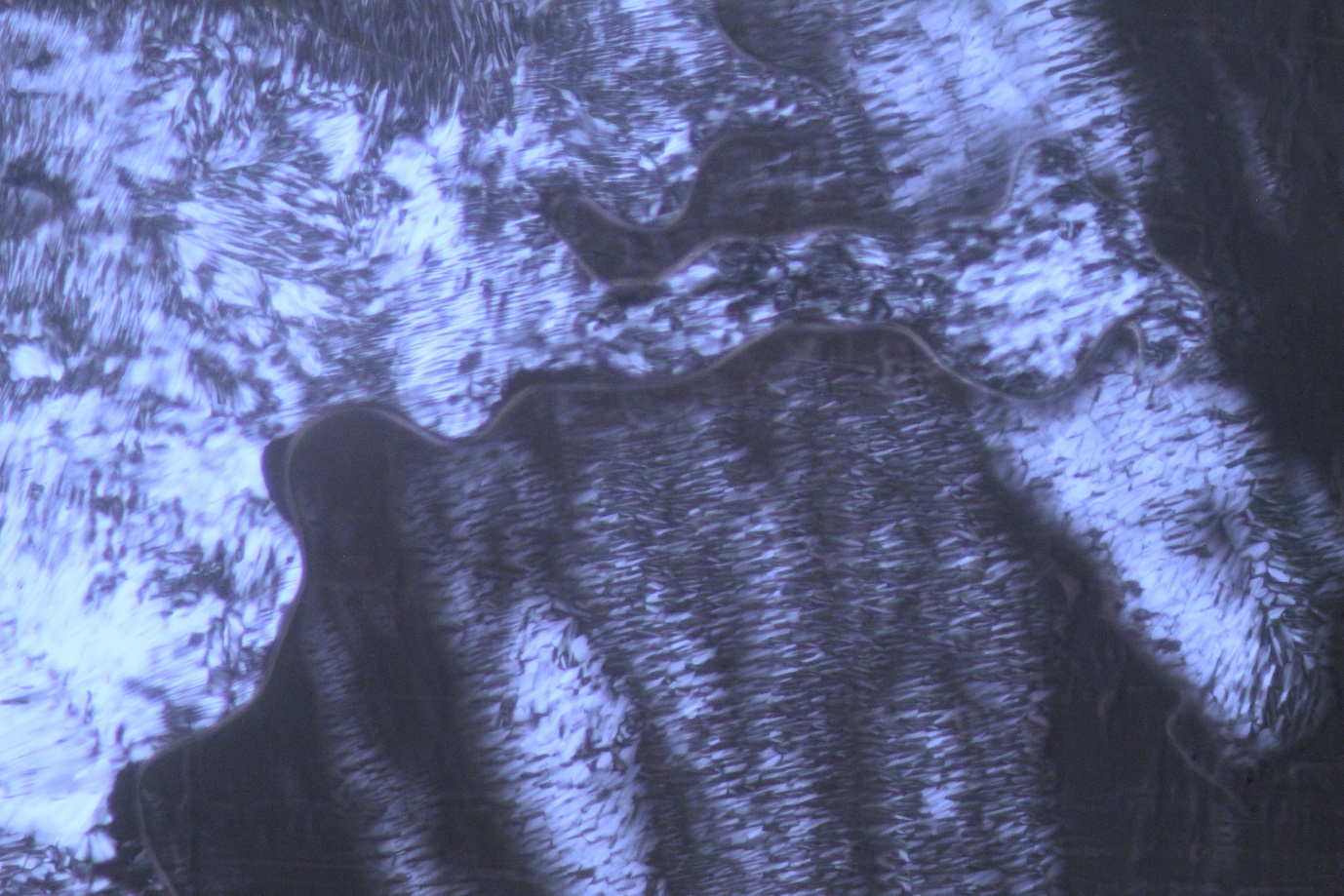


Original Image for Figure 4: (E/L)Gl70, 24 hours; middle part of the image is included in the figure.


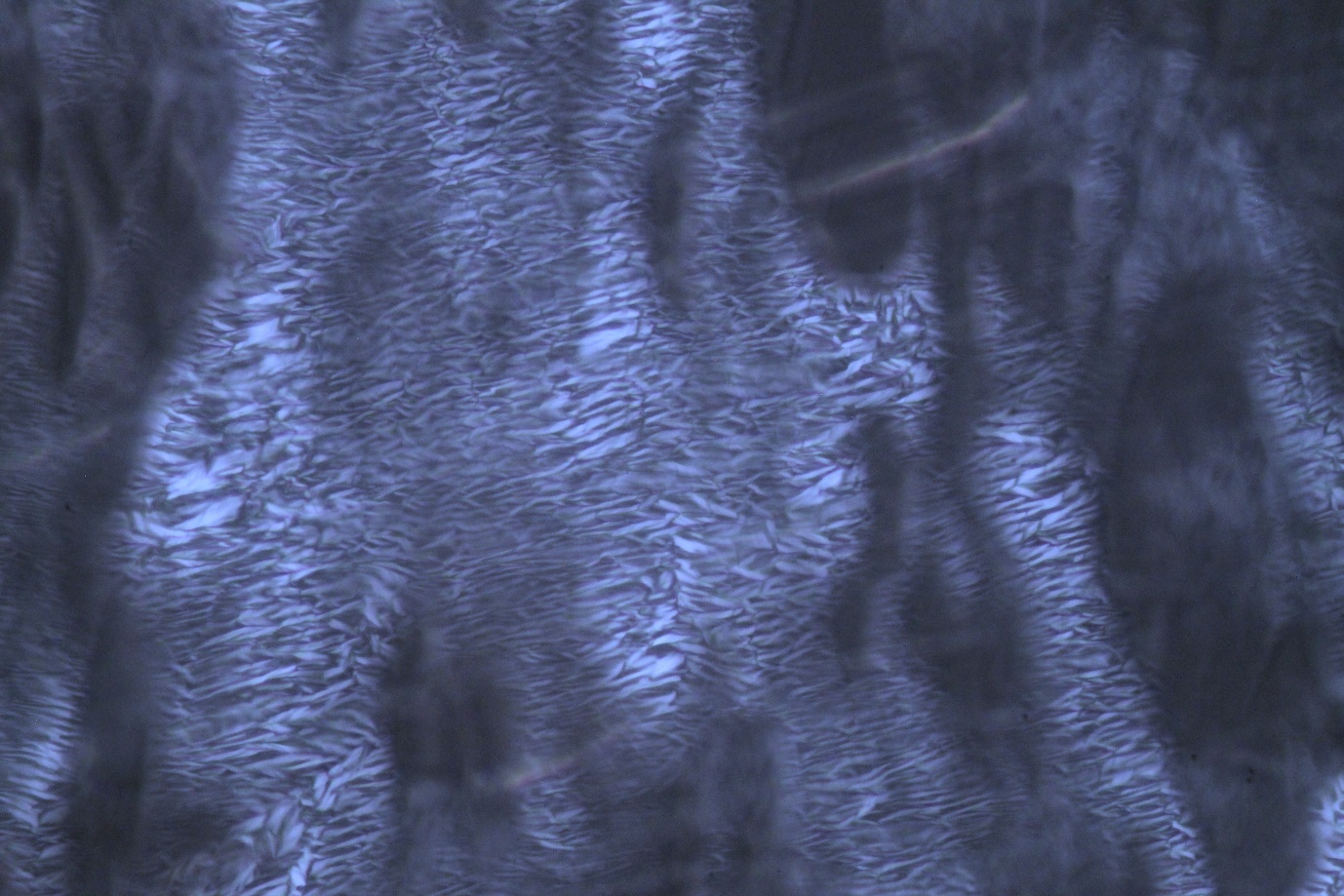


Original Image for Figure 4: (E/L)Gl70, 72 hours; middle part of the image is included in the figure.


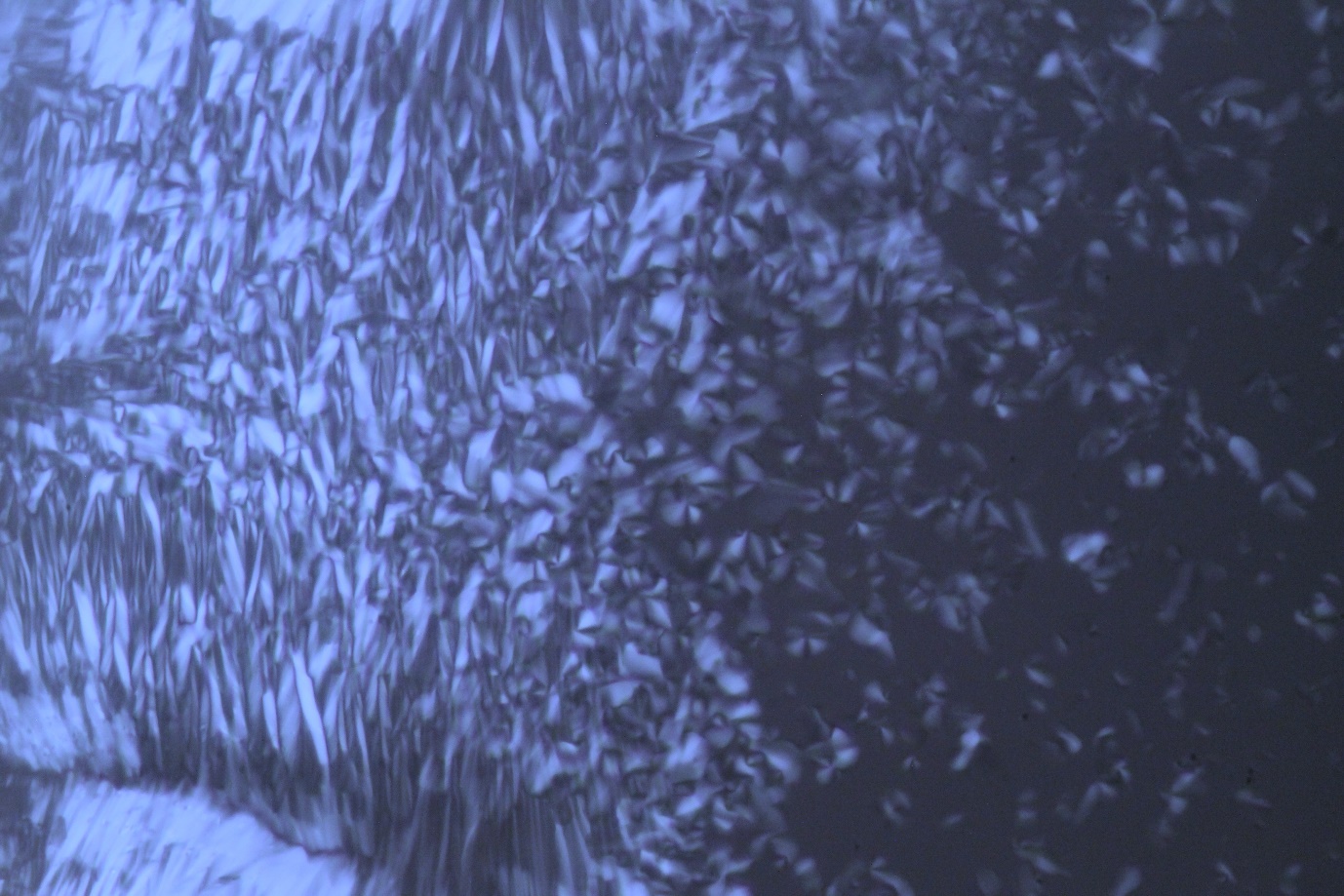


Original Image for Figure 4: (E/L)Gl70, 7 days; middle part of the image is included in the figure.


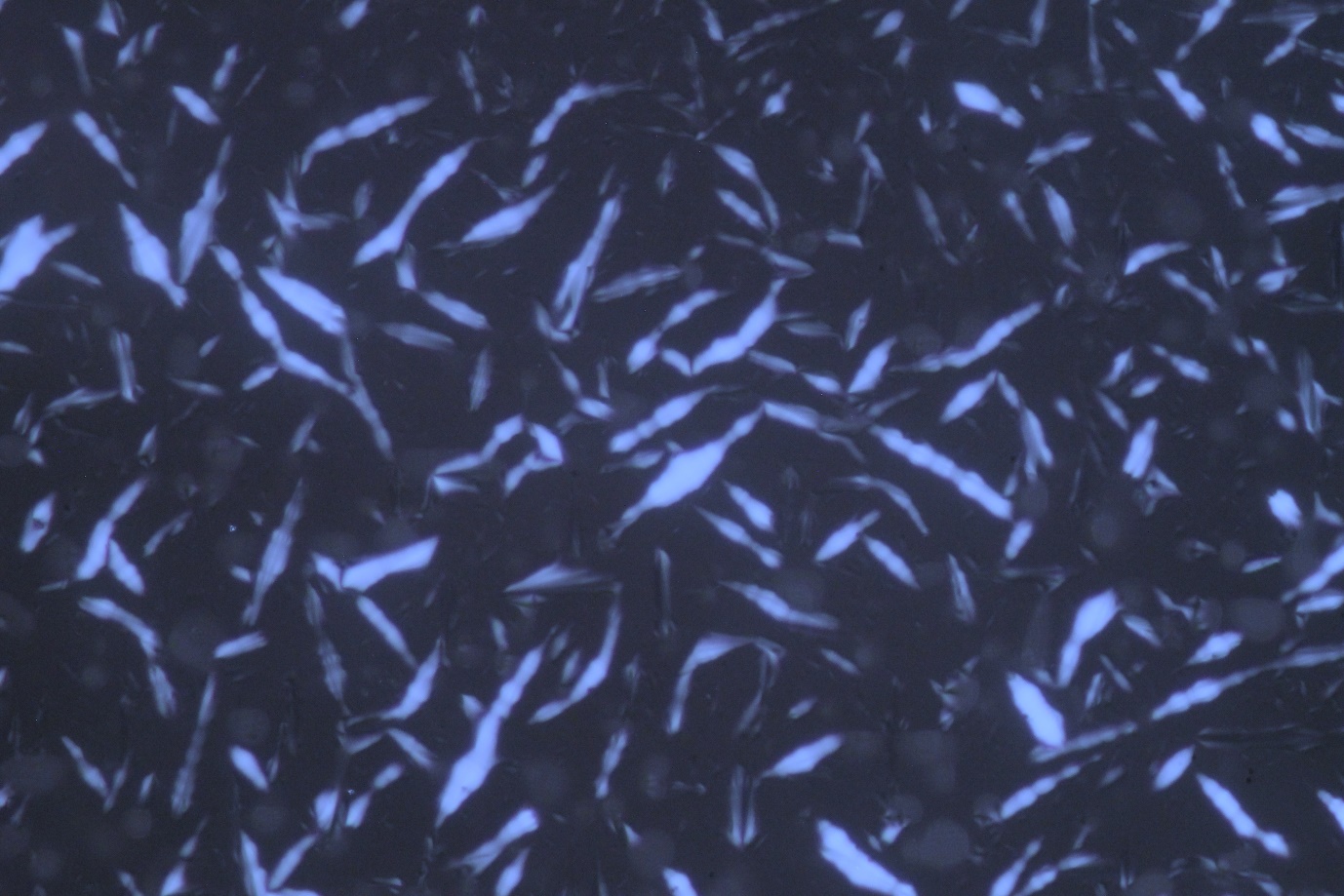


Original Image for Figure 4: (E/L)Gl70, 14 days; middle part of the image is included in the figure.


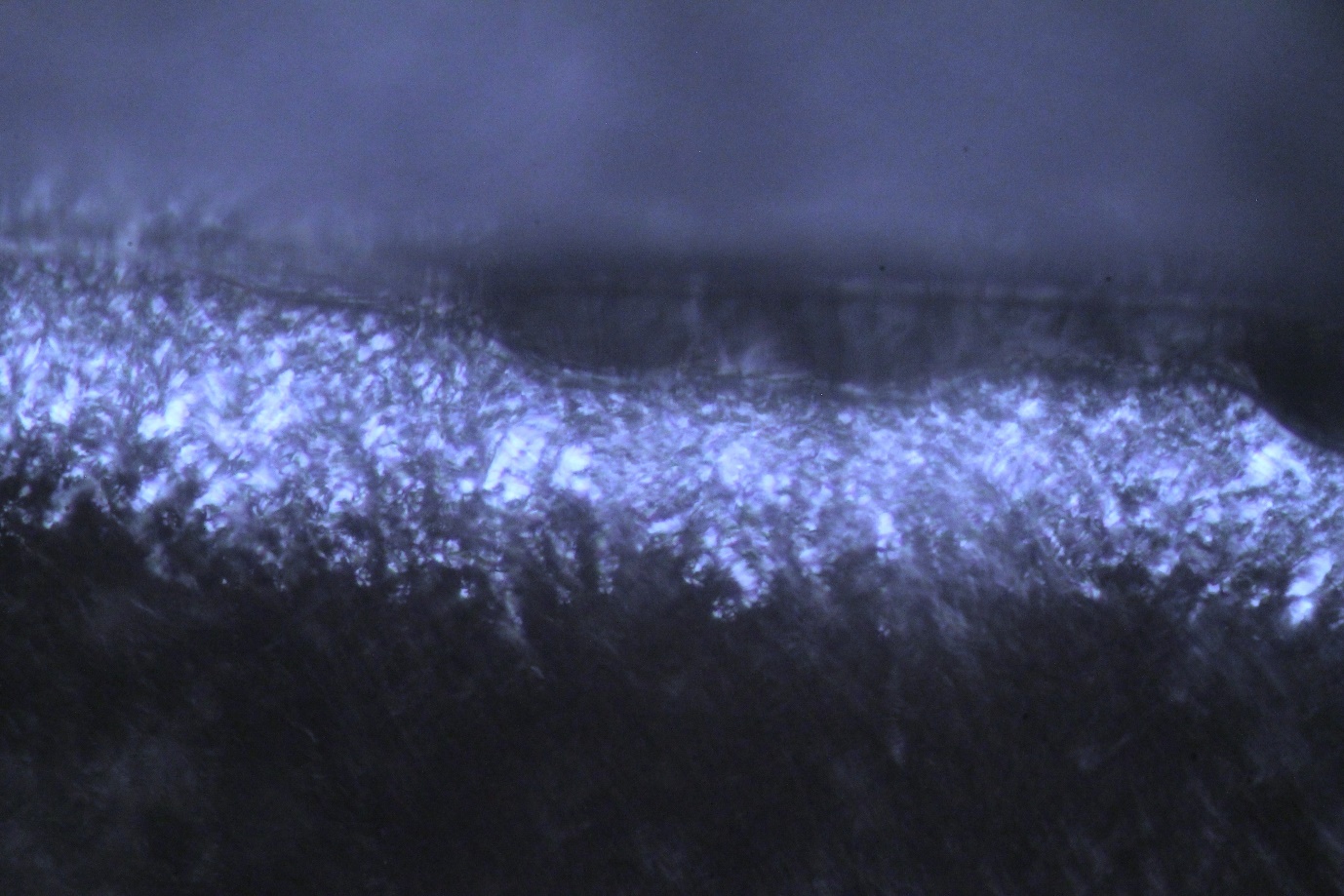


Original Image for Figure 4: (E/L)Go80, 1 hour; middle part of the image is included in the figure.


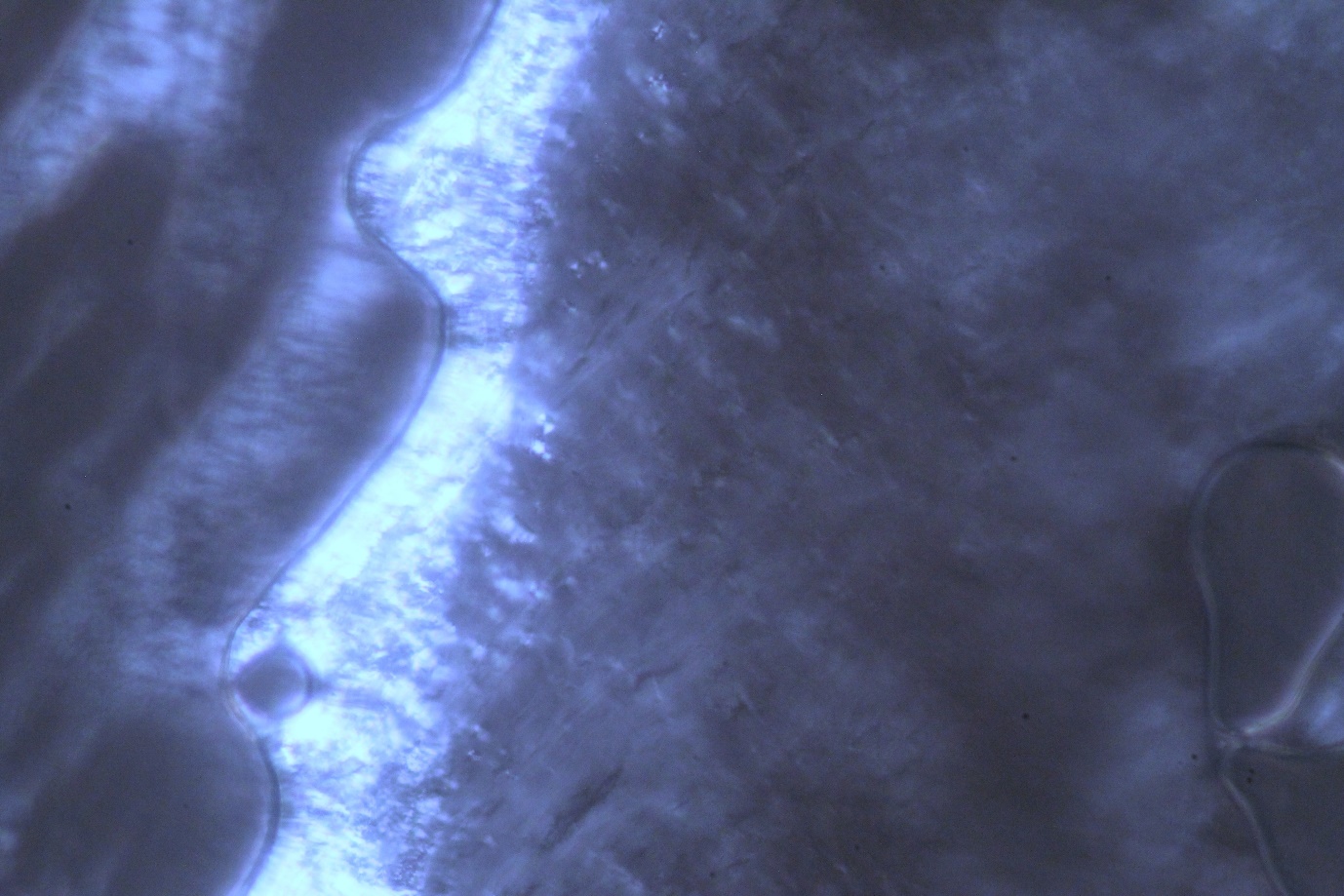


Original Image for Figure 4: (E/L)Go80, 24 hours; middle part of the image is included in the figure.


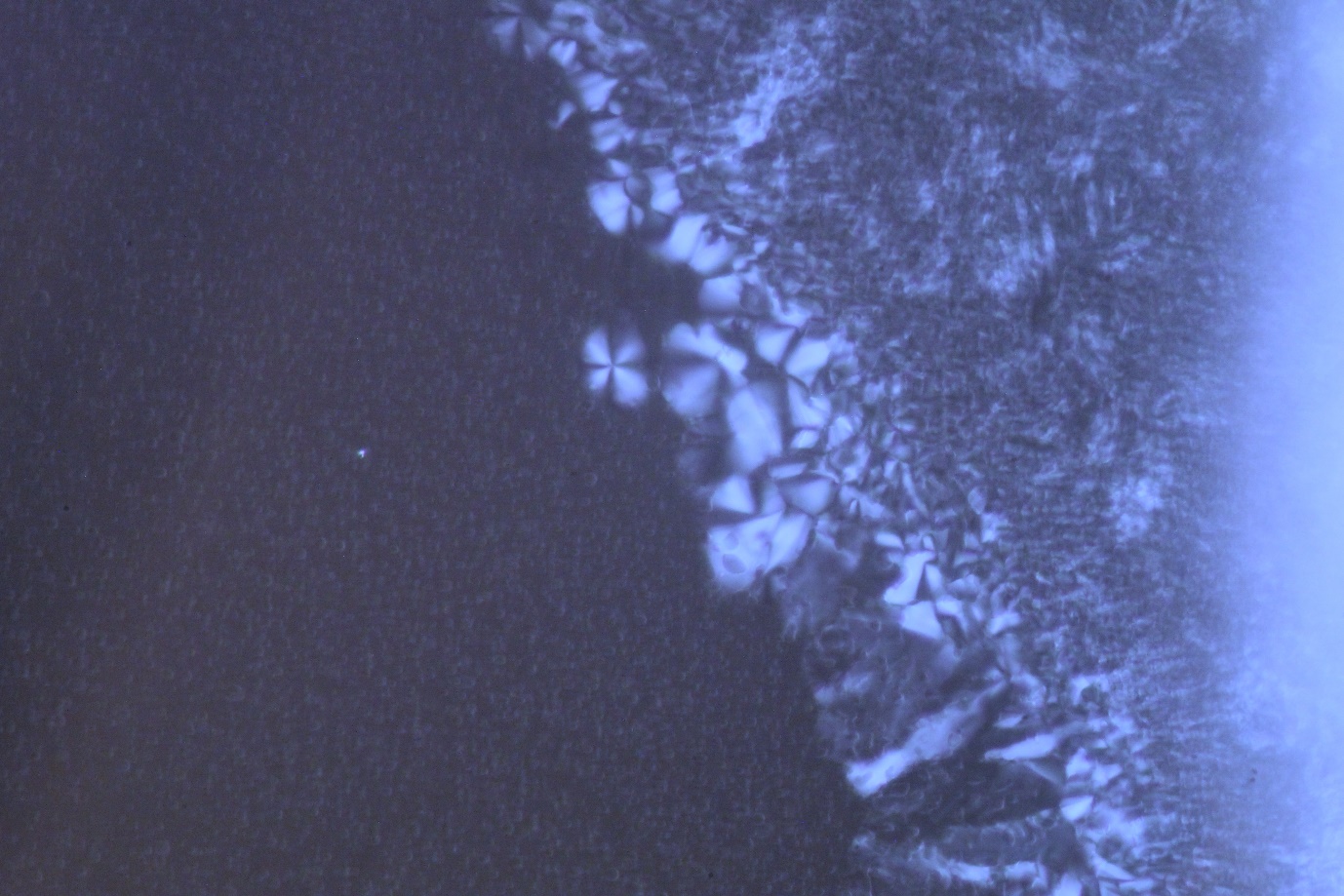


Original Image for Figure 4: (E/L)Go80, 72 hours; middle part of the image is included in the figure.


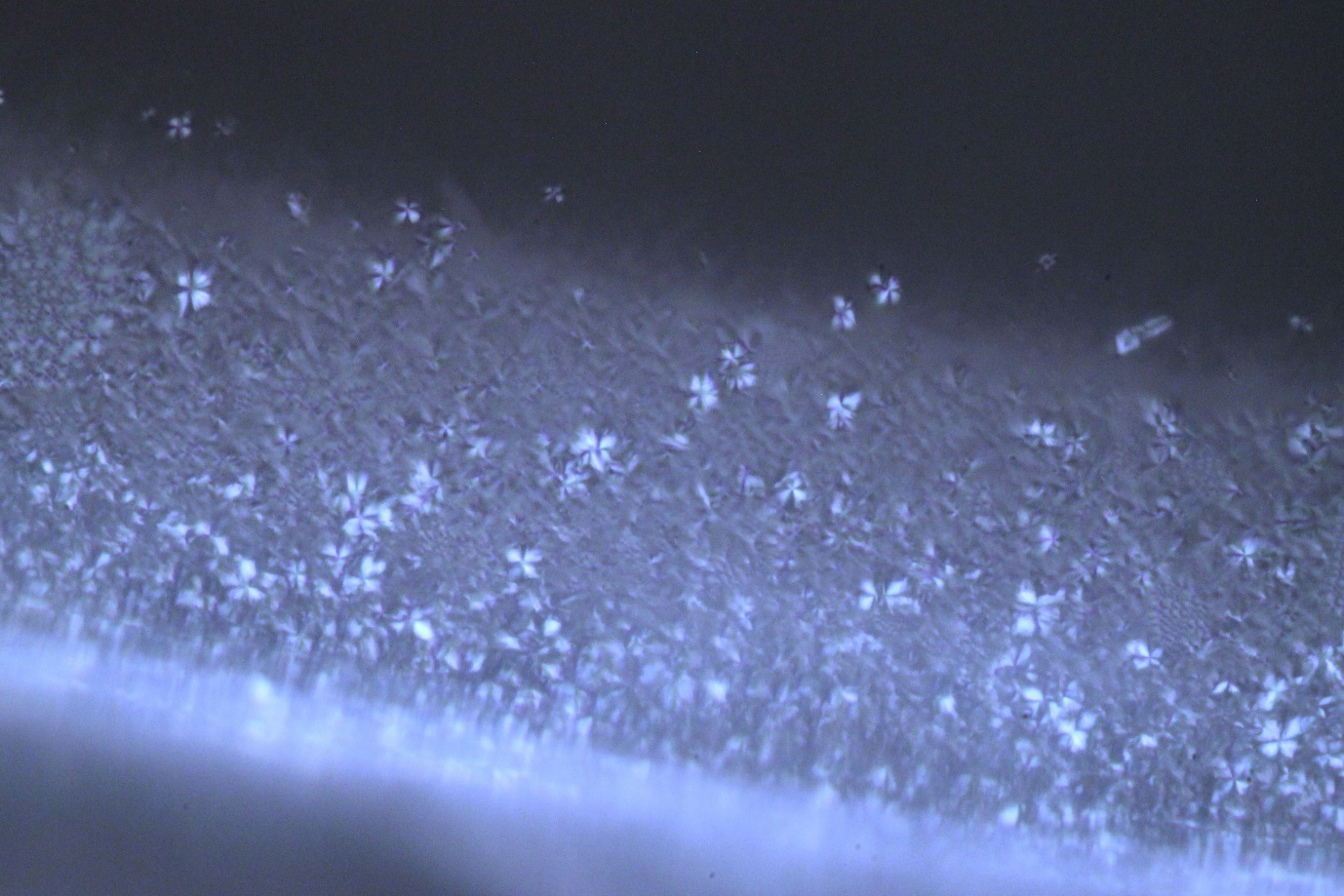


Original Image for Figure 4: (E/L)Go80, 7 days; middle part of the image is included in the figure.


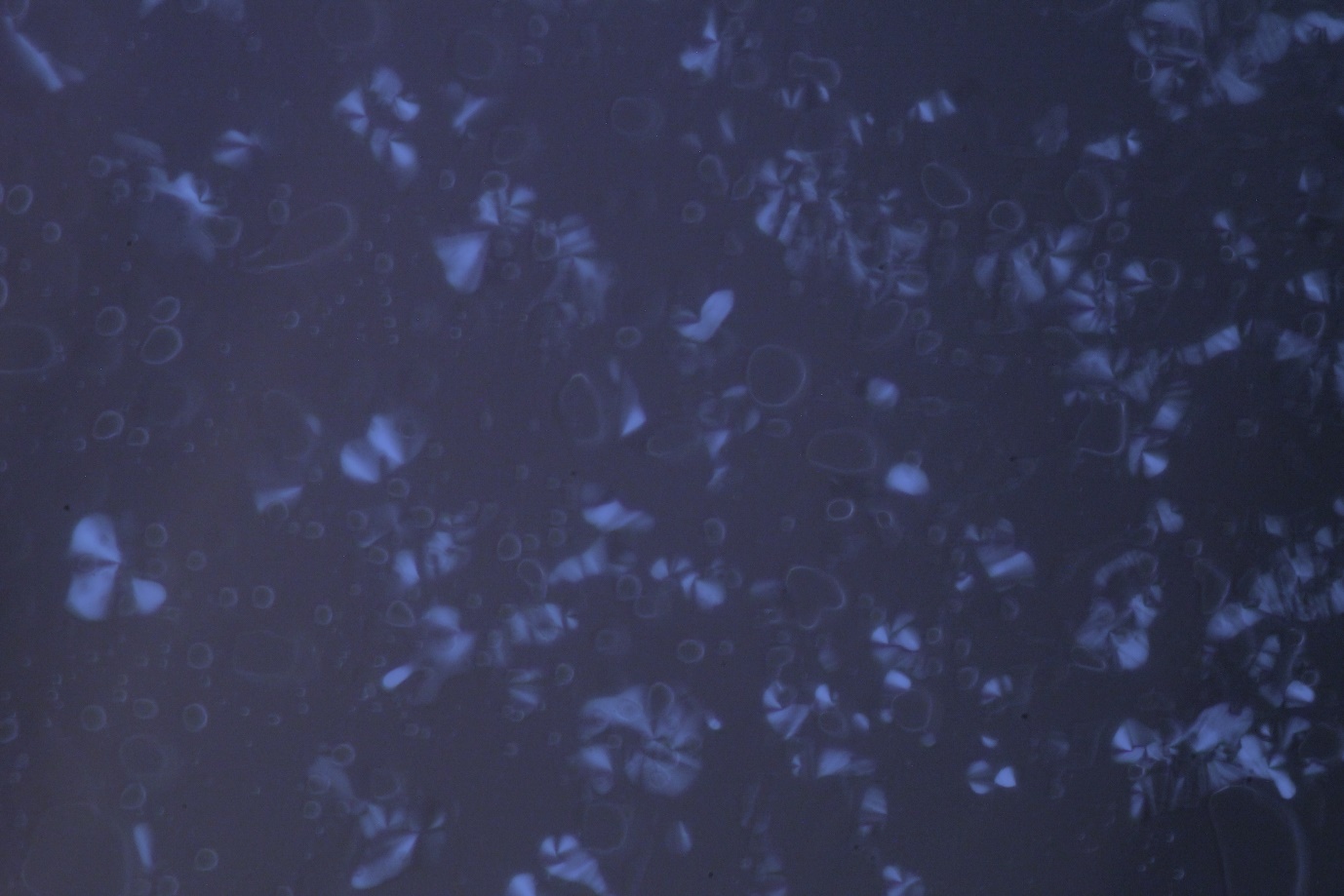


Original Image for Figure 4: (E/L)Go80, 14 days; middle part of the image is included in the figure.


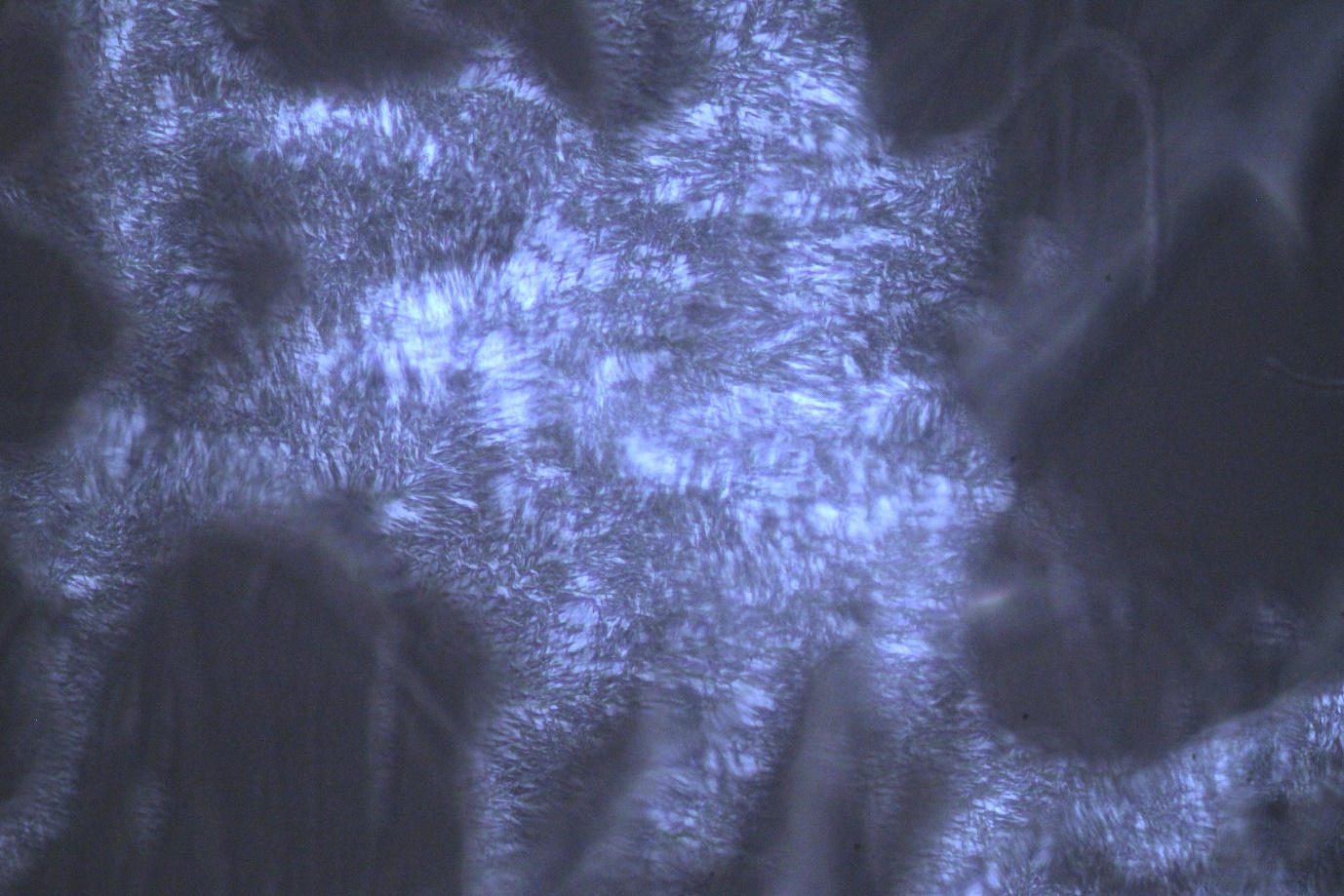


Original Image for Figure 4: (E/L)Gl80, 1 hour; middle part of the image is included in the figure.


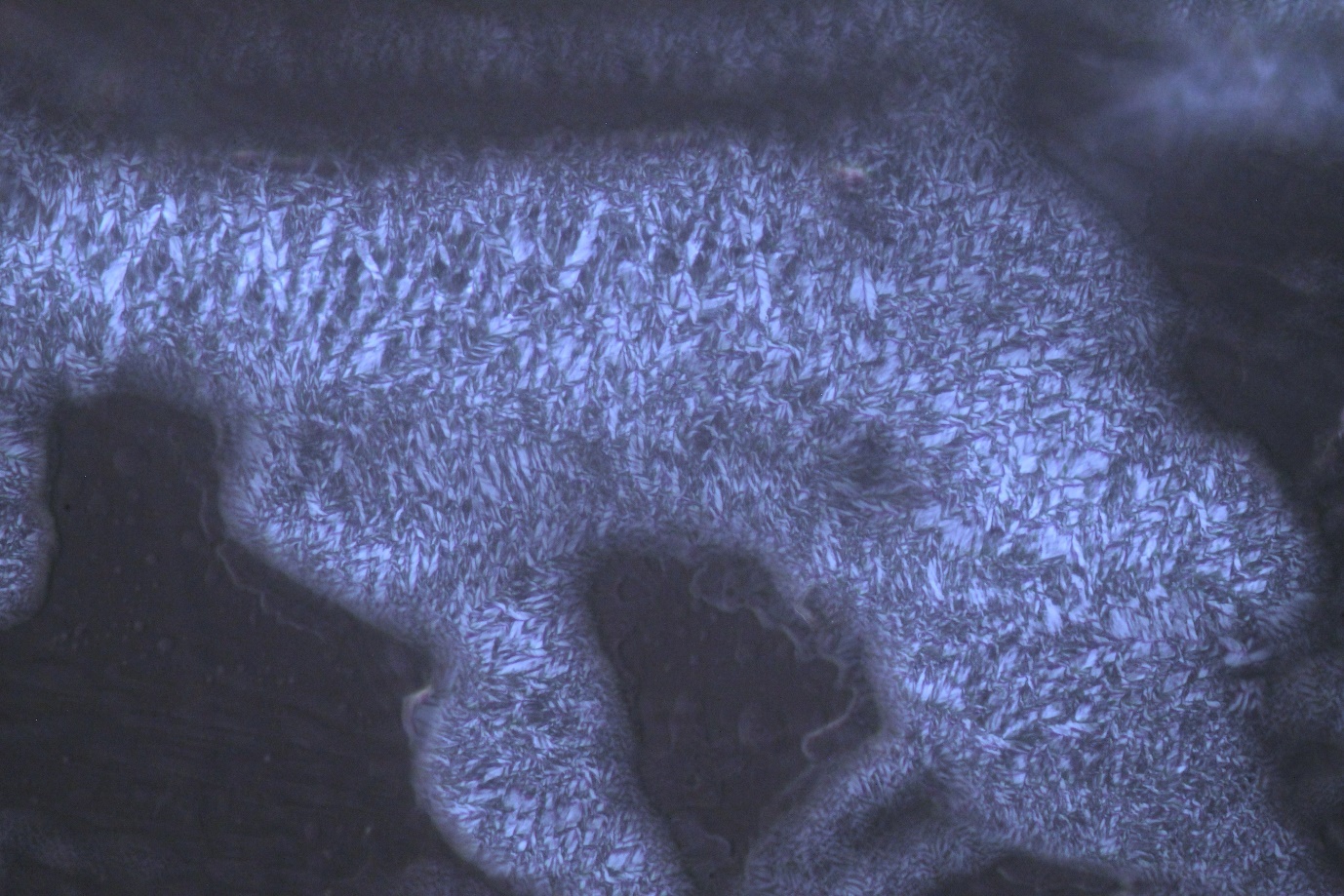


Original Image for Figure 4: (E/L)Gl80, 24 hours; middle part of the image is included in the figure.


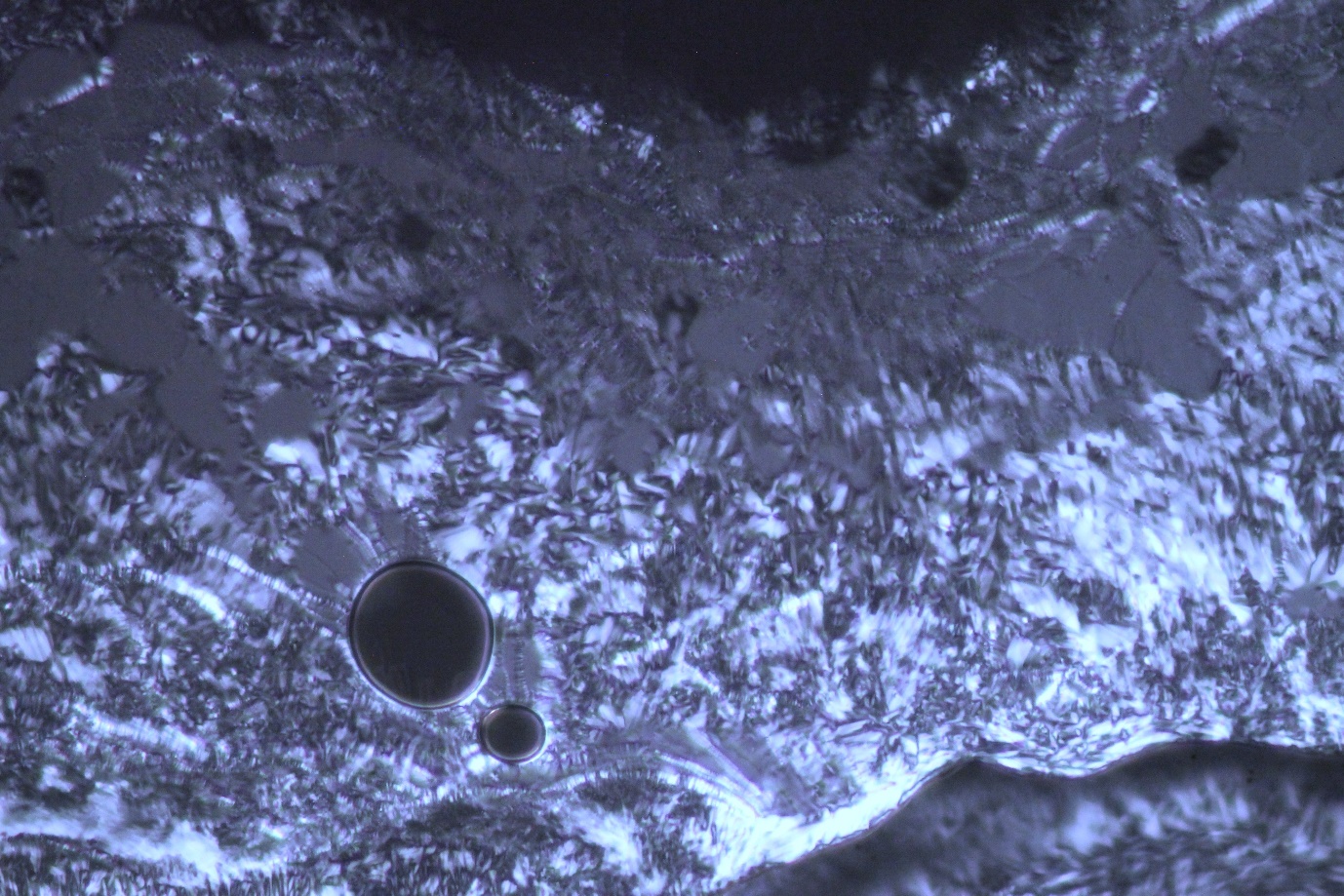


Original Image for Figure 4: (E/L)Gl80, 72 hours; middle part of the image is included in the figure.


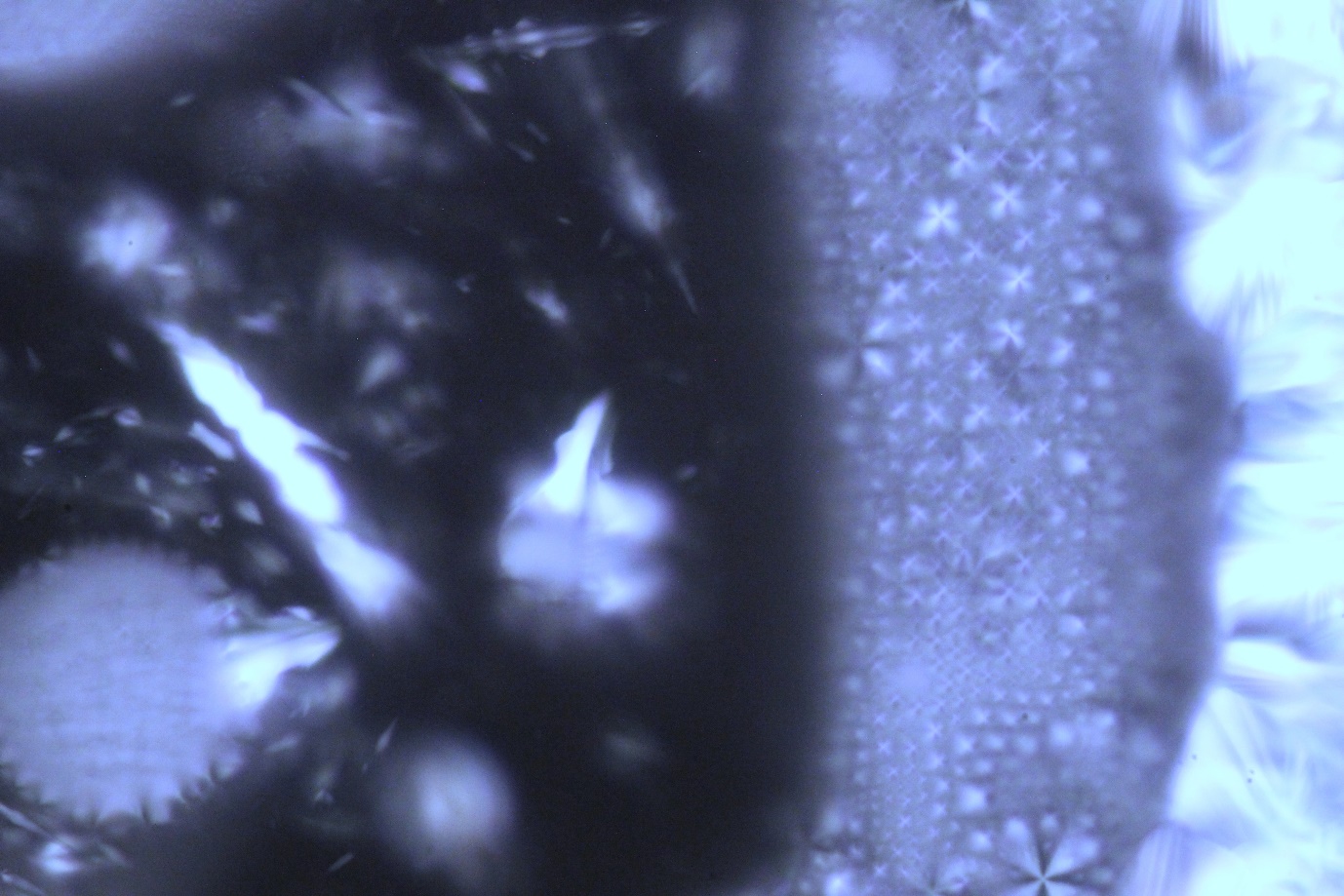


Original Image for Figure 4: (E/L)Gl80, 7 days; middle part of the image is included in the figure.


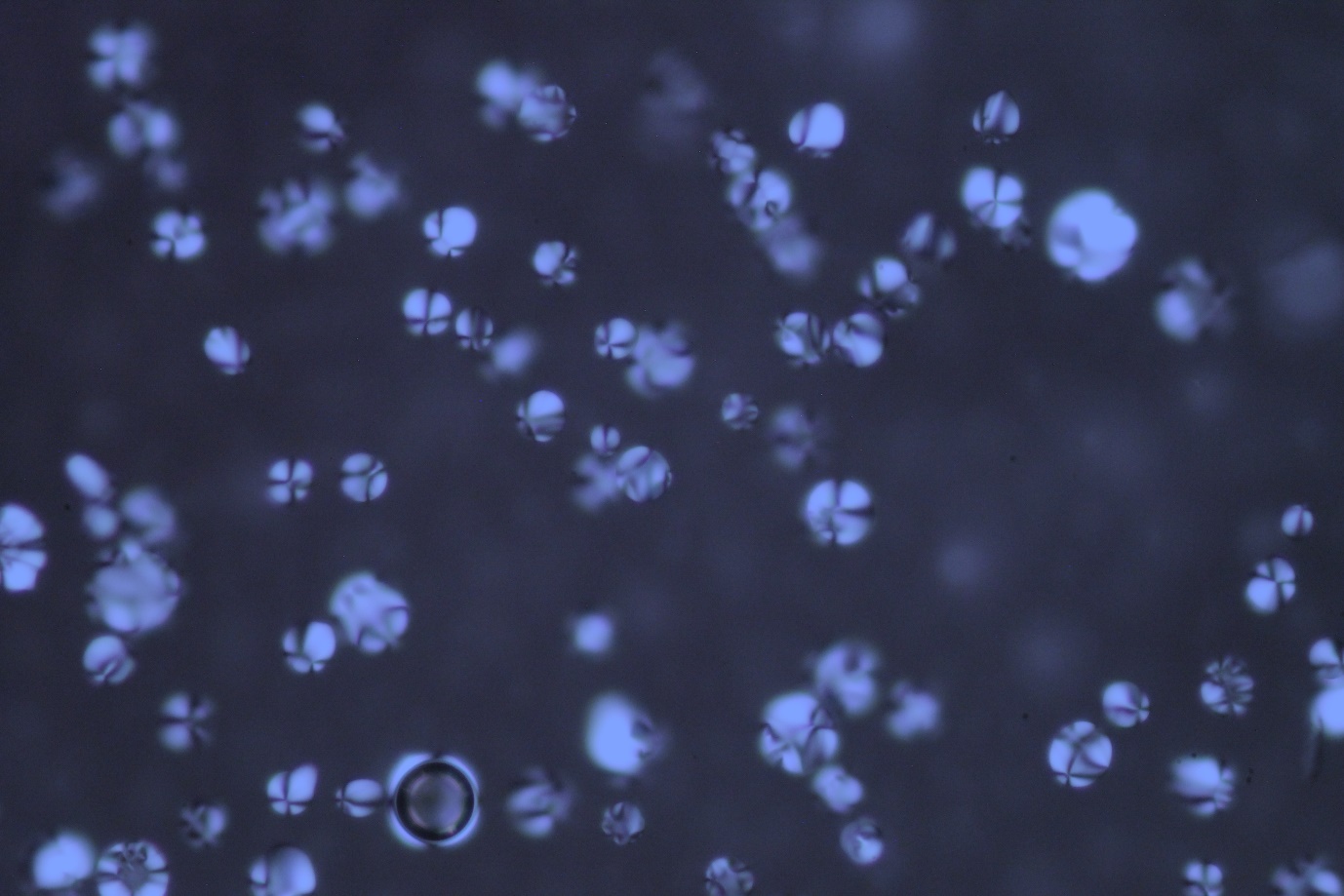


Original Image for Figure 4: (E/L)Gl80, 14 days; middle part of the image is included in the figure.


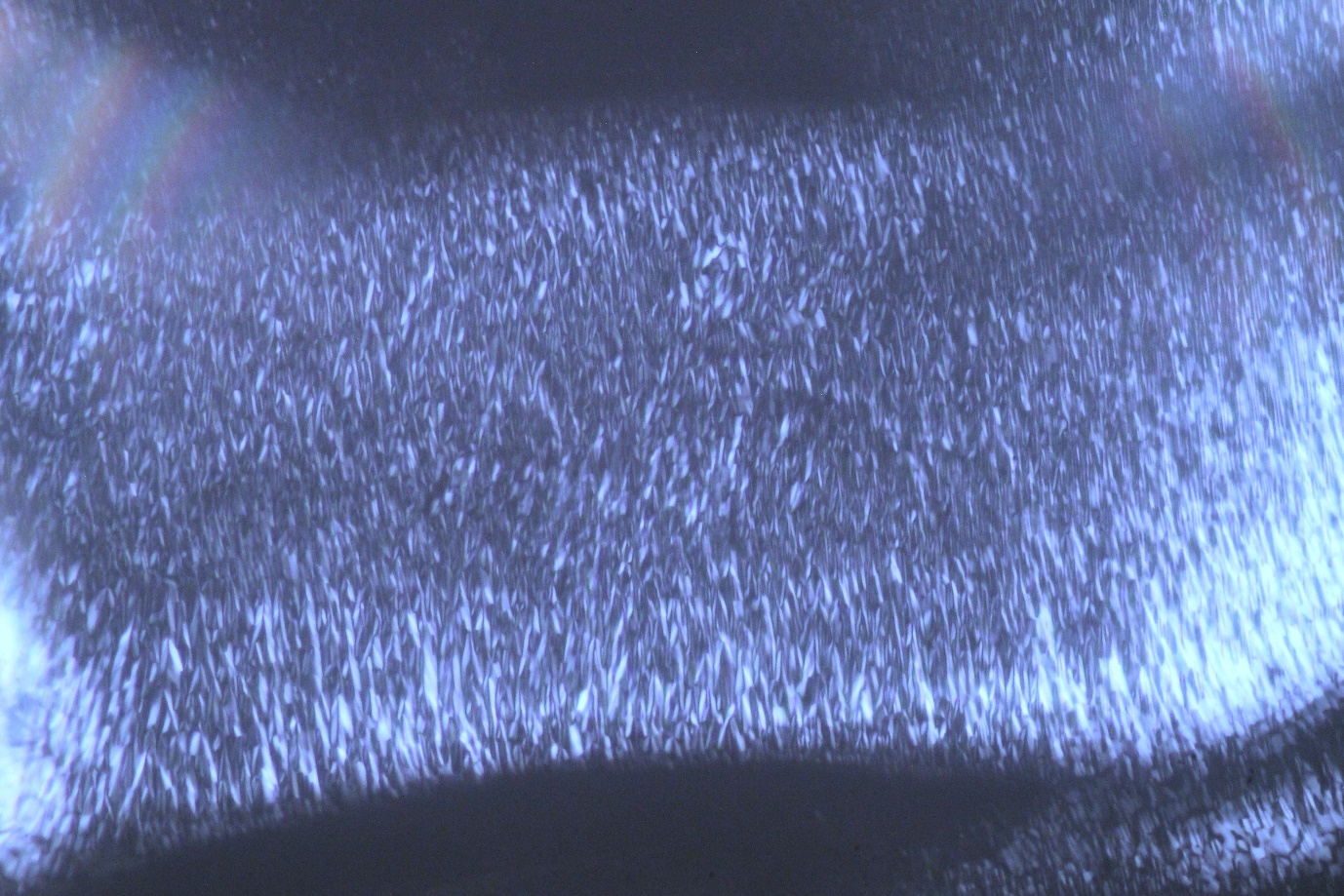


Original Image for Supplementary Figure S2: (E/L)Go50, 6 hours; middle part of the image is included in the figure.


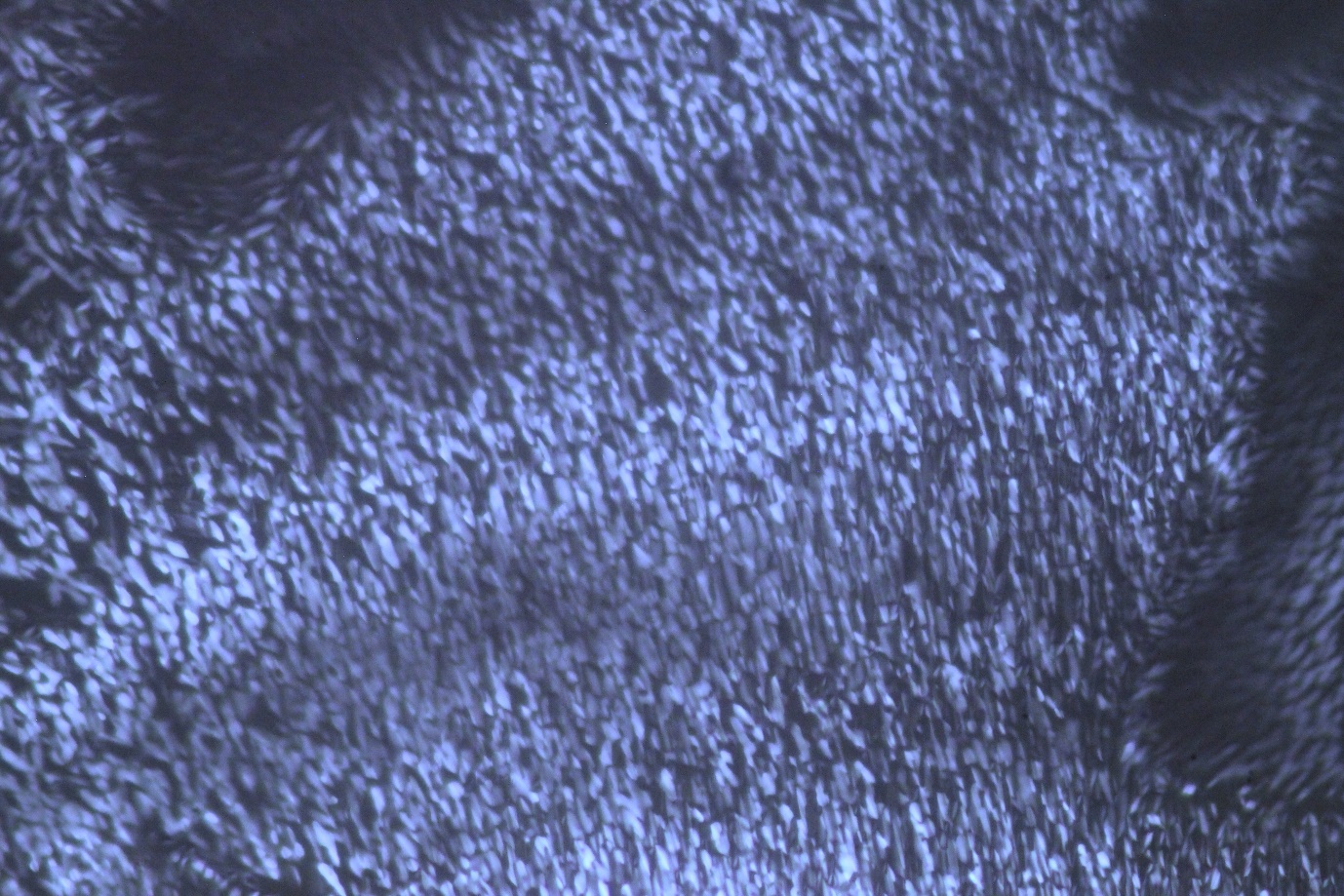


Original Image for Supplementary Figure S2: (E/L)Go50, 12 hours; middle part of the image is included in the figure.


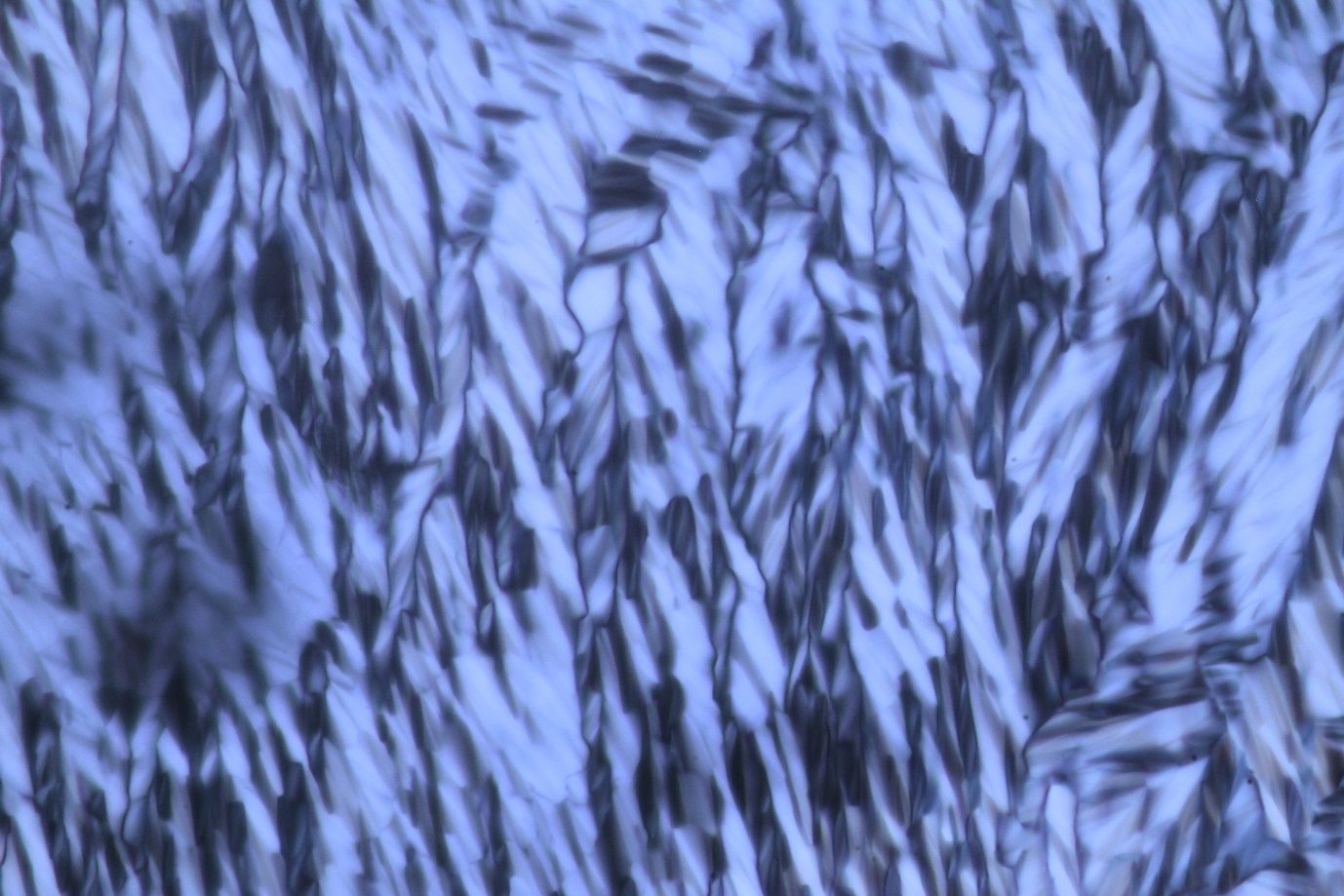


Original Image for Supplementary Figure S2: (E/L)Go50, 36 hours; middle part of the image is included in the figure.


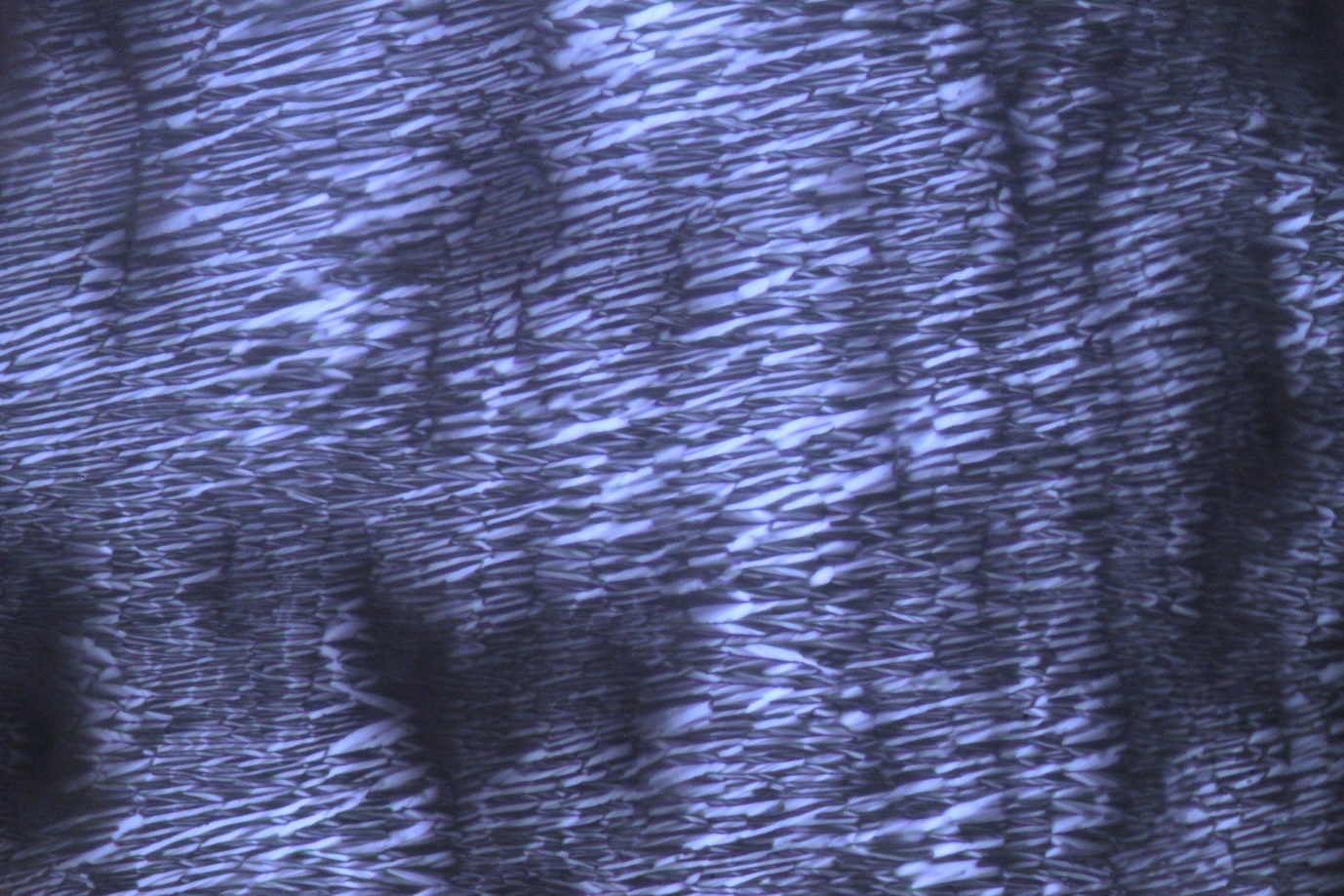


Original Image for Supplementary Figure S2: (E/L)Go50, 48 hours; middle part of the image is included in the figure.


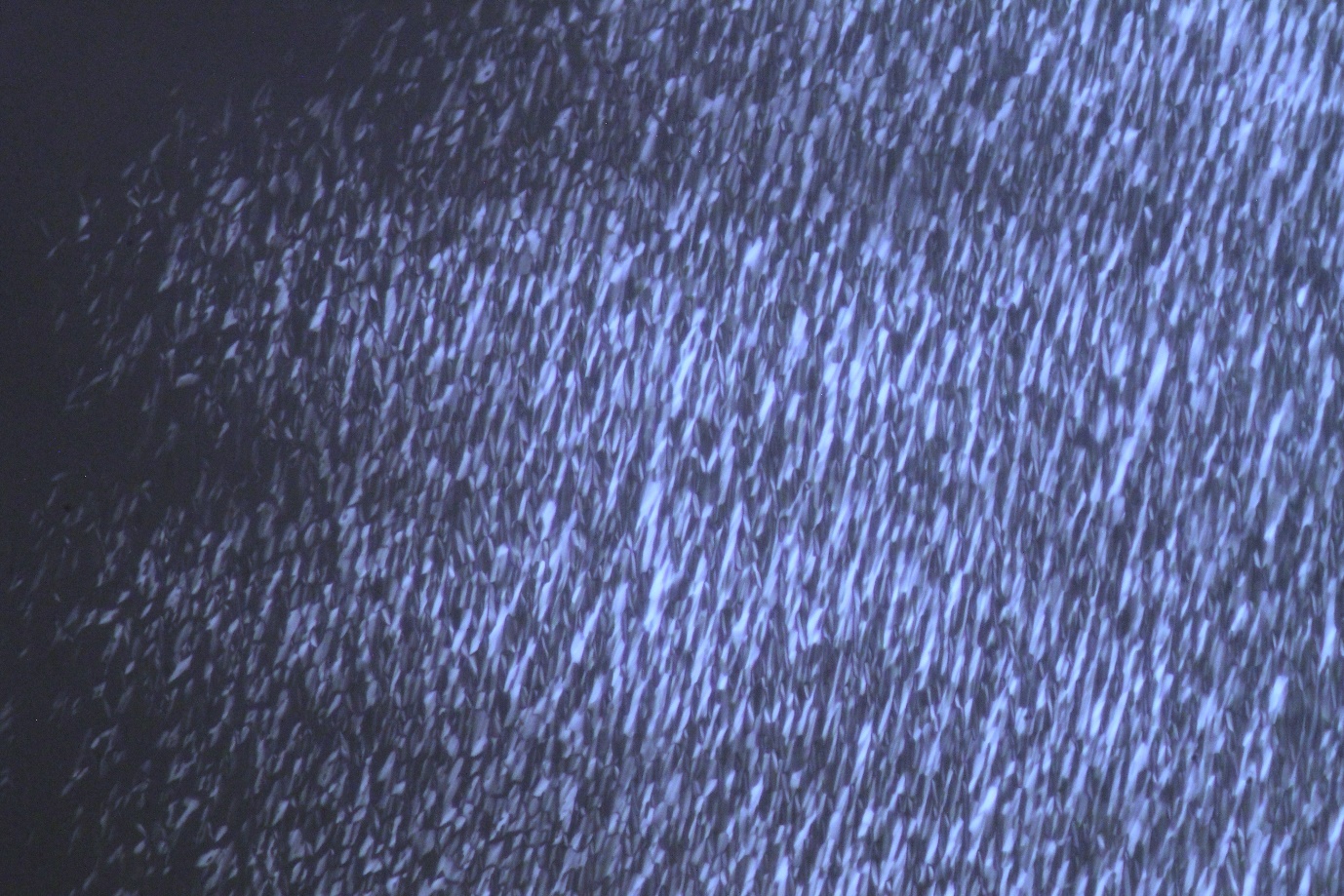


Original Image for Supplementary Figure S2: (E/L)Go50, 10 days; middle part of the image is included in the figure.


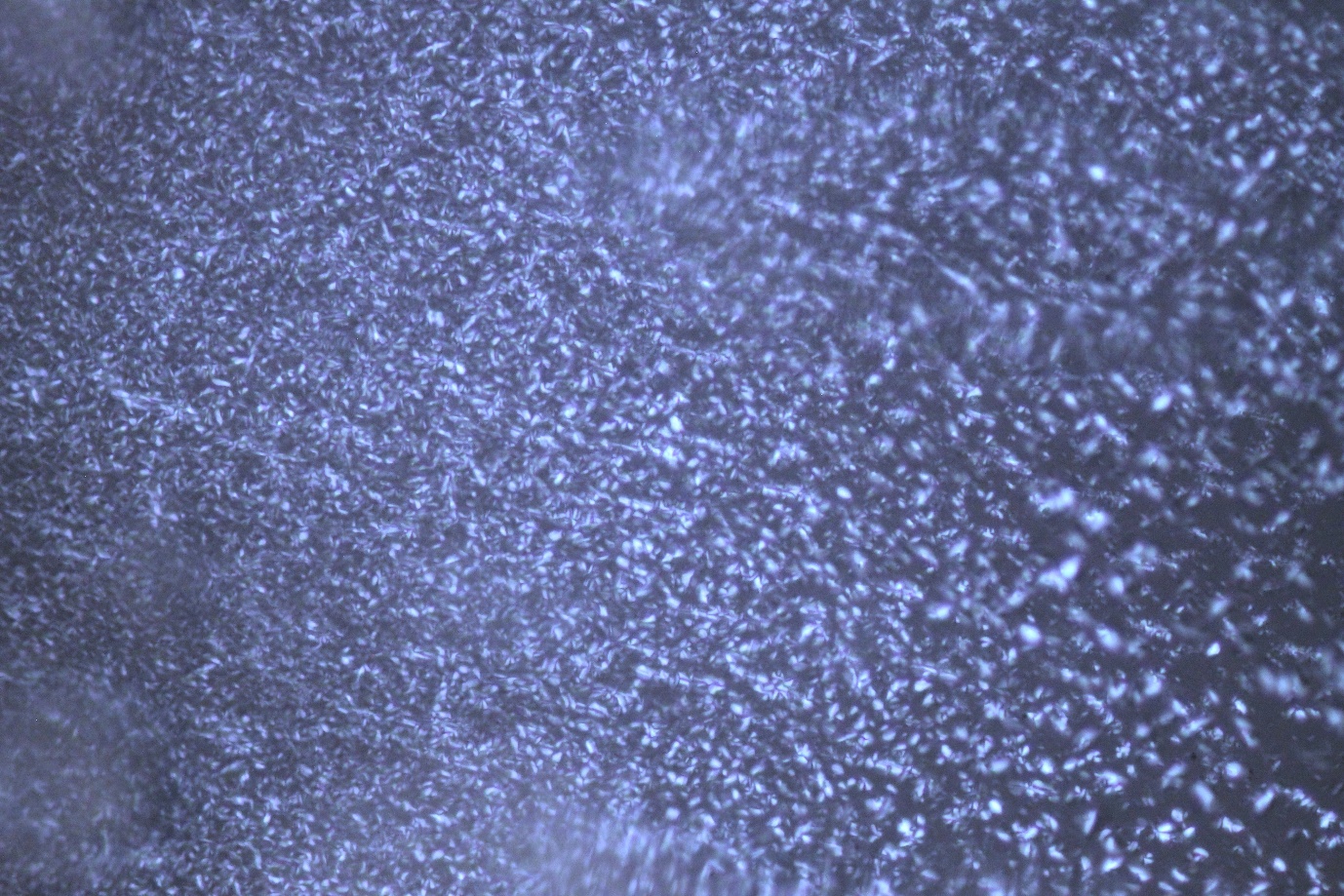


Original Image for Supplementary Figure S2: (E/L)Gl50, 6 hours; middle part of the image is included in the figure.


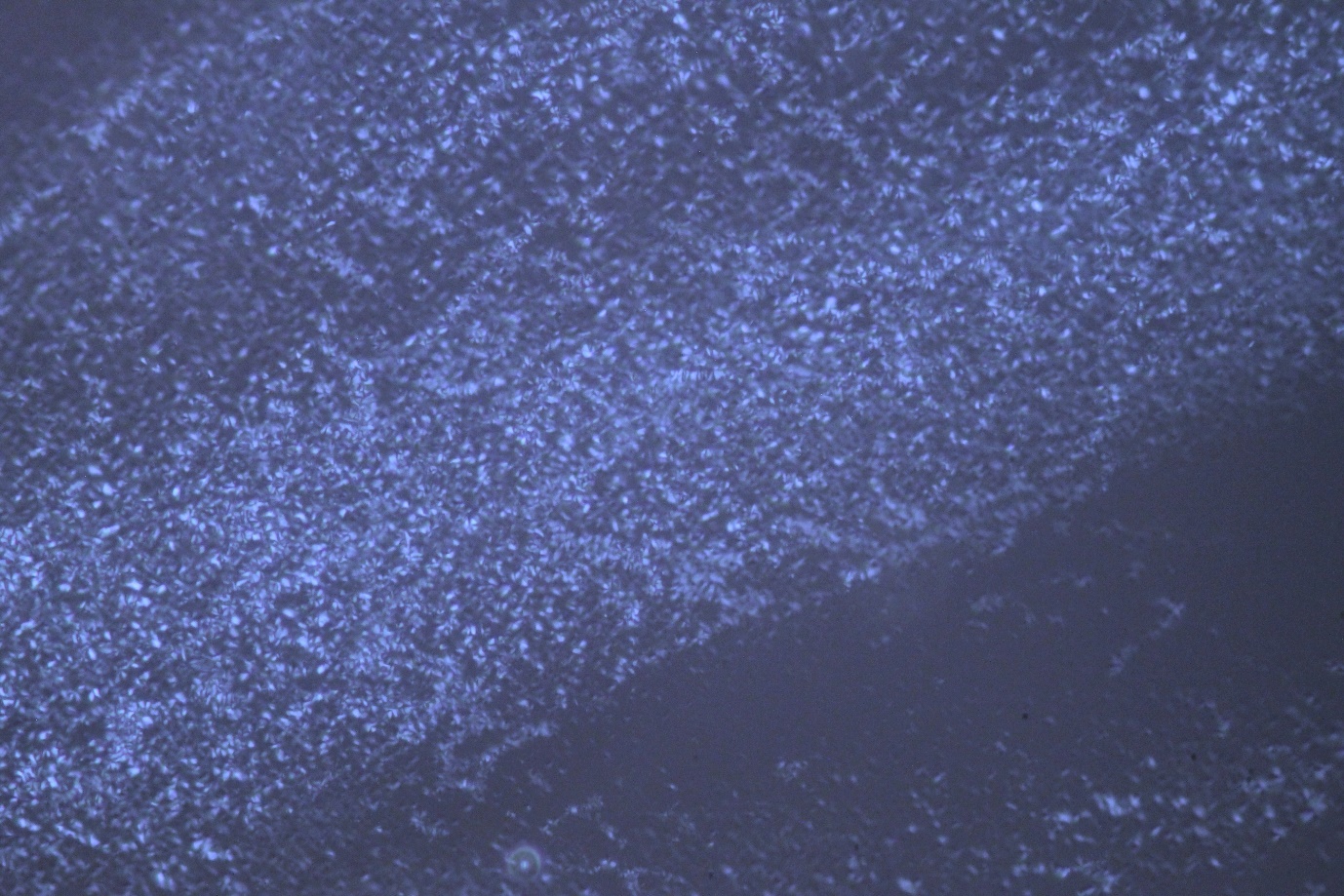


Original Image for Supplementary Figure S2: (E/L)Gl50, 12 hours; middle part of the image is included in the figure.


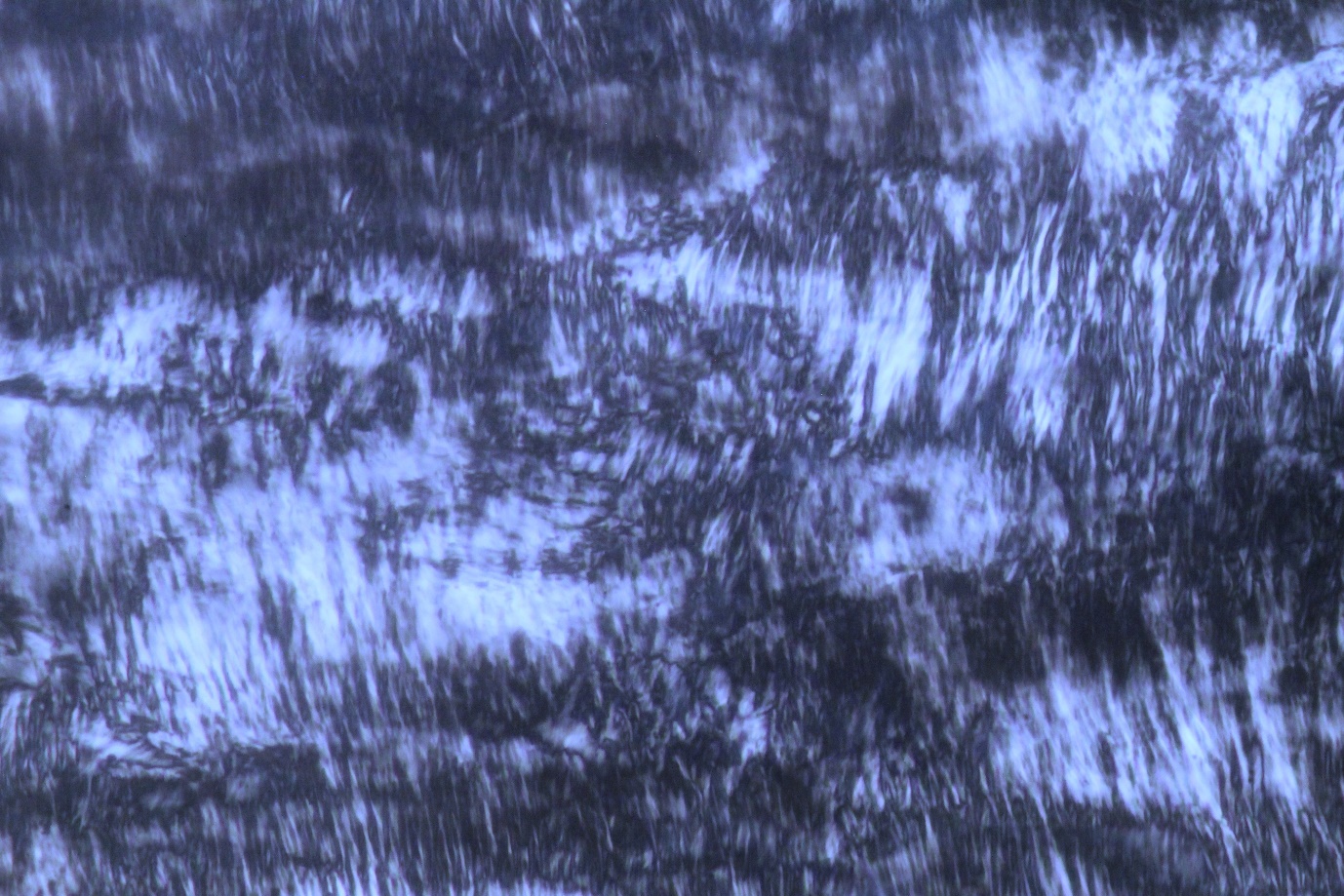


Original Image for Supplementary Figure S2: (E/L)Gl50, 36 hours; middle part of the image is included in the figure.


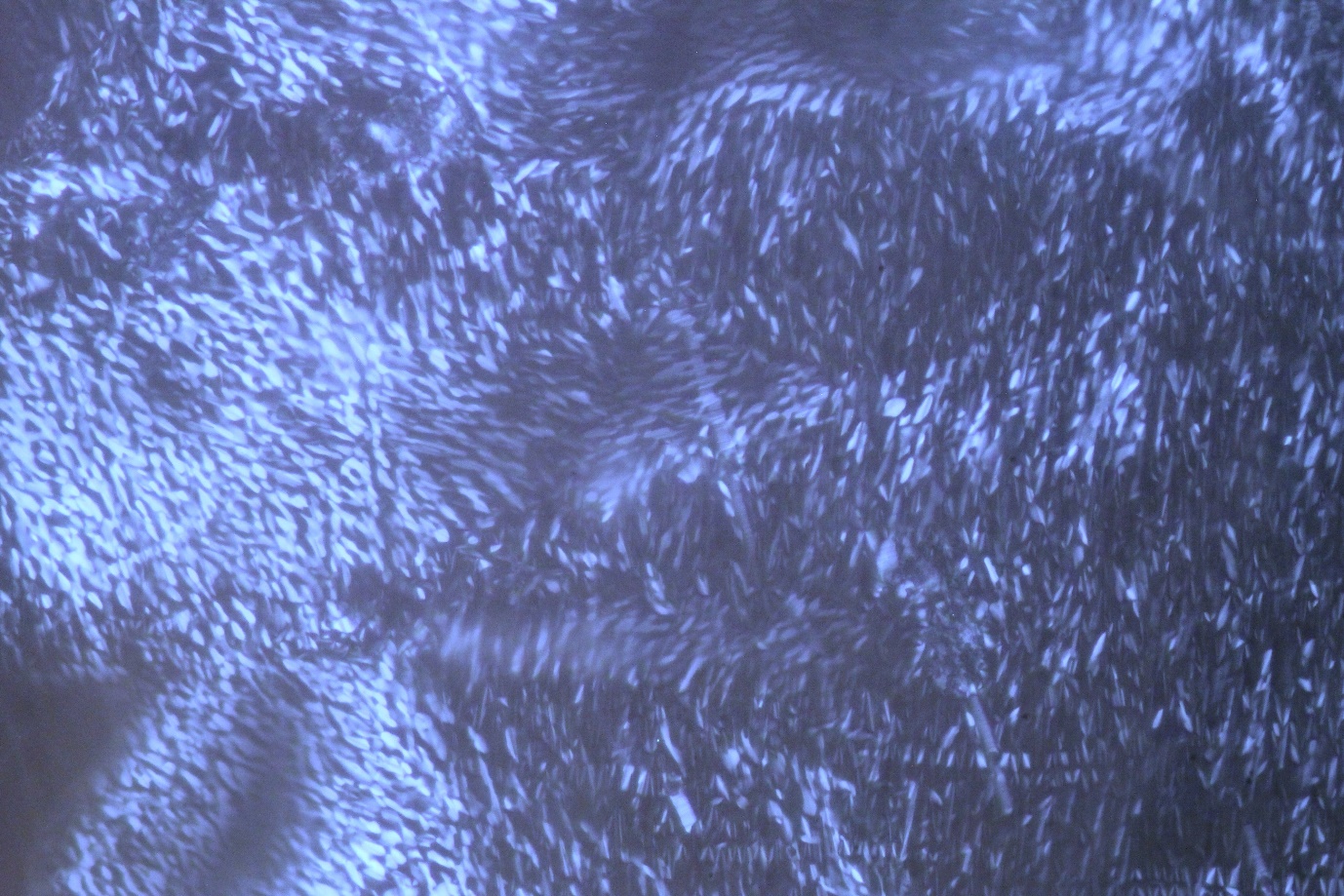


Original Image for Supplementary Figure S2: (E/L)Gl50, 48 hours; middle part of the image is included in the figure.


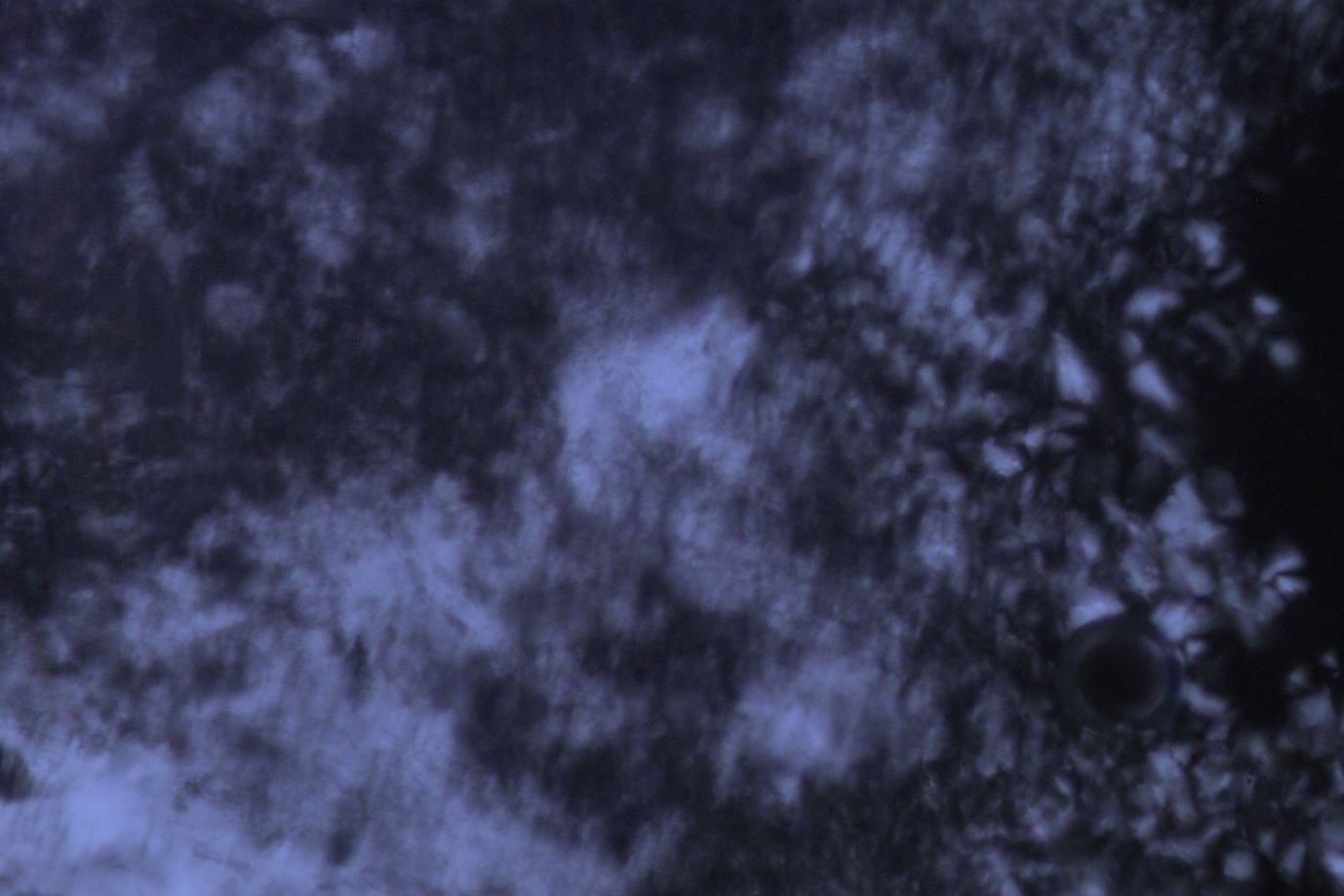


Original Image for Supplementary Figure S2: (E/L)Gl50, 10 days; middle part of the image is included in the figure.


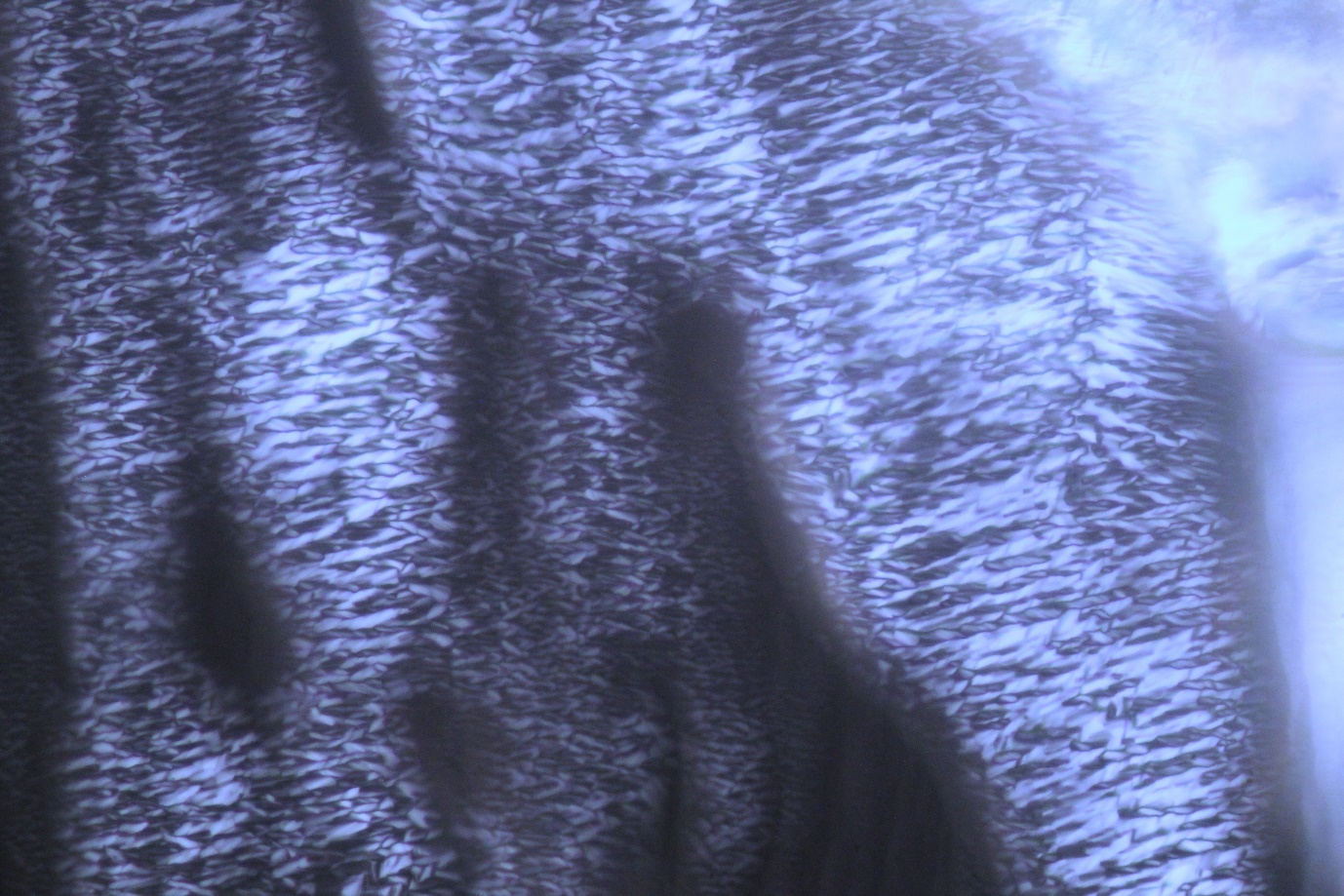


Original Image for Supplementary Figure S2: (E/L)Go60, 6 hours; middle part of the image is included in the figure.


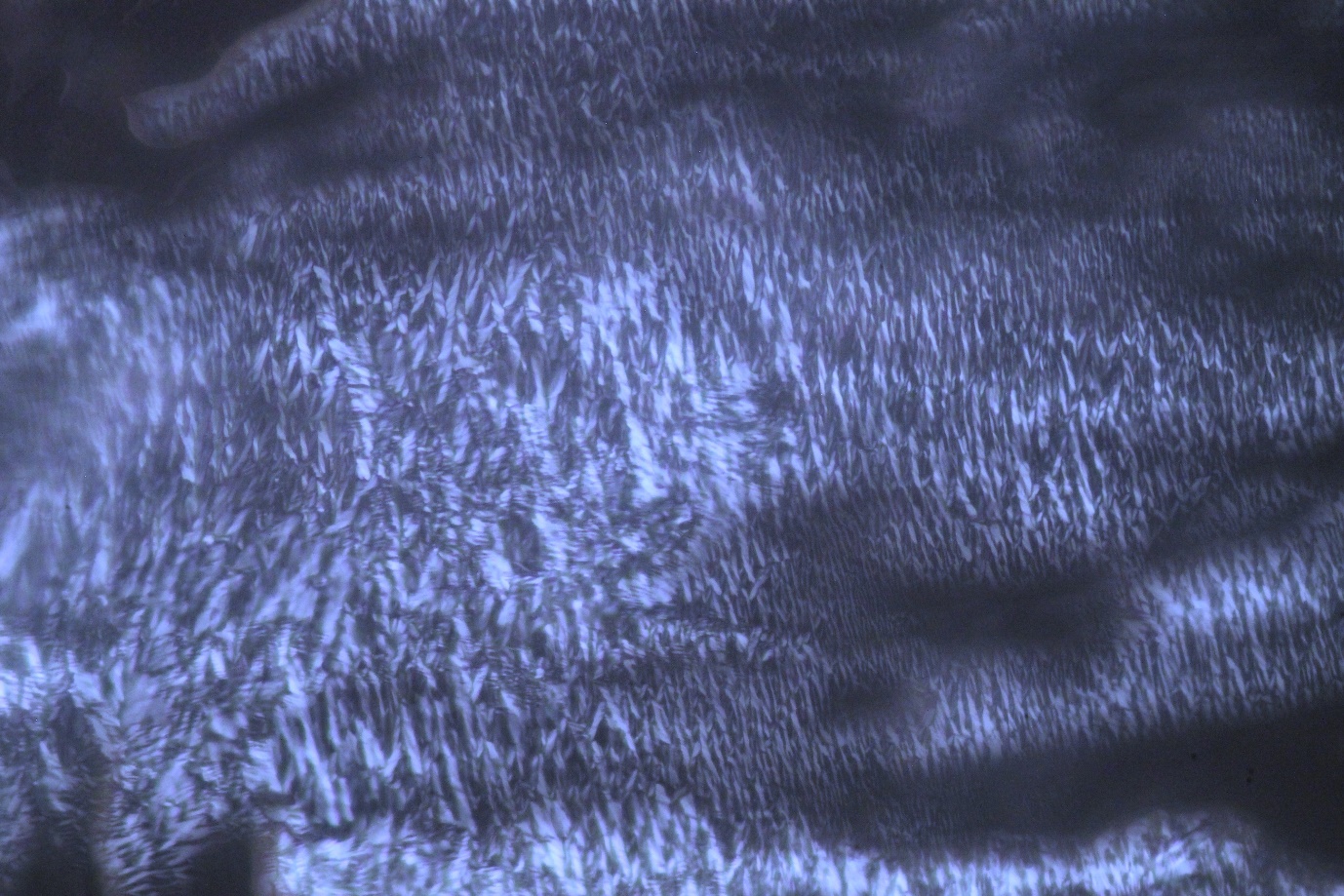


Original Image for Supplementary Figure S2: (E/L)Go60, 12 hours; middle part of the image is included in the figure.


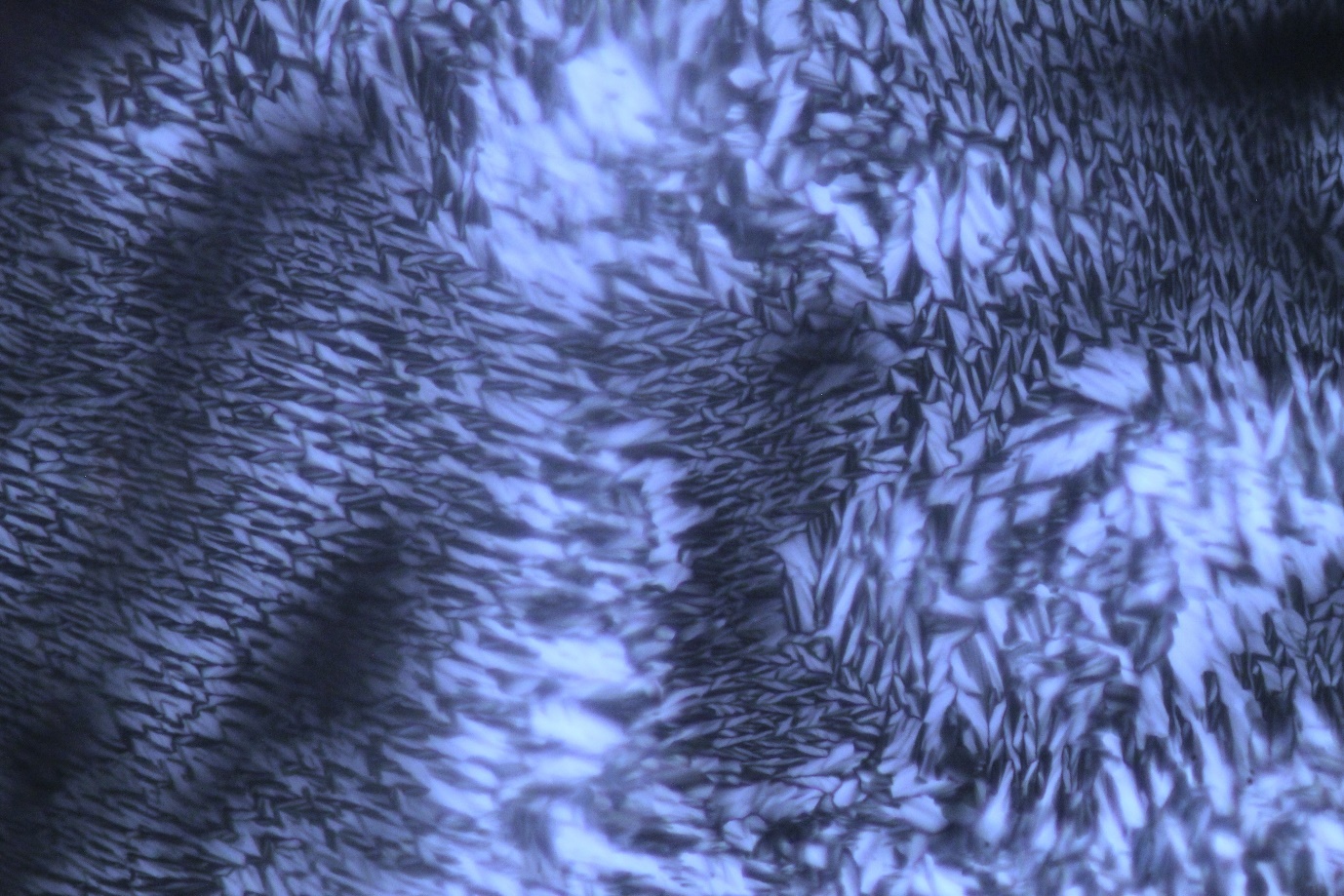


Original Image for Supplementary Figure S2: (E/L)Go60, 36 hours; middle part of the image is included in the figure.


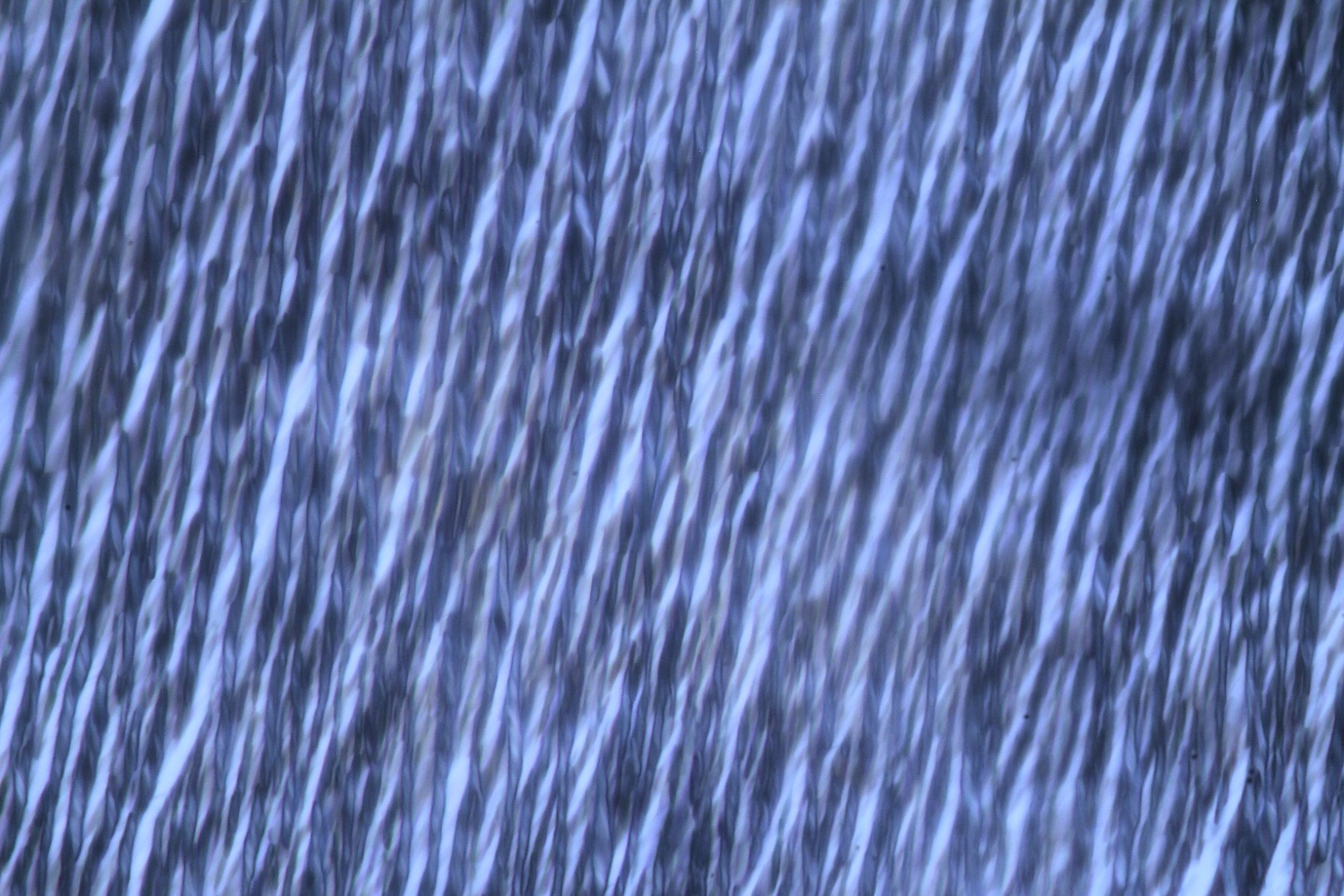


Original Image for Supplementary Figure S2: (E/L)Go60, 48 hours; middle part of the image is included in the figure.


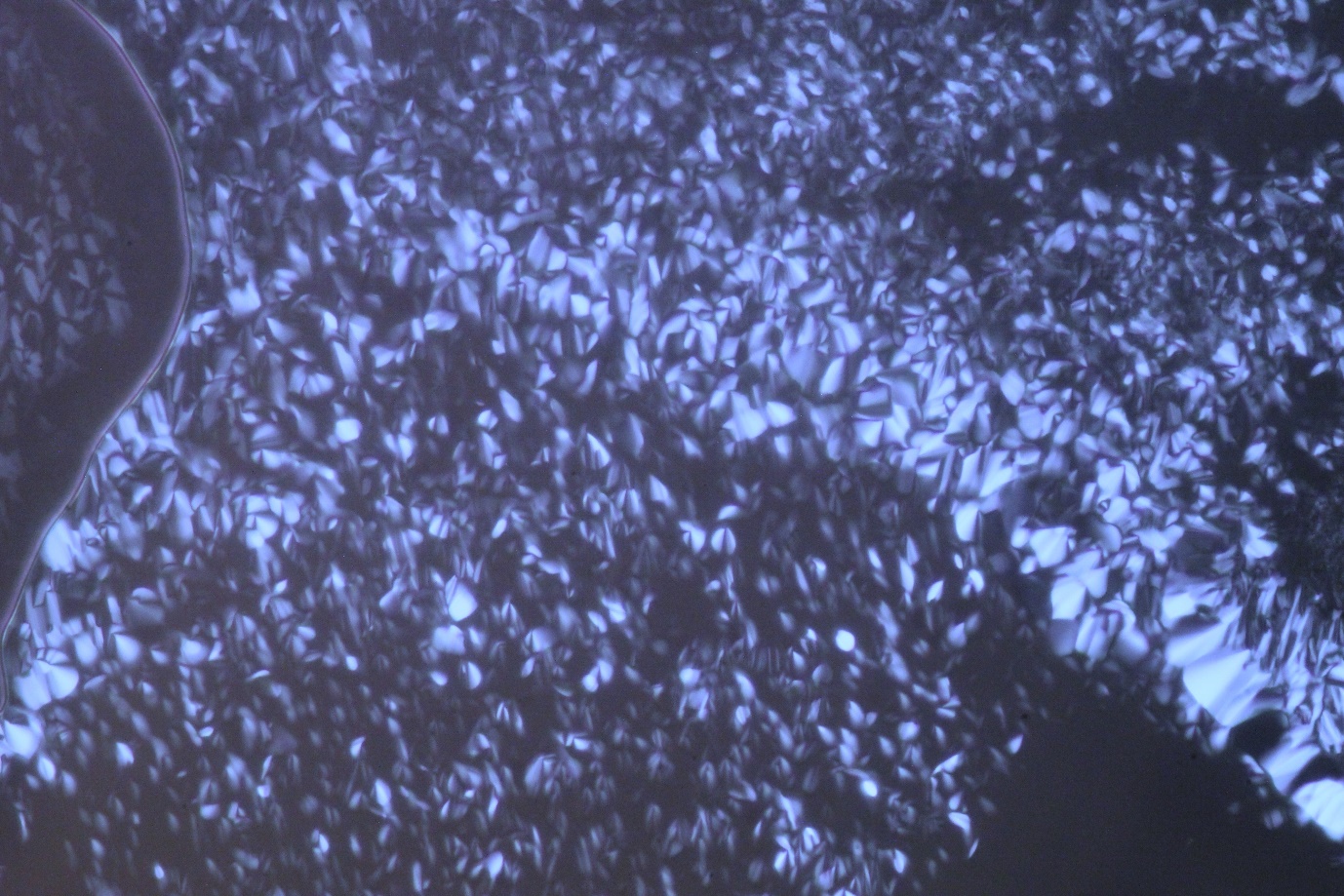


Original Image for Supplementary Figure S2: (E/L)Go60, 10 days; middle part of the image is included in the figure.


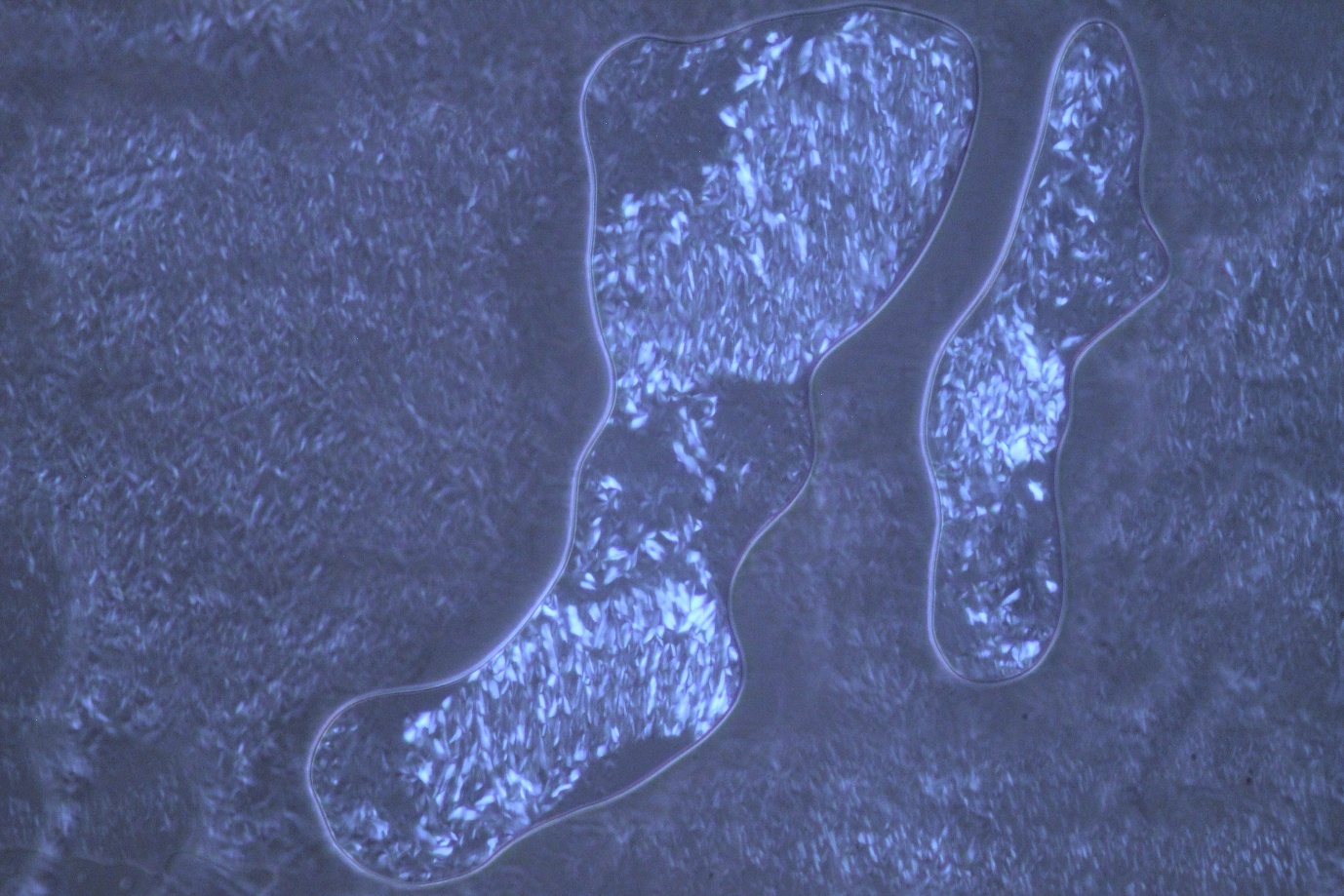


Original Image for Supplementary Figure S2: (E/L)Gl60, 6 hours; middle part of the image is included in the figure.


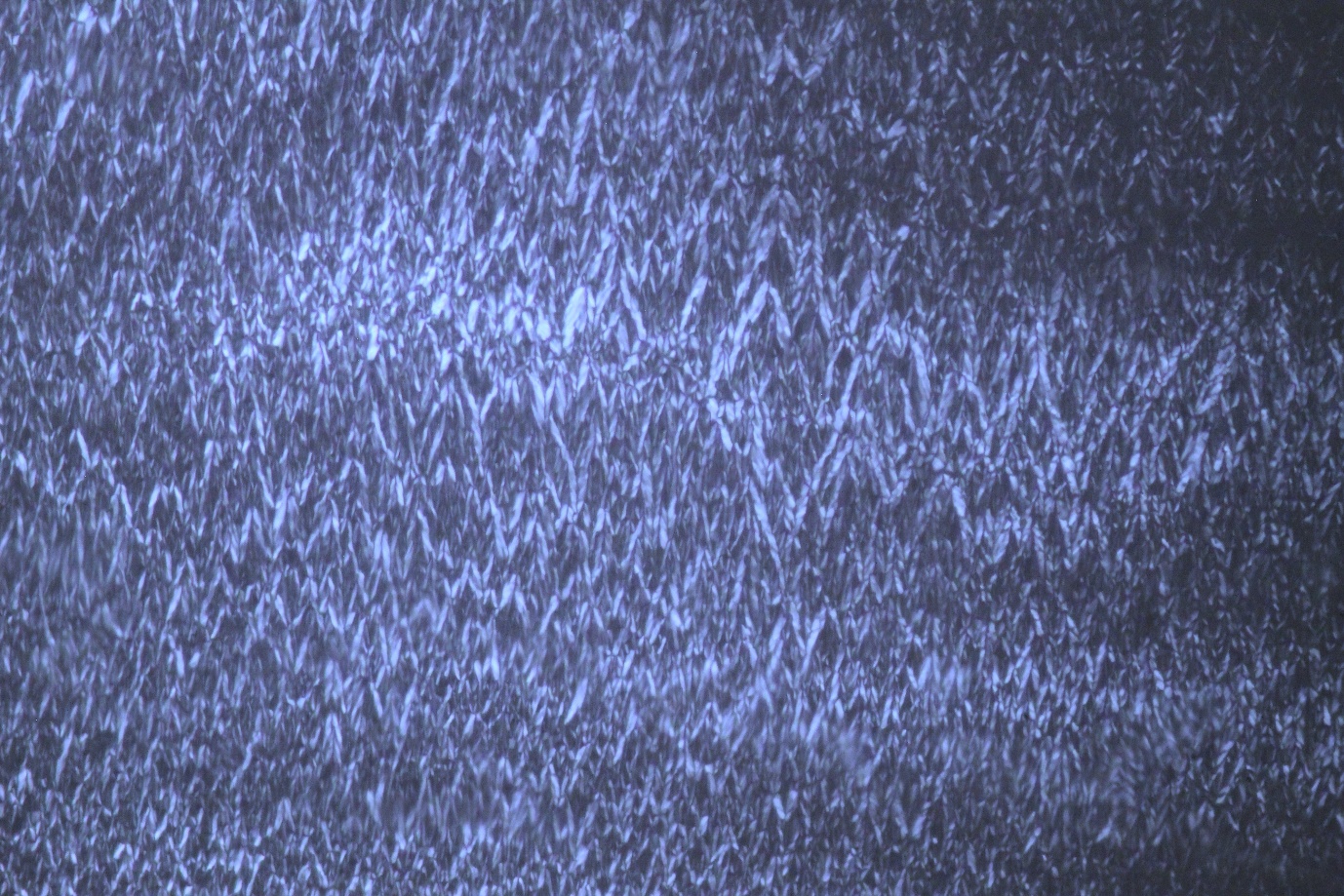


Original Image for Supplementary Figure S2: (E/L)Gl60, 12 hours; middle part of the image is included in the figure.


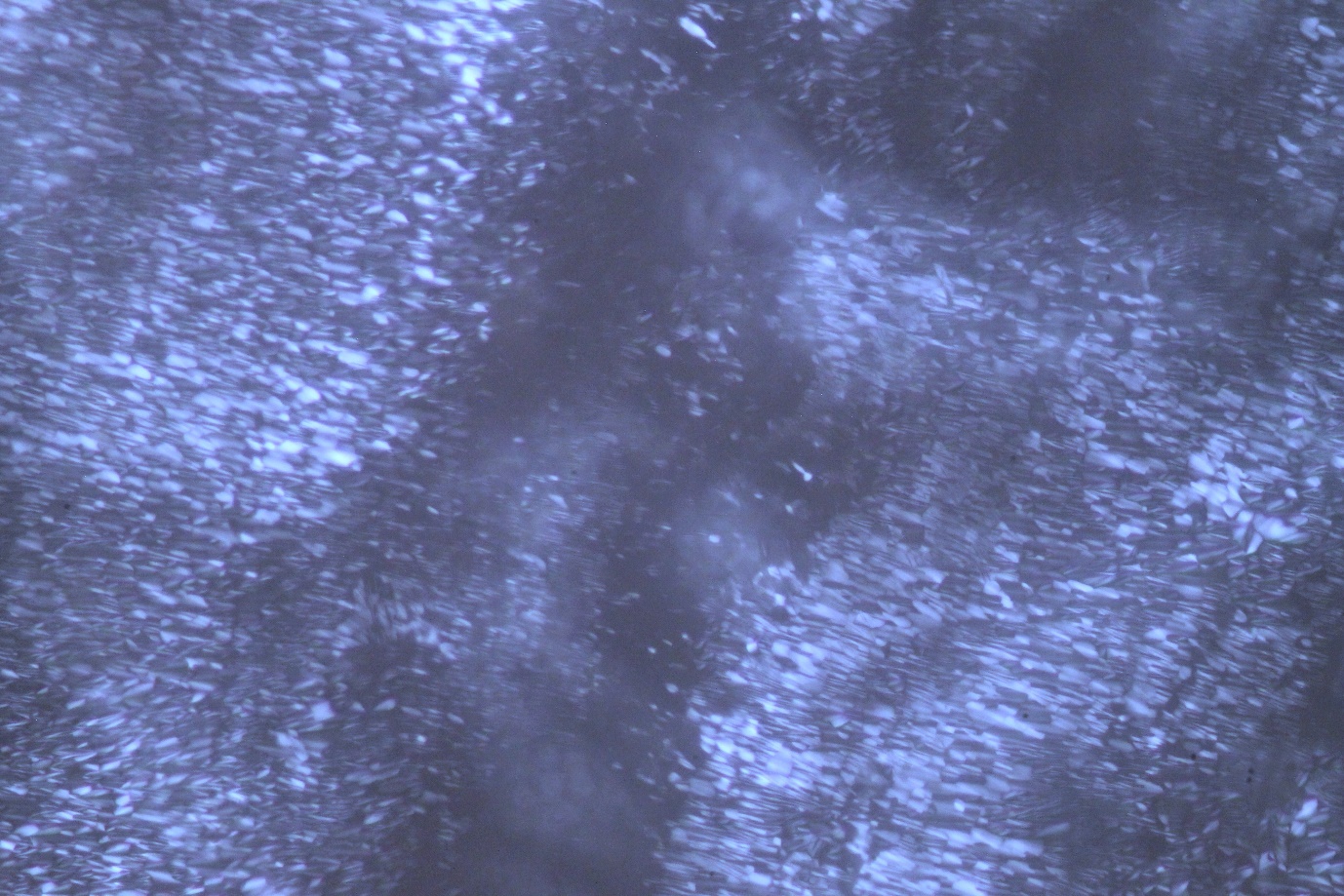


Original Image for Supplementary Figure S2: (E/L)Gl60, 36 hours; middle part of the image is included in the figure.


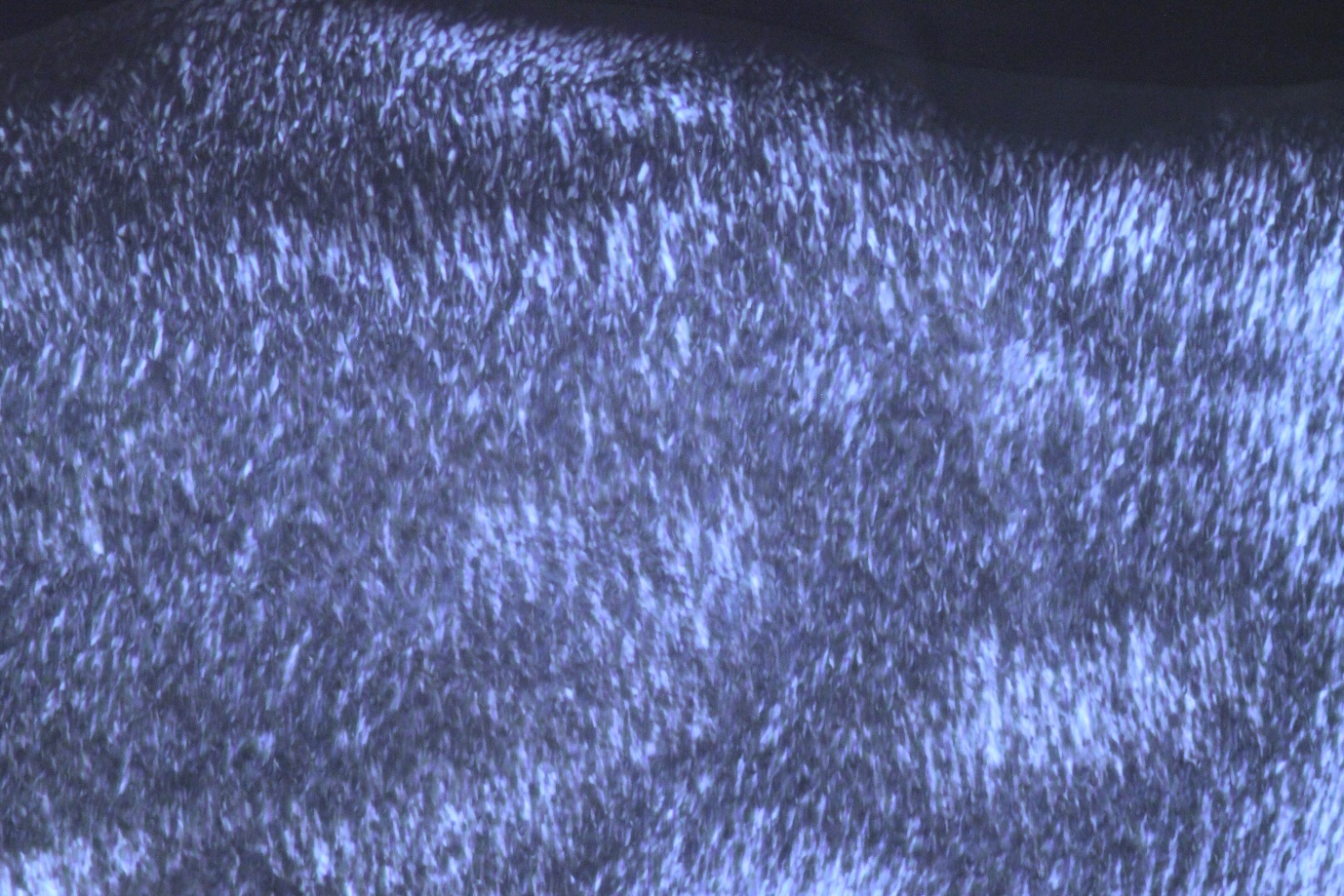


Original Image for Supplementary Figure S2: (E/L)Gl60, 48 hours; middle part of the image is included in the figure.


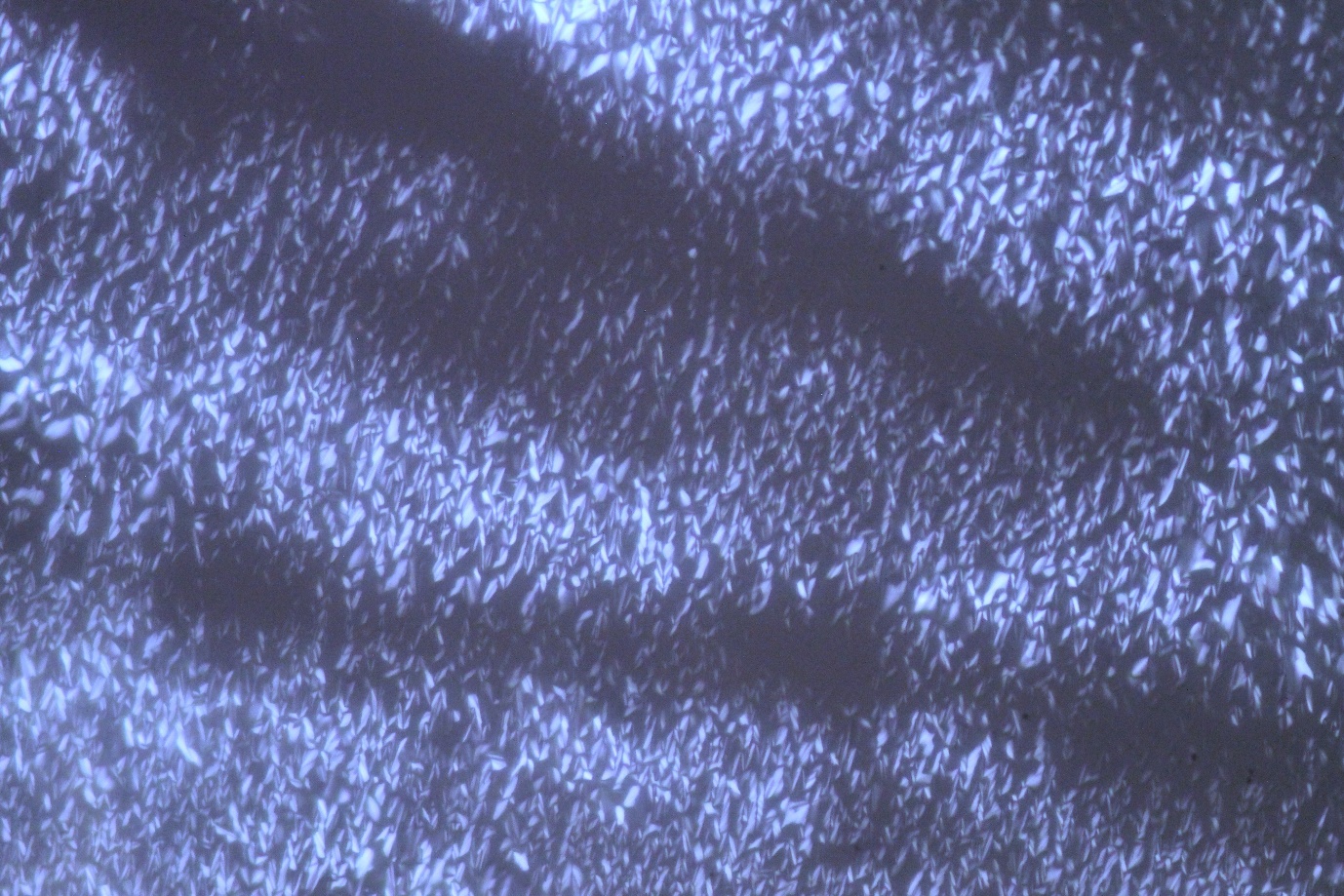


Original Image for Supplementary Figure S2: (E/L)Gl60, 10 days; middle part of the image is included in the figure.


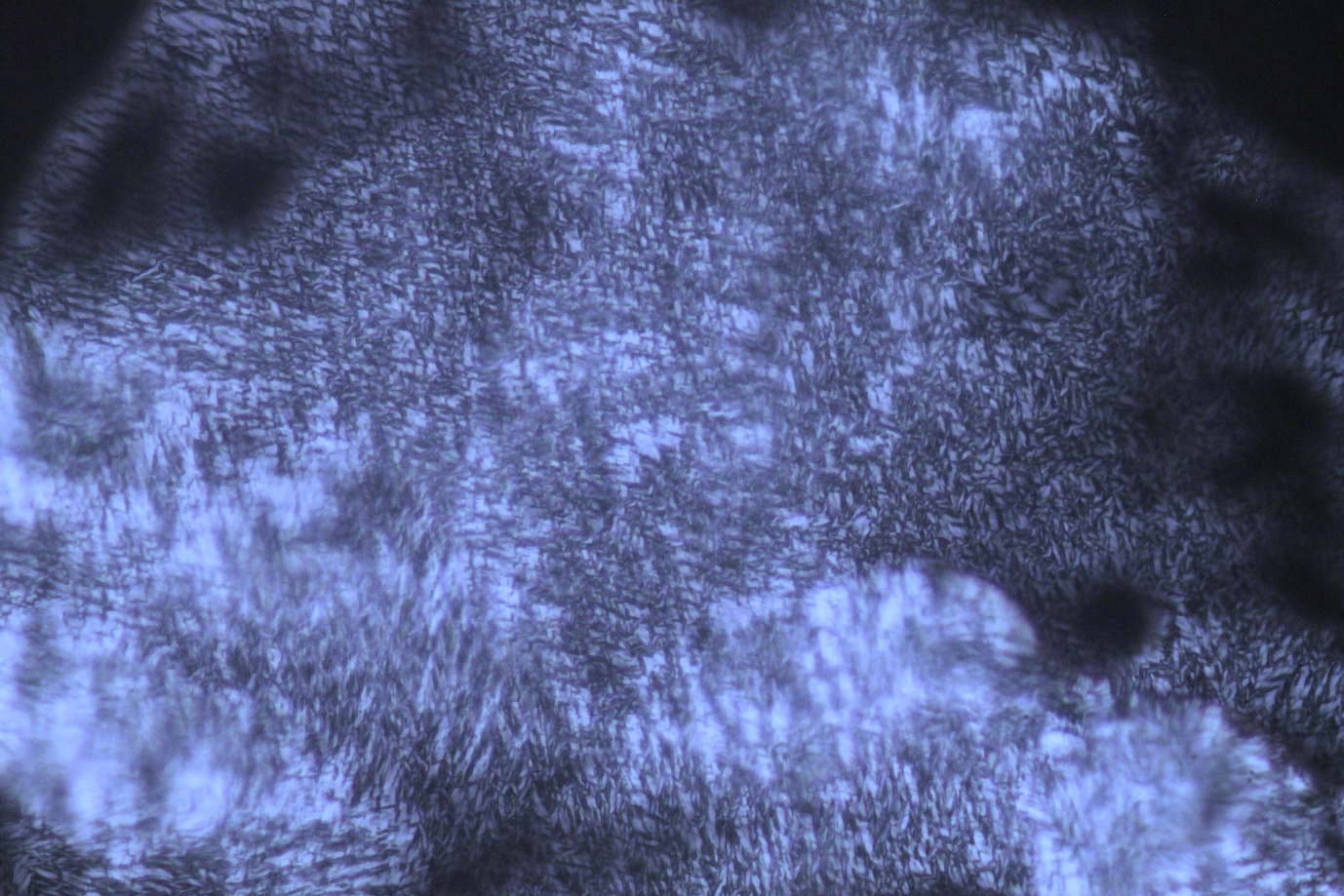


Original Image for Supplementary Figure S2: (E/L)Go70, 6 hours; middle part of the image is included in the figure.


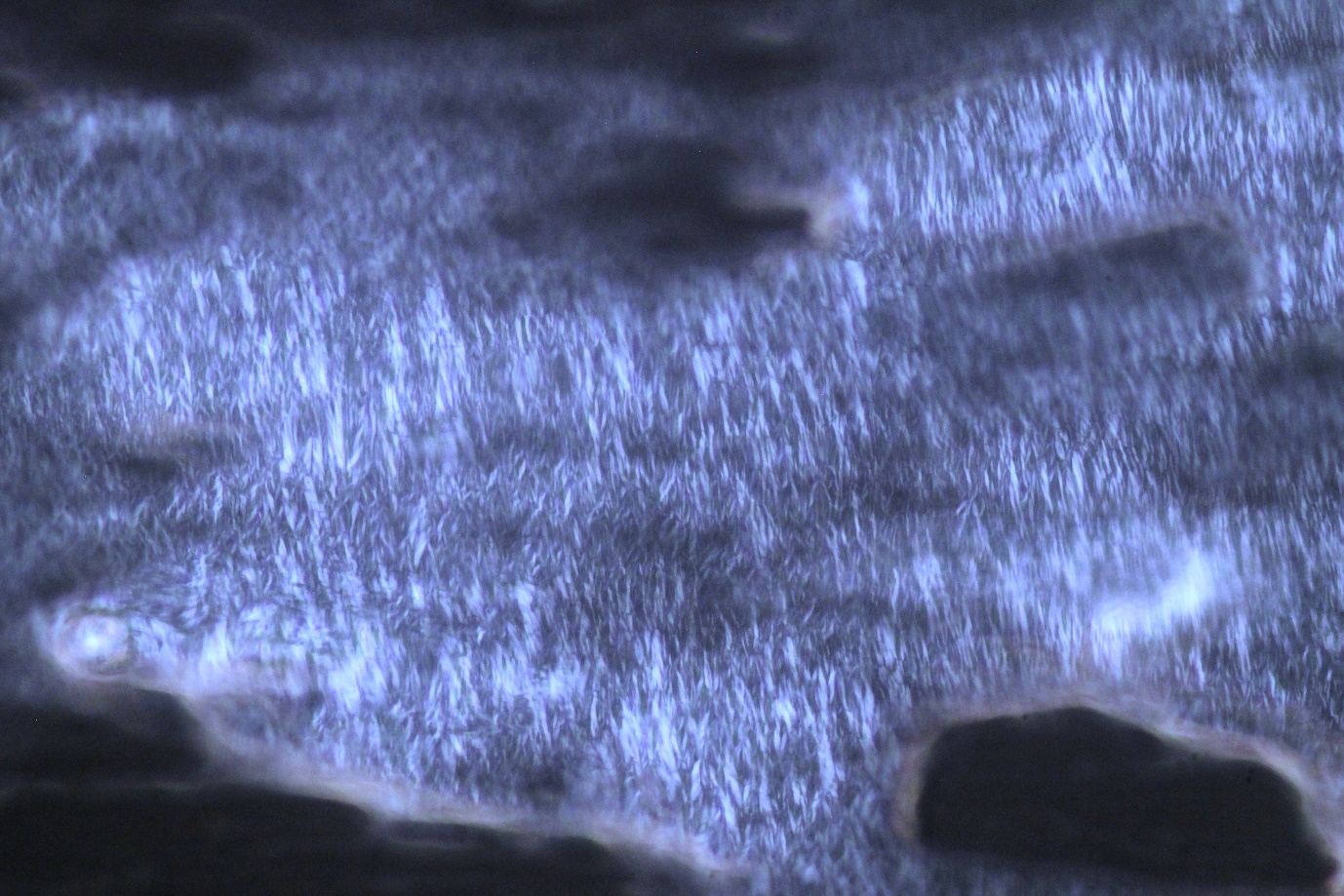


Original Image for Supplementary Figure S2: (E/L)Go70, 12 hours; middle part of the image is included in the figure.


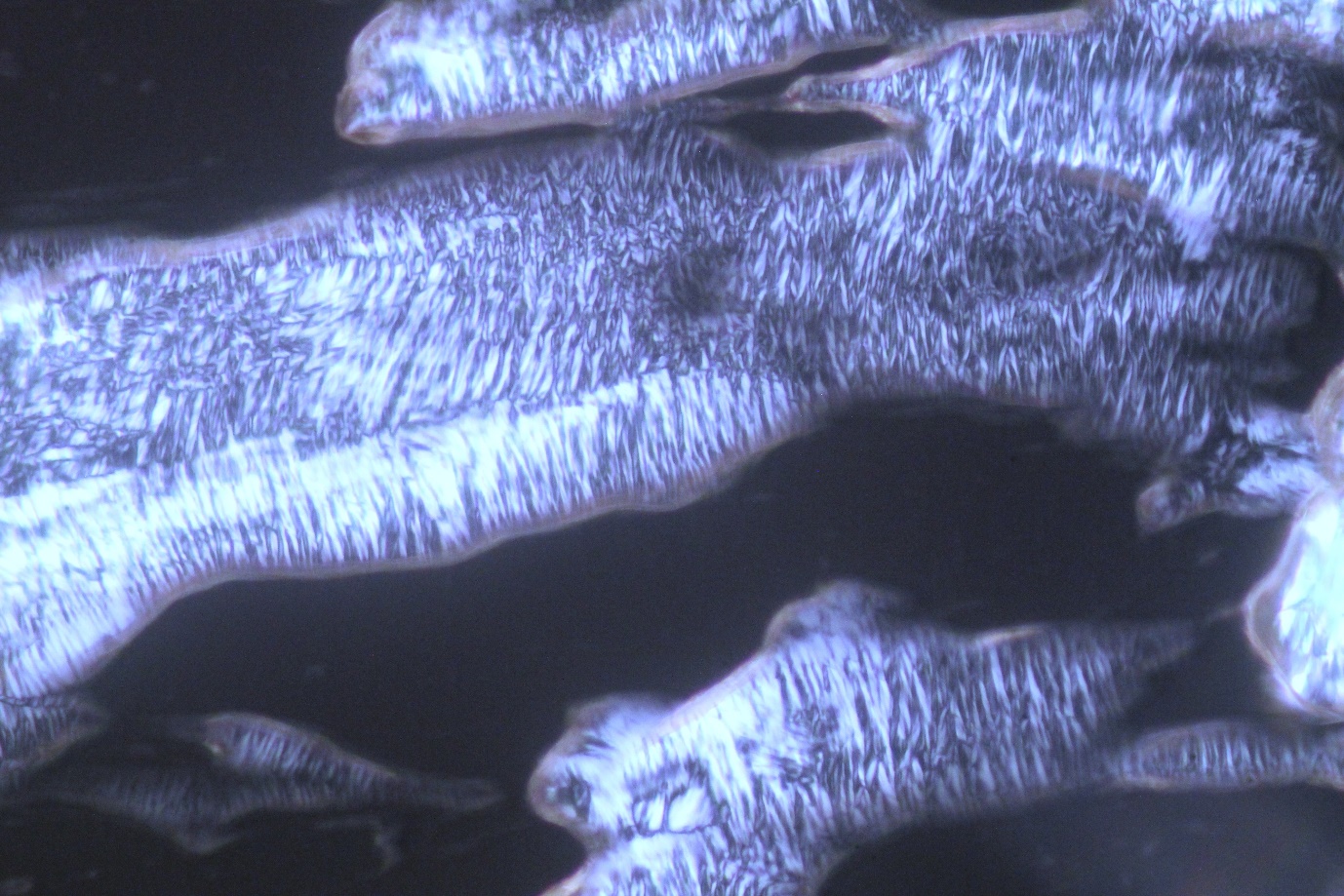


Original Image for Supplementary Figure S2: (E/L)Go70, 36 hours; middle part of the image is included in the figure.


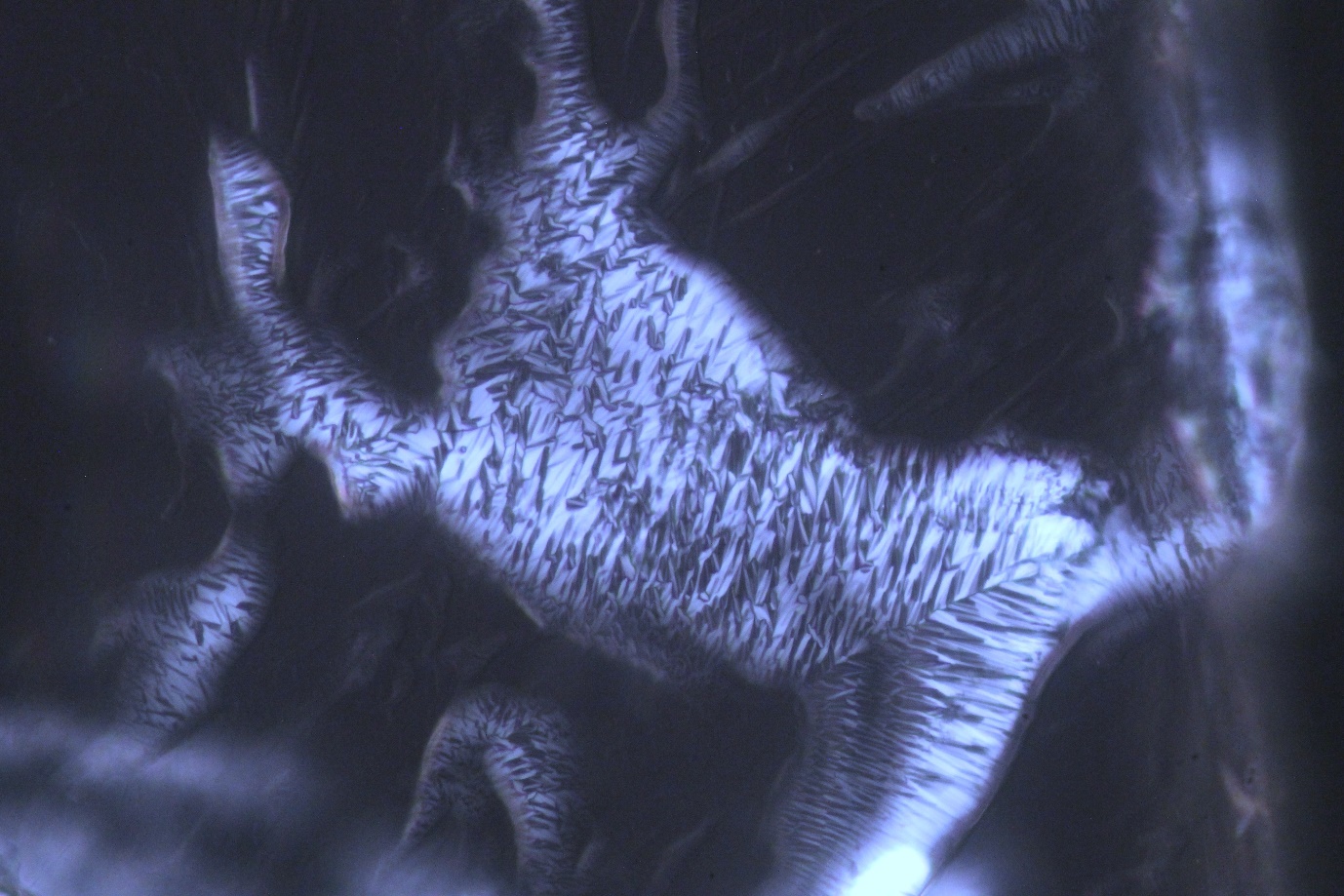


Original Image for Supplementary Figure S2: (E/L)Go70, 48 hours; middle part of the image is included in the figure.


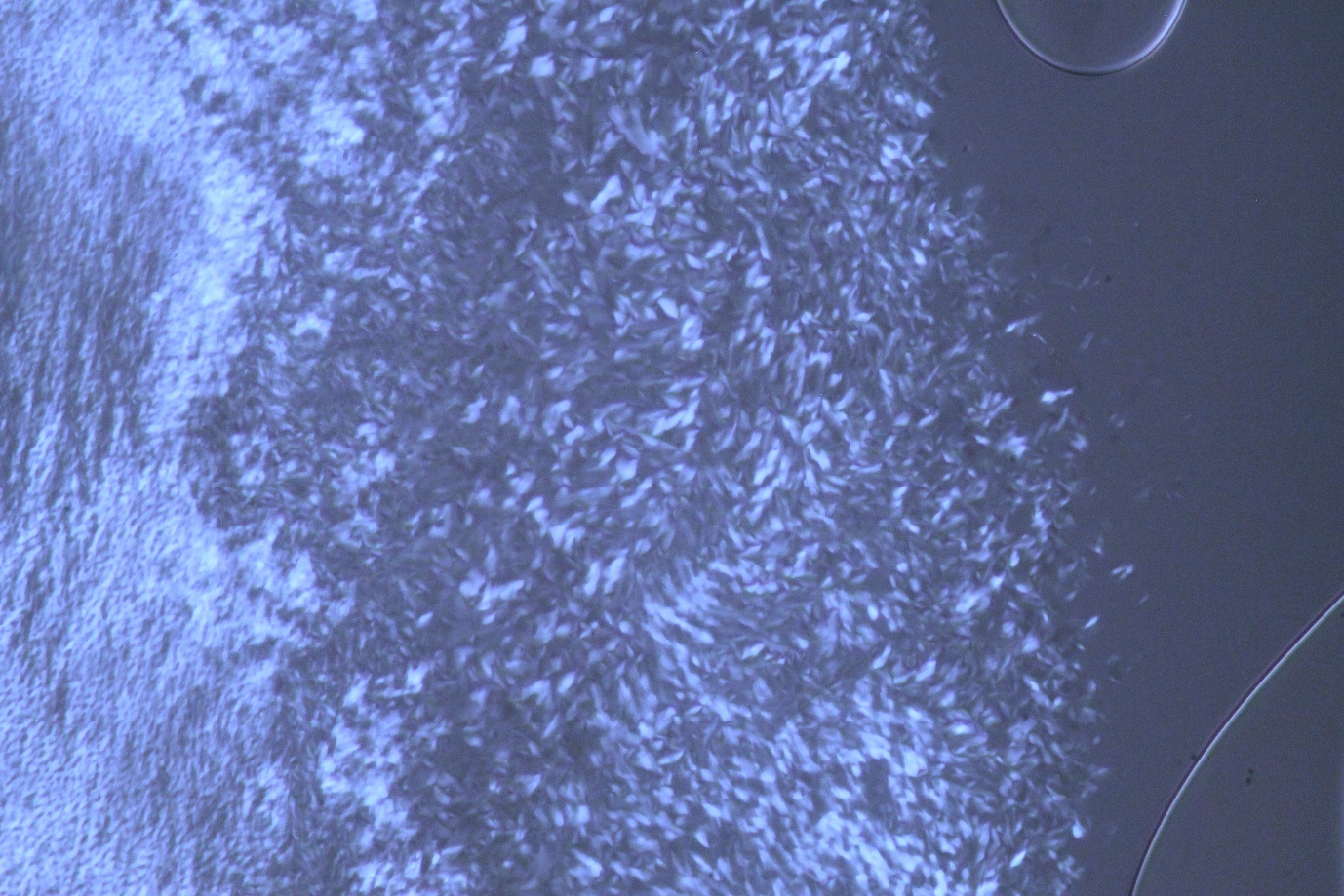


Original Image for Supplementary Figure S2: (E/L)Go70, 10 days; middle part of the image is included in the figure.


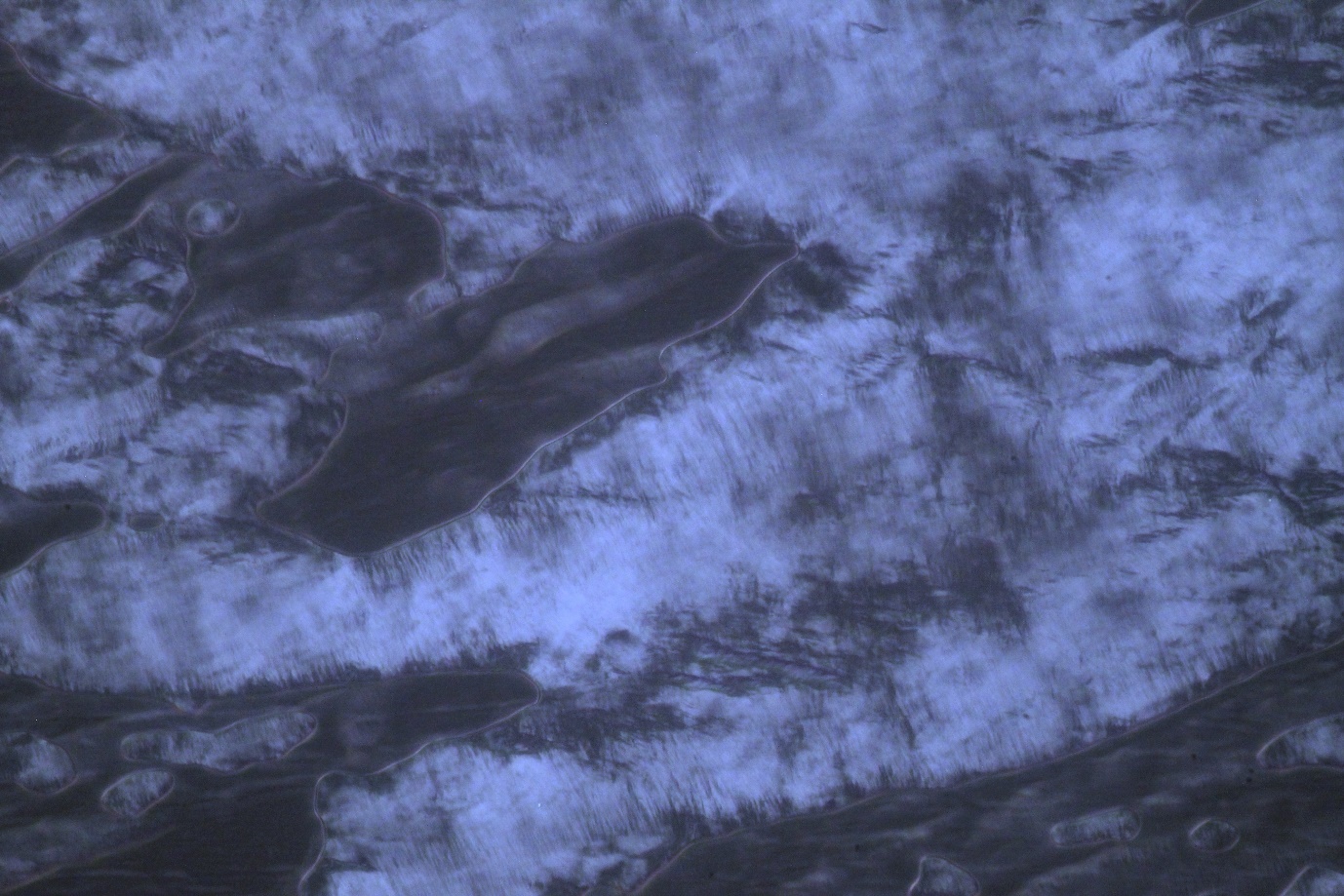


Original Image for Supplementary Figure S2: (E/L)Gl70, 6 hours; middle part of the image is included in the figure.


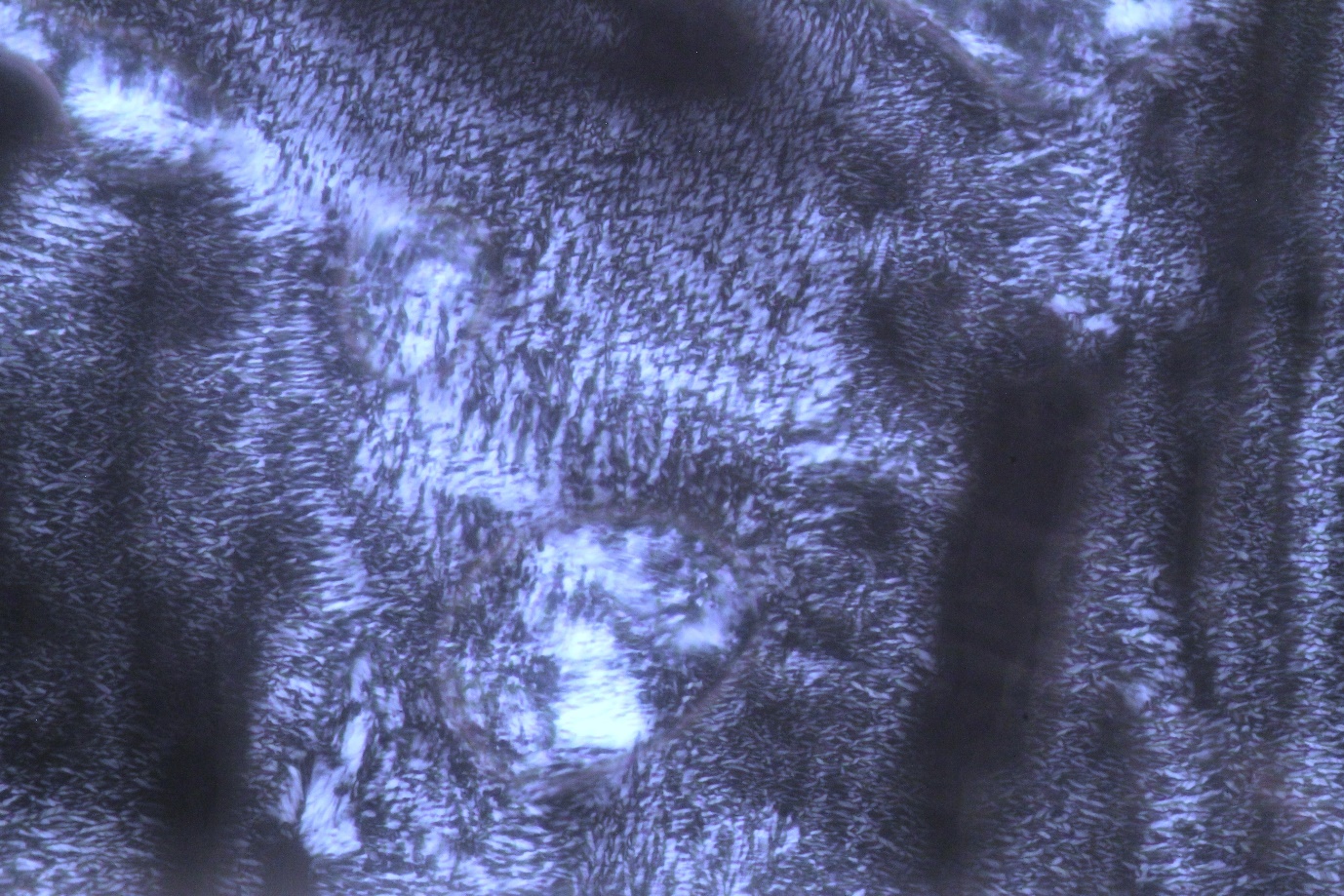


Original Image for Supplementary Figure S2: (E/L)Gl70, 12 hours; middle part of the image is included in the figure.


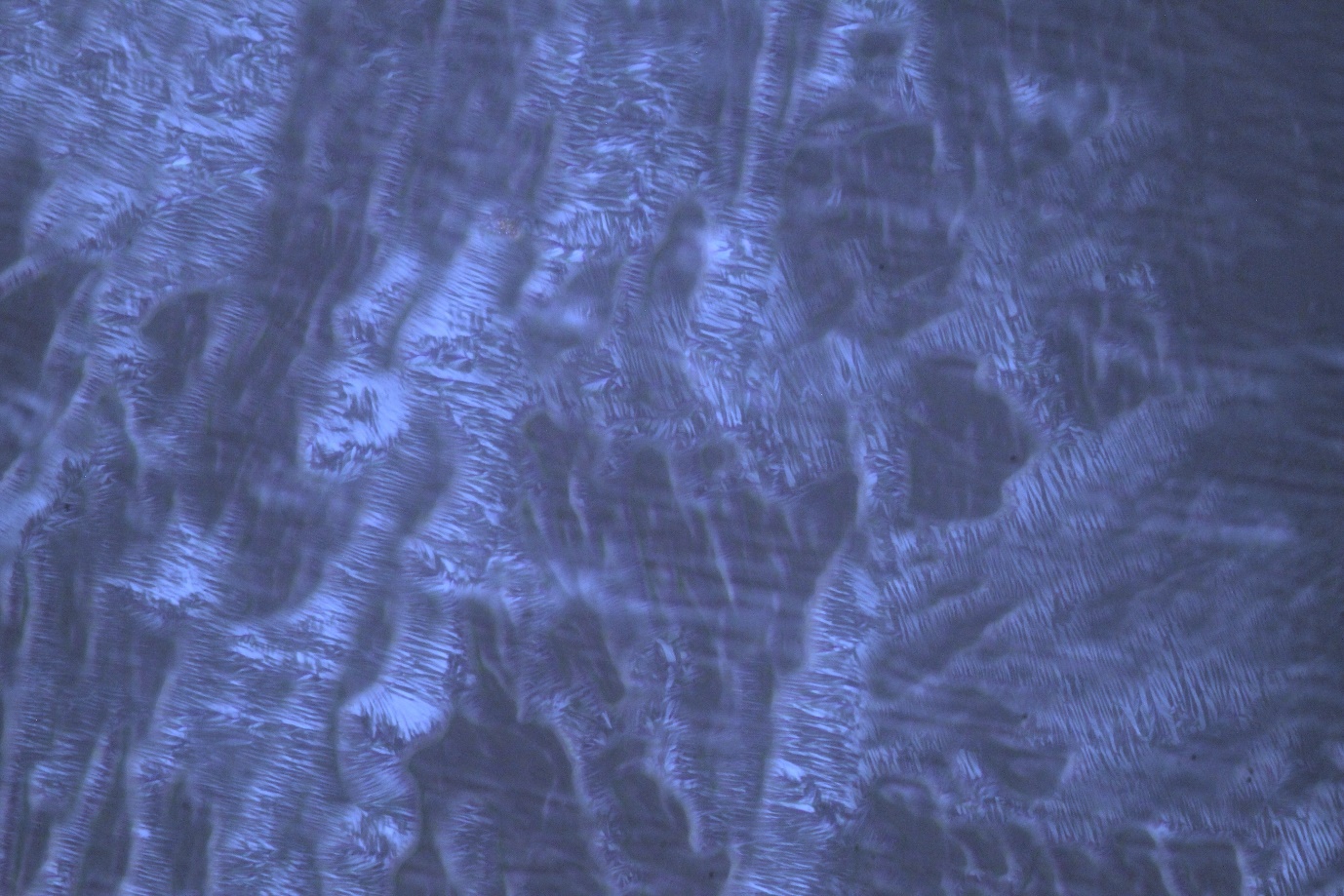


Original Image for Supplementary Figure S2: (E/L)Gl70, 36 hours; middle part of the image is included in the figure.


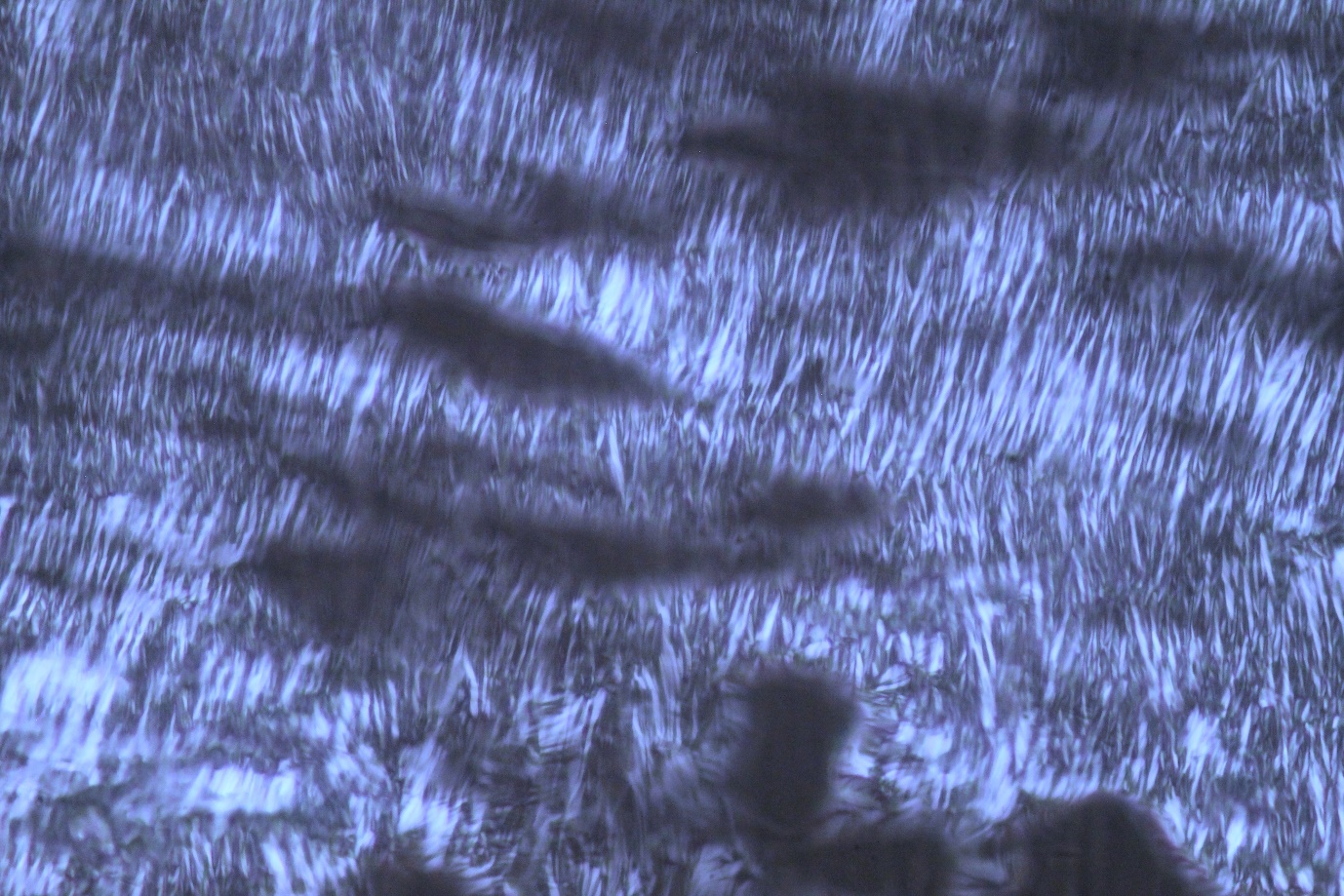


Original Image for Supplementary Figure S2: (E/L)Gl70, 48 hours; middle part of the image is included in the figure.


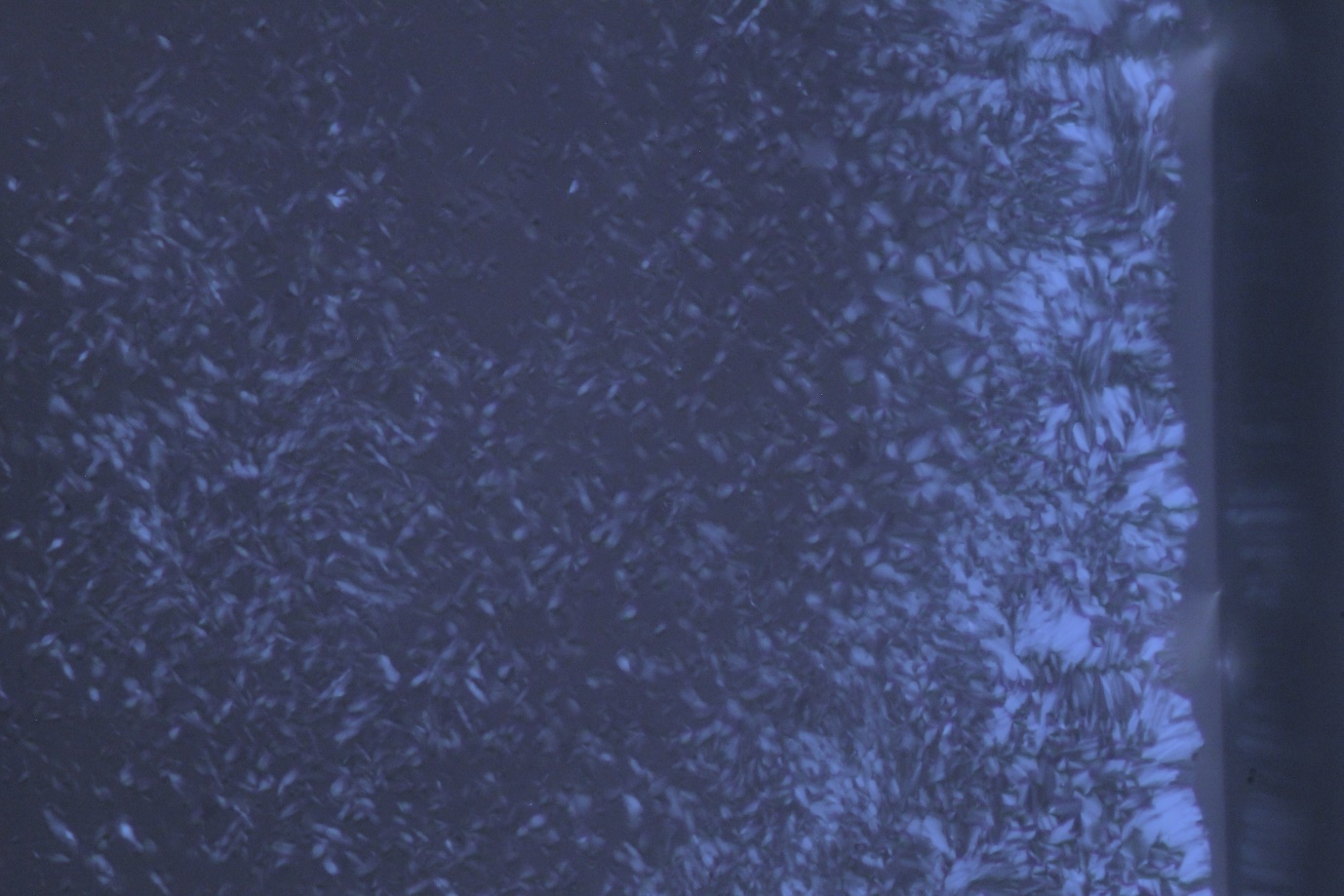


Original Image for Supplementary Figure S2: (E/L)Gl70, 10 days; middle part of the image is included in the figure.


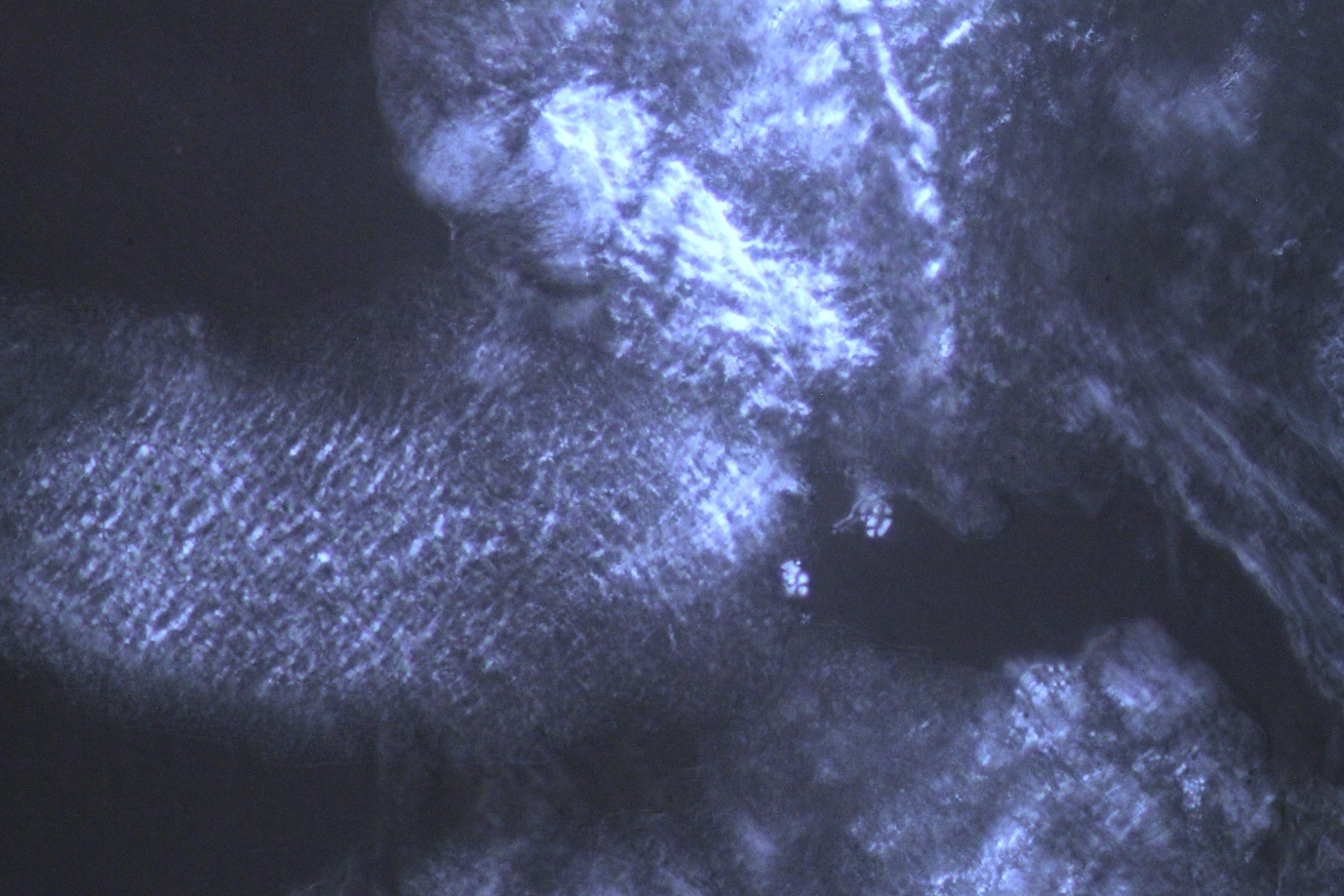


Original Image for Supplementary Figure S2: (E/L)Go80, 6 hours; middle part of the image is included in the figure.


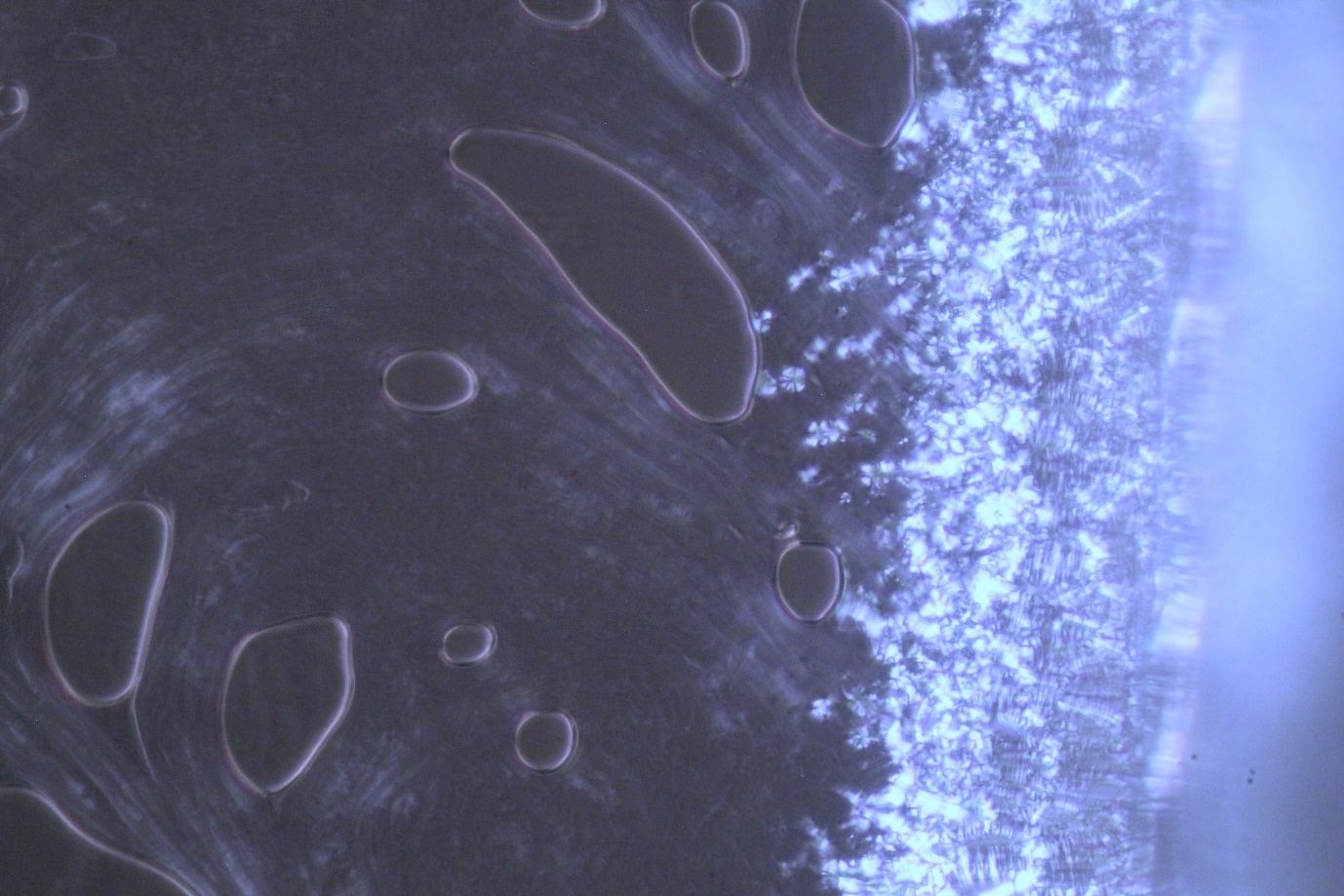


Original Image for Supplementary Figure S2: (E/L)Go80, 12 hours; middle part of the image is included in the figure.


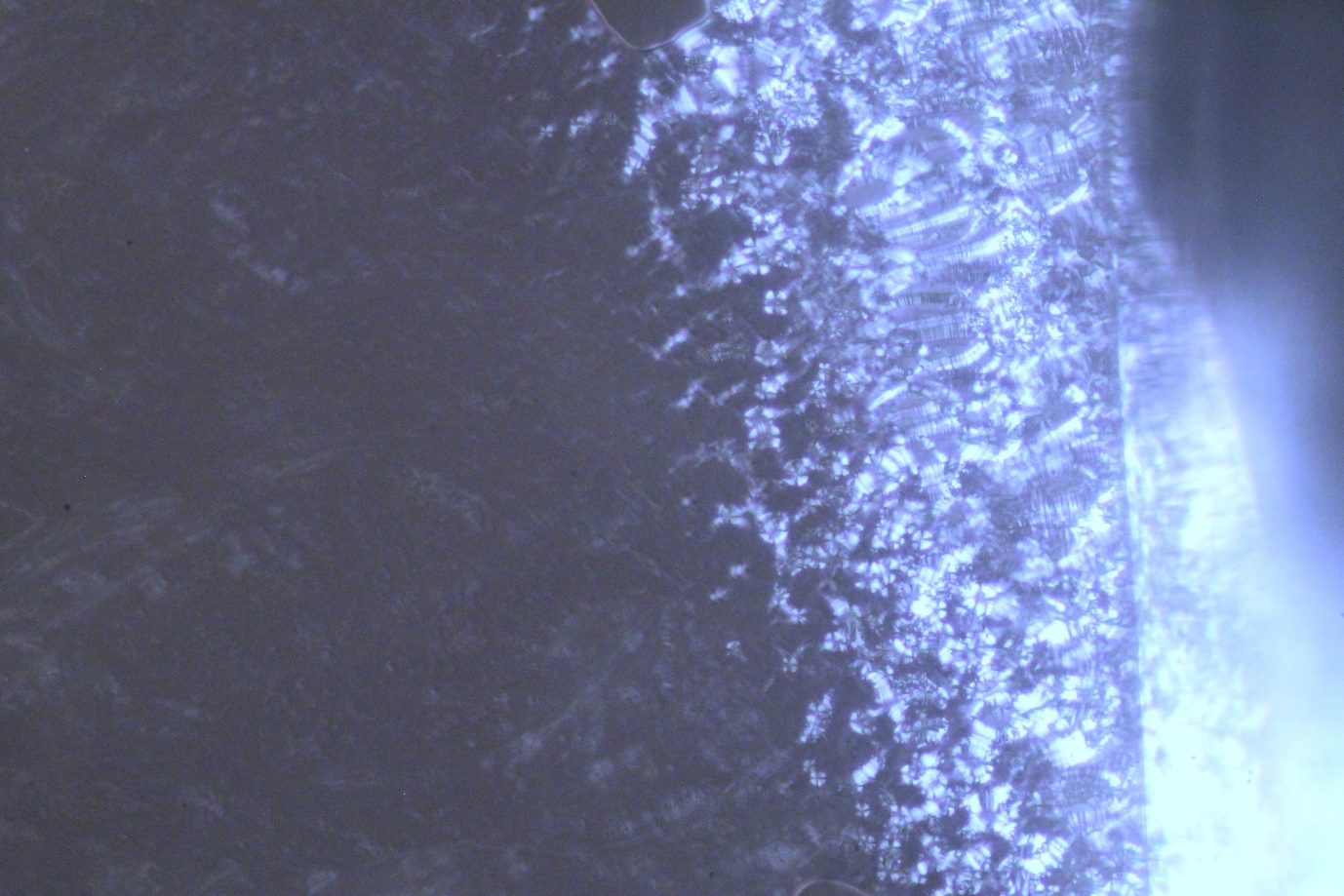


Original Image for Supplementary Figure S2: (E/L)Go80, 36 hours; middle part of the image is included in the figure.


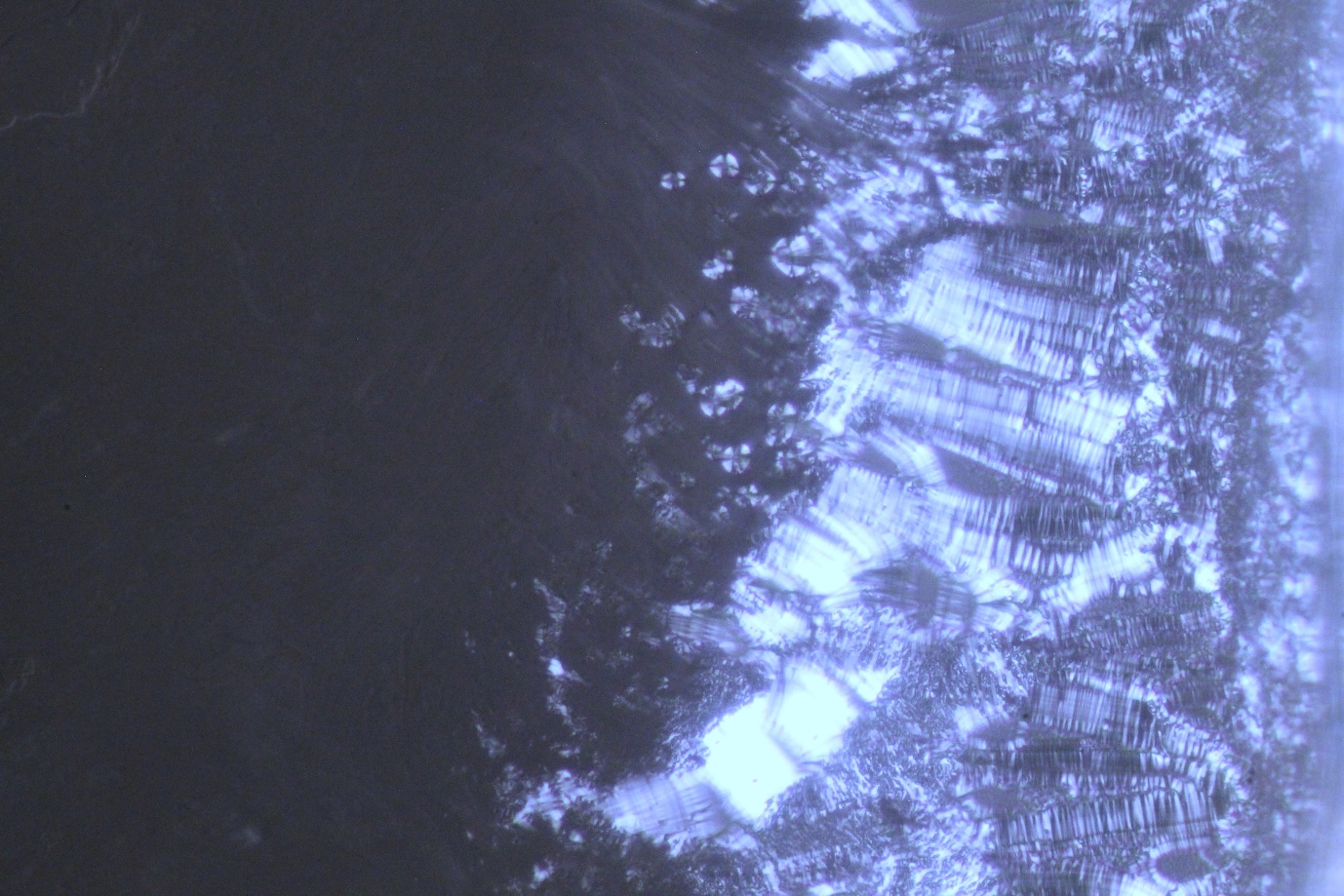


Original Image for Supplementary Figure S2: (E/L)Go80, 48 hours; middle part of the image is included in the figure.


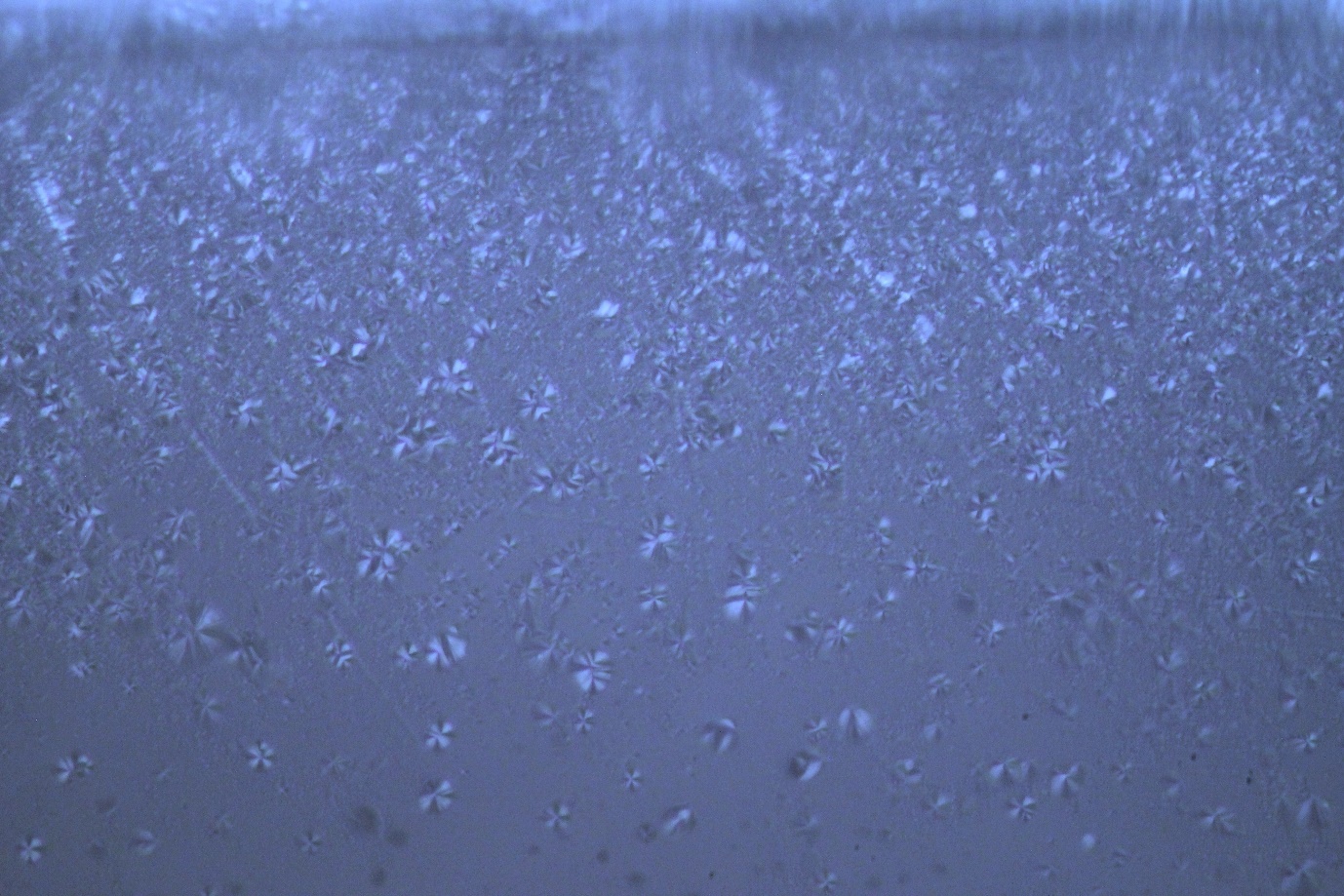


Original Image for Supplementary Figure S2: (E/L)Go80, 10 days; middle part of the image is included in the figure.


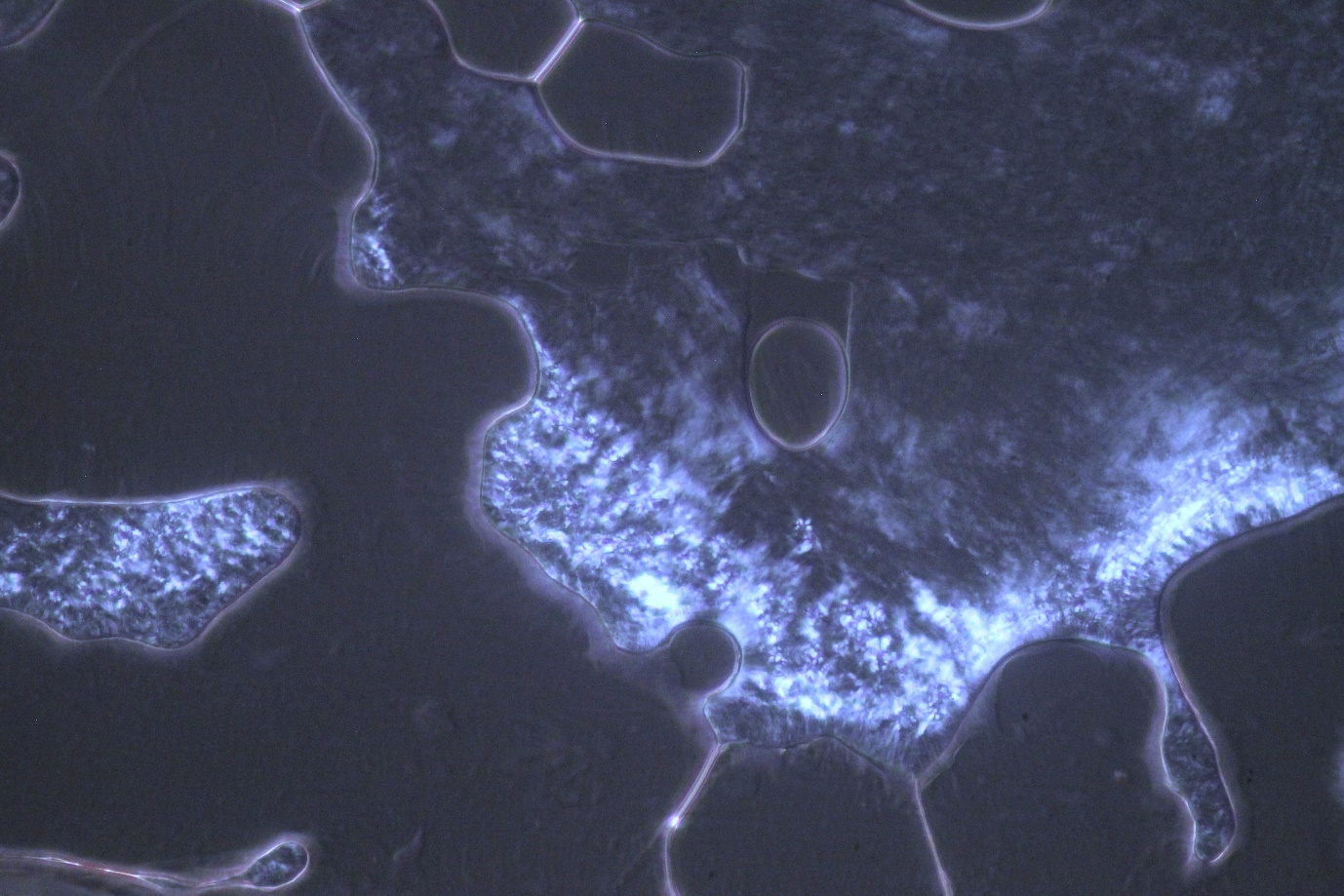


Original Image for Supplementary Figure S2: (E/L)Gl80, 6 hours; middle part of the image is included in the figure.


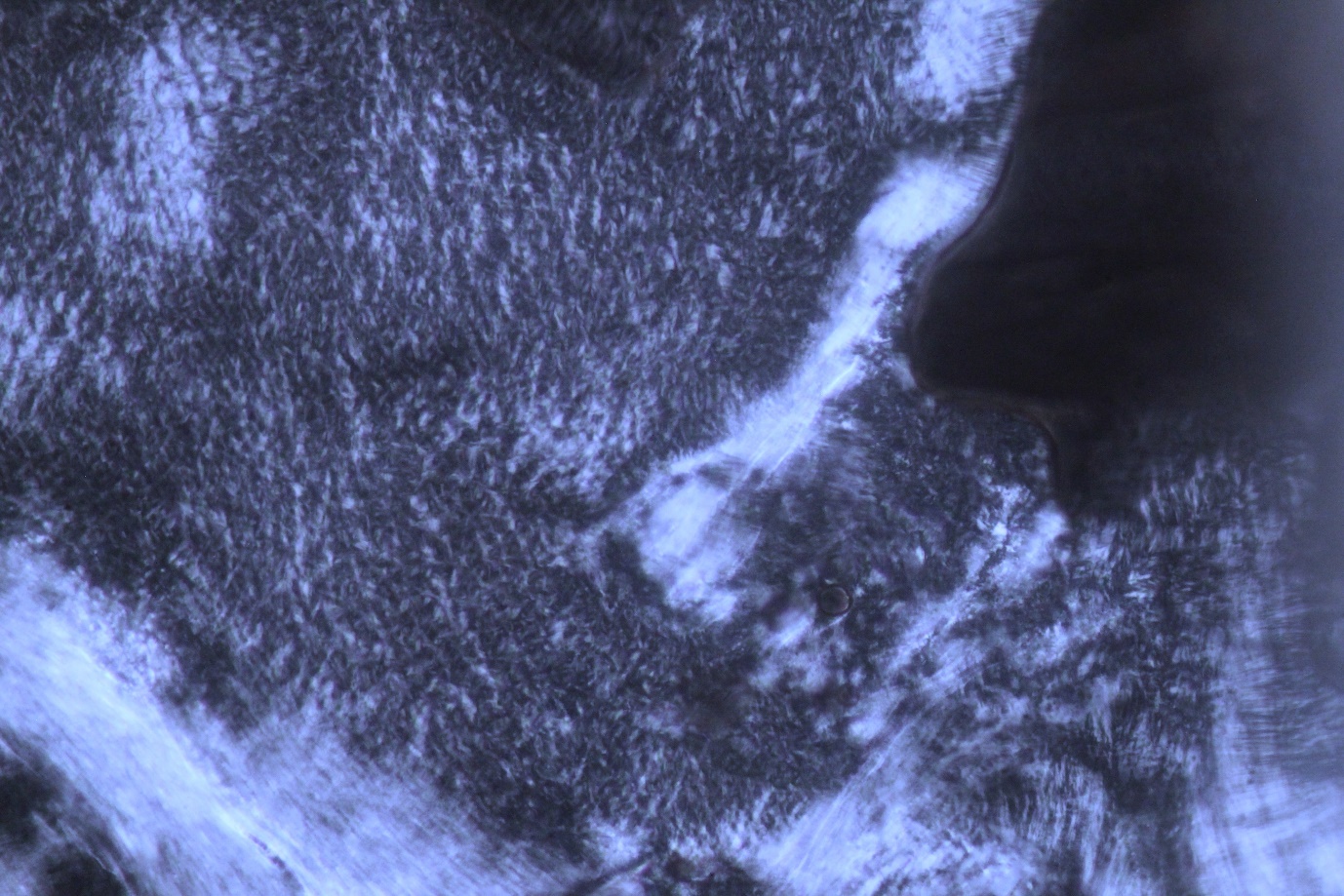


Original Image for Supplementary Figure S2: (E/L)Gl80, 12 hours; middle part of the image is included in the figure.


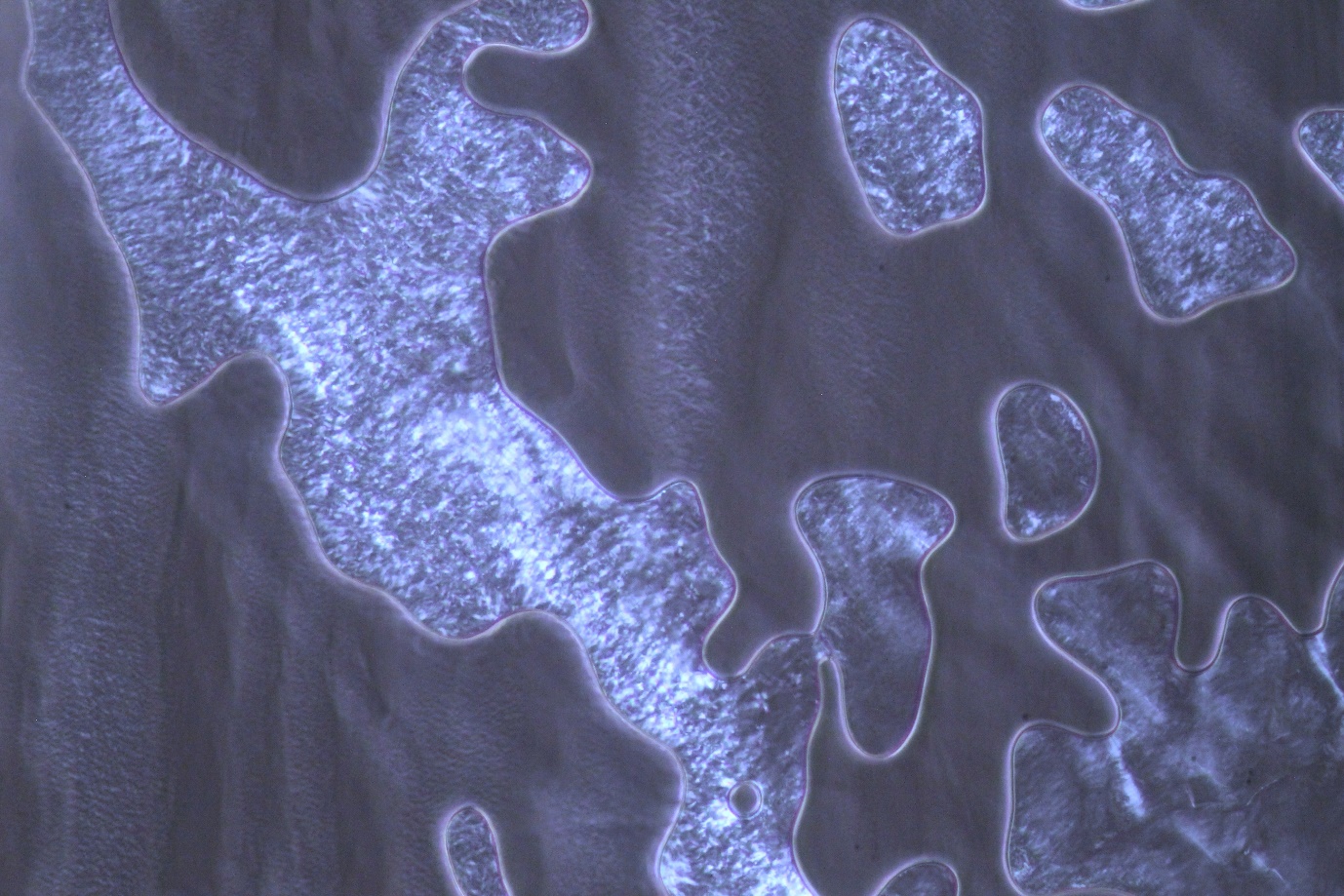


Original Image for Supplementary Figure S2: (E/L)Gl80, 36 hours; middle part of the image is included in the figure.


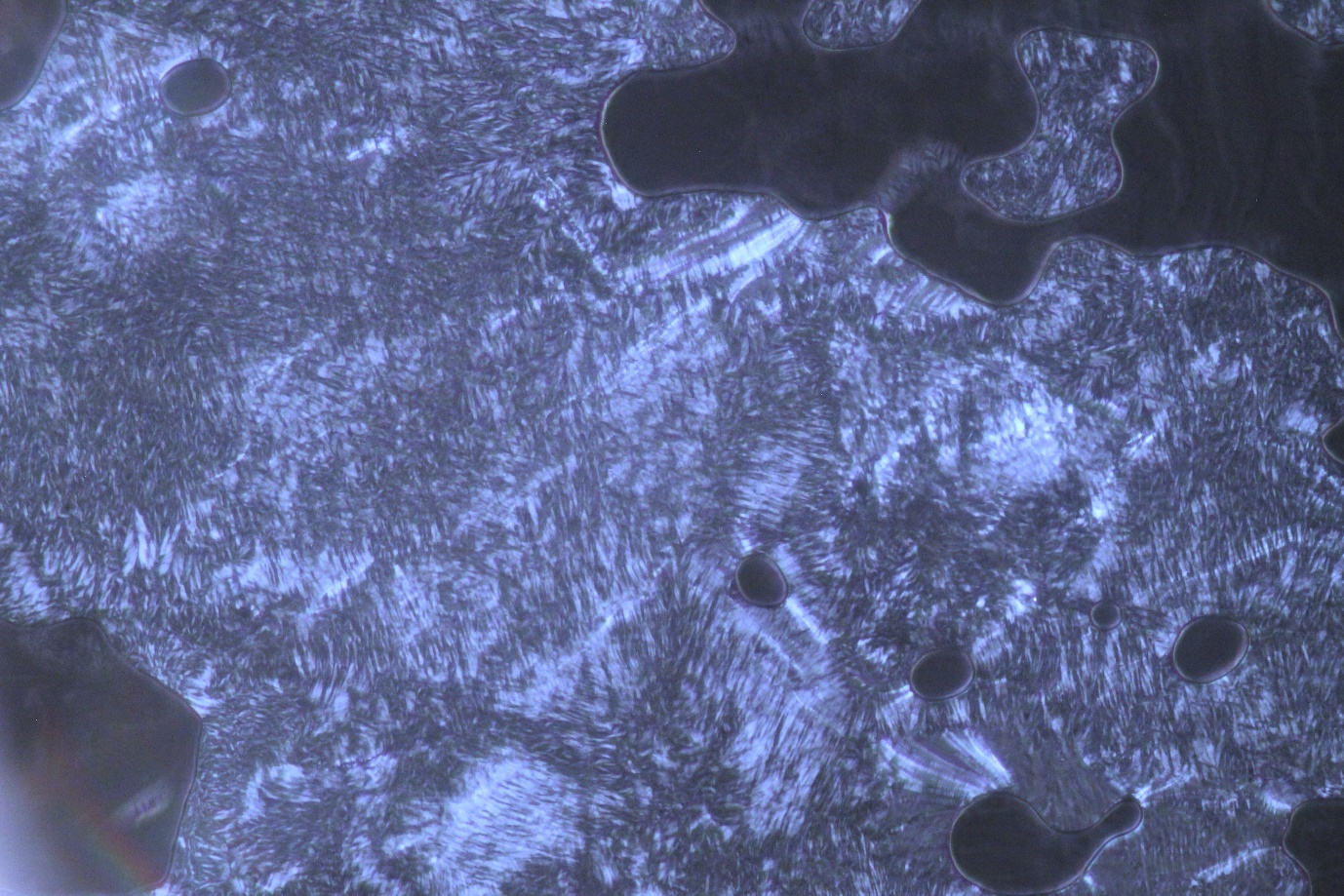


Original Image for Supplementary Figure S2: (E/L)Gl80, 48 hours; middle part of the image is included in the figure.


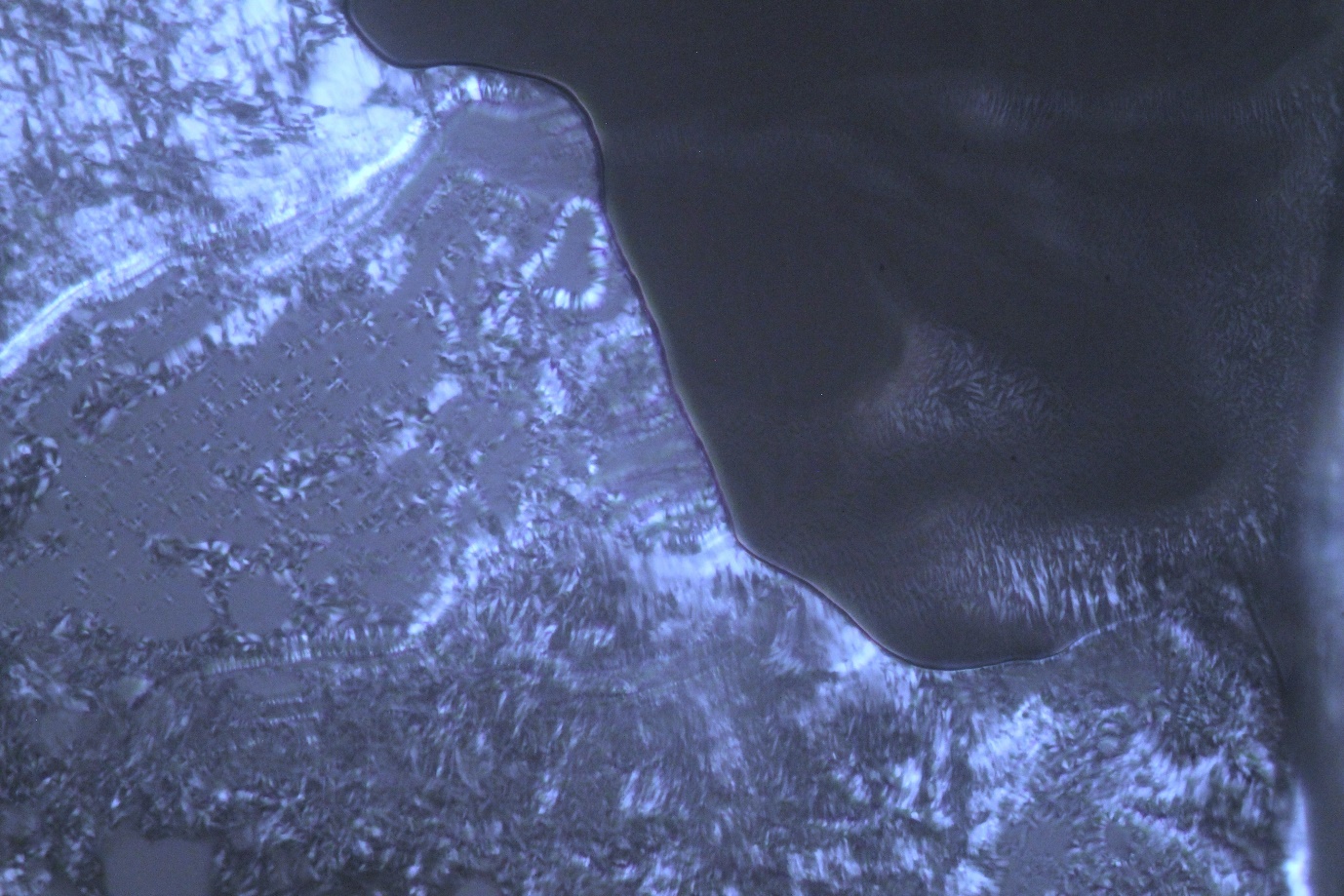


Original Image for Supplementary Figure S2: (E/L)Gl80, 10 days; middle part of the image is included in the figure.
